# Liquiritin Attenuates Pathological Cardiac Hypertrophy by Activating the PKA/LKB1/AMPK Pathway

**DOI:** 10.3389/fphar.2022.870699

**Published:** 2022-05-03

**Authors:** Xiahenazi Aiyasiding, Hai-Han Liao, Hong Feng, Nan Zhang, Zheng Lin, Wen Ding, Han Yan, Zi-Ying Zhou, Qi-Zhu Tang

**Affiliations:** ^1^ Department of Cardiology, Renmin Hospital of Wuhan University, Wuhan, China; ^2^ Hubei Key Laboratory of Metabolic and Chronic Diseases, Wuhan, China; ^3^ Department of Geriatrics, Renmin Hospital of Wuhan University, Wuhan, China

**Keywords:** liquiritin, cardiac hypertrophy, heart, AMPKα2, PKA, cAMP

## Abstract

**Background:** Liquiritin (LQ) is one of the main flavonoids extracted from the roots of *Glycyrrhiza* spp., which are widely used in traditional Chinese medicine. Studies in both cellular and animal disease models have shown that LQ attenuates or prevents oxidative stress, inflammation, and apoptosis. However, the potential therapeutic effects of LQ on pressure overload-induced cardiac hypertrophy have not been so far explored. Therefore, we investigated the cardioprotective role of LQ and its underlying mechanisms in the aortic banding (AB)-induced cardiac hypertrophy mouse model.

**Methods and Results:** Starting 3 days after AB surgery, LQ (80 mg/kg/day) was administered daily over 4 weeks. Echocardiography and pressure-volume loop analysis indicated that LQ treatment markedly improved hypertrophy-related cardiac dysfunction. Moreover, hematoxylin and eosin, picrosirius red, and TUNEL staining showed that LQ significantly inhibited cardiomyocyte hypertrophy, interstitial fibrosis, and apoptosis. Western blot assays further showed that LQ activated LKB1/AMPKα2/ACC signaling and inhibited mTORC1 phosphorylation in cardiomyocytes. Notably, LQ treatment failed to prevent cardiac dysfunction, hypertrophy, and fibrosis in AMPKα2 knockout (AMPKα2^−/−^) mice. However, LQ still induced LKB1 phosphorylation in AMPKα2^−/−^ mouse hearts. *In vitro* experiments further demonstrated that LQ inhibited Ang II-induced hypertrophy in neonatal rat cardiomyocytes (NRCMs) by increasing cAMP levels and PKA activity. Supporting the central involvement of the cAMP/PKA/LKB1/AMPKα2 signaling pathway in the cardioprotective effects of LQ, inhibition of Ang II-induced hypertrophy and induction of LKB1 and AMPKα phosphorylation were no longer observed after inhibiting PKA activity.

**Conclusion:** This study revealed that LQ alleviates pressure overload-induced cardiac hypertrophy *in vivo* and inhibits Ang II-induced cardiomyocyte hypertrophy *in vitro via* activating cAMP/PKA/LKB1/AMPKα2 signaling. These findings suggest that LQ might be a valuable adjunct to therapeutic approaches for treating pathological cardiac remodeling.

## Introduction

Heart failure (HF) is one of the most severe diseases, endangering human health and adding a substantial economic burden worldwide (Maillet et al., 2013; van Berlo et al., 2013). Thus, it is particularly necessary and urgent to develop novel therapeutic strategies for preventing the occurrence and progression of HF. Pathological cardiac hypertrophy induced by hypertension, myocardial infarction, cardiomyopathy, or structural heart diseases has been demonstrated to be an independent risk factor for HF (Maillet et al., 2013; van Berlo et al., 2013). The most prominent feature of pathological hypertrophy is contractile dysfunction, interstitial fibrosis, and re-expression of fetal cardiac genes, such as those encoding natriuretic peptides and the β-myosin heavy chain (Maillet et al., 2013; van Berlo et al., 2013). Prevention or reversal of pathological cardiac hypertrophy has thus been advocated as a strategy for treating HF.

Alterations in signal transduction pathways, such as mitogen-activated protein kinase (MAPK), adenosine monophosphate-activated protein kinase (AMPK), transforming growth factor β (TGF-β)/Smad, and Janus kinase (JAK)/STAT, have been shown to contribute to the occurrence and progression of pathological hypertrophy and HF (Frey et al., 2004; Rohini et al., 2010). Among these pathways, AMPK stands as a major cellular energy sensor and its activation was shown to protect the heart from pressure overload, ischemia- and diabetes-associated cardiac hypertrophy, and HF. The cardioprotective effects of AMPK are mediated by inhibitory phosphorylation of acetyl-CoA carboxylase (ACC), which preserves cardiomyocyte survival and function by inhibiting ATP-consuming anabolic processes (such as *de novo* lipogenesis) and enhancing ATP-generating catabolic processes (such as glycolysis and fatty acid oxidation [FAO]) (Viollet et al., 2009; Feng et al., 2018). Besides, AMPK activation inhibits mTOR/p70S6K signaling and thus blocks protein synthesis and hypertrophy in cardiomyocytes (Viollet et al., 2009; Feng et al., 2018). Genetic inhibition of AMPK aggravates cardiac hypertrophy and promotes HF progression, while overexpression or pharmacological activation of AMPK greatly attenuates development and progression of these conditions (Viollet et al., 2009; Feng et al., 2018). Liver kinase B1 (LKB1) acts as a major upstream kinase phosphorylating and activating AMPK. Cardiomyocyte-specific LKB1 knockdown leads to complete ablation of AMPKα2 activity, resulting in exacerbated pathological cardiac remodeling and impaired cardiac function (Jessen et al., 2010). Conversely, enhancing LKB1 activity improves cardiac metabolism and preserves heart function (Liu et al., 2021). Although these findings consolidated the notion of therapeutic modulation of LKB1/AMPK/mTOR signaling as a valuable strategy for preventing cardiac hypertrophy and HF, effective pharmacological treatments aimed at this end remain to be developed.

The flavonoid compound liquiritin (LQ) is one of the major constituents in Glycyrrhizae radix, which is widely used to treat various conditions and diseases including pain, cancer, cough, and allergic reactions (Zhao et al., 2008; Yang et al., 2013; Tao et al., 2016; Zhang et al., 2016; Nakatani et al., 2017; Wei et al., 2017; Ramalingam et al., 2018; Thu et al., 2021). Moreover, studies revealed that LQ administration exerts powerful pharmacological effects in heart diseases. Zhang et al. (2016) demonstrated that LQ attenuated myocardial fibrosis and inhibited the inflammatory response by blocking the activation of MAPKs and IκBα/NFκB signaling in experimental diabetic cardiomyopathy. LQ treatment was also shown to inhibit the development and progression of coronary heart disease *via* inhibiting the proliferation and migration of human vascular smooth muscle cells (Yuan et al., 2022). Our previous study demonstrated that LQ attenuated LPS-induced myocardial injury *via* AMPKα phosphorylation and activation (Mou et al., 2021). Interestingly, previous studies also indicated that LQ regulates cAMP/PKA signaling (Uto et al., 2019; Guo et al., 2020). However, whether LQ is capable of attenuating pressure overload-induced cardiac hypertrophy remains unclear. Based on the experimental findings described above, we hypothesized that LQ exerts protective effects against pathological cardiac hypertrophy by regulating cAMP/PKA/LKB1/AMPKα signaling. To test this hypothesis, in this study we evaluated the effects of LQ in a mouse model of pressure overload-induced cardiac hypertrophy induced by aortic banding (AB). In addition, the impact of LQ on cAMP/PKA/LKB1/AMPKα signaling was evaluated in AMPKα2 knockout mice subjected to AB surgery and after pharmacological inhibition of AMPK and PKA in Ang II-treated neonatal rat cardiomyocytes (NRCMs).

## Materials and Methods

### Reagents and Antibodies

LQ (white crystalline powder, purity >98%) was purchased from Shanghai Winherb Medical Tech. Co., Ltd. Compound C (CpC) (S7840) was purchased from Selleck (Shanghai, China). H-89 dihydrochloride (371963-M) was purchased from Sigma-Aldrich. Anti-LKB1 (LKB1, 3047s), anti-phospho-LKB1 (p-LKB1, 3482s), anti-Acetyl-CoA carboxylase (ACC, 3,676), anti-phospho-Acetyl-CoA carboxylase (p-ACC, 3661S), anti-mTOR (T-mTOR, 2,983), anti-phospho-mTOR (p-mTOR, 2,971), anti-GAPDH (2,118), anti-Bax (2,772), and anti-Bcl-2 (2,870) antibodies were purchased from Cell Signaling Technology (Pudong, Shanghai, China). Anti-phospho-AMPKα2 (p-AMPKα2, ab109402) and anti-AMPKα2 (T-AMPKα2, ab3760) antibodies were purchased from Abcam (Cambridge, United Kingdom). Secondary antibodies were purchased from LI-COR Biosciences (Lincoln, NE, United States). The cell counting kit 8 (CCK-8) was purchased from Dojindo Molecular Technologies (Rockville, MD, United States).

### Animals and Treatments

Adult male C57BL/6 mice aged 8–10 weeks (weight: 23.5–27.5 g) were purchased from the Institute of Laboratory Animal Science, Chinese Academy of Medical Sciences (CAMS) & Peking Union Medical College (PUMC; Beijing, China). The animals were given free access to food and drinking water. Before AB surgery, all mice were adapted for 1 week to specific pathogen-free (SPF) grade cages, with a 12 h light/dark cycle and a constant temperature (20–25°C) and humidity (50 ± 5%) environment. In order to generate pressure overload-induced cardiac hypertrophy, AB surgery was performed as described in our previous articles (Jiang et al., 2014; Zhang X. et al., 2018). Briefly, mice were anesthetized by intraperitoneal injection of pentobarbital sodium (50 mg/kg, Sigma). After adequate anesthesia, the left chest was opened through the second intercostal space and the thoracic aorta was ligated with a 27-gauge needle and a 7–0 silk suture. Successful constriction was confirmed by Doppler analysis. The sham group underwent the same surgical procedure but without aortic ligation. No AB surgery- or treatment-related deaths were recorded. After allowing recovery from surgery, mice were randomly divided into four groups: 1) Sham + vehicle (saline, *n* = 12); 2) Sham + LQ (80 mg/kg/day, *n* = 12); 3) AB + vehicle (AB + Veh, isovolumetric saline, *n* = 15); and 4) AB + LQ (80 mg/kg/day, *n* = 15). AMPKα2 knockout mice (AMPKα2^−/−^) were generated and bred in our lab as described in our previous article (Zhang N. et al., 2018). The AMPKα2^−/−^ mice were randomly divided into three groups: 1) AMPKα2^−/−^ + Sham (saline, *n* = 12); 2) AMPKα2^−/−^ + AB + Veh (saline, *n* = 15); and 3) AMPKα2^−/−^ + AB + LQ (80 mg/kg/day, *n* = 15). LQ was dissolved in 60% ethanol and then diluted with 0.9% saline into a final concentration of 8 mg/ml. LQ solution or equal volumes of the vehicle were administered daily by oral gavage over 4 consecutive weeks starting 3 days after AB or sham surgery. The dose of LQ was determined according to a previously published article (Mou et al., 2021).

### Echocardiography and Hemodynamics

After 4 weeks of treatment, cardiac functions were examined by echocardiography and pressure-volume (PV) loop analysis. Transthoracic echocardiography was performed according to the protocol reported by us (Wu et al., 2016; Zhang et al., 2021). Briefly, mice were anesthetized by isoflurane (1.5%) inhalation. Heart rate was kept between 450 and 550 beats/min by regulating the inhalational flow of isoflurane. A MyLab 30CV ultrasound system (Esaote S.P.A. Genoa, Italy) equipped with a 10-MHz line array transducer was used to examine cardiac function, including heart rate (HR), left ventricular (LV) end-diastolic diameter (LVEDd), LV end-systolic diameter (LVESd), LV fractional shortening (LVFS), and LV ejection fraction (LVEF).

PV-loop analysis was performed as described previously (Wu et al., 2016; Zhang et al., 2021). Briefly, a microtip catheter transducer (SPR-839, Millar Instruments, Houston, TX, United States) was inserted into the right carotid artery and proceeded into the LV. The Millar Pressure-Volume System (MPVS-400, Millar Instruments) was used to continuously record pressure and volume signals, and hemodynamic parameters were evaluated by PVAN data analysis software (Millar Instruments). After echocardiography and PV-loop analysis, the mice were sacrificed by cervical dislocation and the heart and lungs were weighed to calculate heart weight/body weight ratio (HW/BW, mg/g), lung weight/body weight ratio (LW/BW, mg/g), and HW/tibia length ratio (HW/TL, mg/mm). Hearts were frozen in liquid nitrogen and then stored at −80°C for downstream molecular and histological analyses.

### Cardiac Histology and Immunohistochemistry

Hearts harvested at sacrifice were immersed in 4% formaldehyde overnight after diastole arrest in 10% KCl solution. Paraffin-embedded samples were cut into 5-μm slices and hematoxylin and eosin (H&E) staining was performed to examine overall morphology and cardiomyocyte area. Picrosirius red (PSR) staining was performed to assess cardiac fibrosis. Image-Pro Plus 6.0 (Media Cybernetics, Bethesda, MD, United States) was used to estimate cross-sectional area (CSA) and calculate average collagen volume. At least 250 cardiomyocytes (50 cells per slide) were analyzed to compute cardiomyocyte surface area, and at least 60 fields of view (×200 magnification) per group were examined to quantify fibrotic area.

### Cell Culture and Treatment

Neonatal rat cardiomyocytes (NRCMs) were extracted from Sprague-Dawley rats within 3 days of birth and cultured according to our previously published method (Zhang N. et al., 2018). Briefly, the hearts were quickly removed, digested, centrifuged, filtered, and cell density was calculated with a hemocytometer. NRCMs were seeded into 6-, 24-, or 96-well plates, according to the experimental requirements, in Dulbecco’s Modified Eagle’s Medium (DMEM; Gibco, C11995) with 15% fetal bovine serum (FBS). (E)-5-(2-bromovinyl)-2’-deoxyuridine (BrdU, 0.1 mM) was added to the culture media for 24 h to inhibit fibroblast growth. To determine optimal LQ dosage, NRCMs were treated with different concentrations of LQ and viability assessed by CCK-8 analysis. Preincubations with CpC (10 μM), H-89 (1 μM), and LQ (80 μM) were carried out for 2 h before Ang II treatment (1 mM; 48 h). Six experimental groups were defined: 1) control (CON); 2) CpC; 3) Ang II; 4) Ang II + LQ; 5) Ang II + CpC; and 6) Ang II + LQ + CpC.

### Immunofluorescence

Immunofluorescence was performed according to a previously published protocol (Zhang X. et al., 2018). Briefly, NRCMs cultured on coverslips were pretreated with or without LQ (80 μM) and then stimulated with 1 mM Ang II for 48 h. Before staining, the NRCMs were fixed in 4% formaldehyde and permeabilized in 0.2% Triton X-100. Then the cells were blocked with 10% goat serum for 1 h at 37°C, stained with α-actinin (1:100; Cell Signaling Technology, 69758S), and incubated with Alexa Fluor 488-goat anti-mouse secondary antibody (1:200). Finally, the cells were stained with 4,6-diamidino-2-phenylindole (DAPI) for nuclei observation. An Olympus DX51 fluorescence microscope (Olympus Corporation, Tokyo, Japan) was used to capture the images, and Image-Pro Plus 6.0 was used to calculate cross-sectional areas. At least 100 myocytes were outlined in each group.

### TUNEL Staining

A Terminal Deoxynucleotidyl Transferase-mediated dUTP Nick-End-Labeling (TUNEL) staining kit (Millipore, Billerica, MA, United States) was used to examine cellular apoptosis according to the manufacturer’s instructions. The images were analyzed by Image-Pro Plus 6.0.

### Cellular cAMP and PKA Activity Assays

Cell extracts were prepared using commercial kits according to the manufacturer’s instructions, as described in our previous article (Wu et al., 2020). cAMP levels were estimated using a competitive ELISA kit (#4339; Cell Signaling Technology). PKA activity was detected using a PKA Colorimetric Activity Kit (EIAPKA; Thermo Fisher Scientific, Waltham, MA, United States) by measuring the phosphorylation level of a specific synthetic PKA substrate.

### Quantitative Real-Time PCR

The transcriptional levels of cardiac hypertrophy- and fibrosis-related genes were examined by real-time reverse transcriptase-polymerase chain reaction (RT-PCR). Total RNA was extracted from hearts or cells using TRIzol reagent (Invitrogen, Carlsbad, CA, United States). The concentration and purity of the extracted RNA were evaluated by A260/A280 and A260/A230 absorbance ratios using an ultraviolet spectrophotometer (NanoDrop 2000; Thermo Fisher Scientific). 2 μg of total RNA was reversely transcribed into cDNA using oligo (dT) primers with a commercial kit (Roche, Mannheim, Germany). SYBR green was used to detect amplification of target genes. Relative expression of target genes was determined by normalization against GAPDH. The primers used in this study are shown in Table 1.

**TABLE 1:** Sequences for the primers used in the qRT-PCR assays.

Gene name	Forward (5′−3′)	Reverse (5′−3′)
ANP-M	ATT​GAC​AGG​ATT​GGA​GCC​CAG	TCA​AGC​AGA​ATC​GAC​TGC​CTT
BNP-M	TTT​GGG​CTG​TAA​CGC​ACT​GA	CAC​TTC​AAA​GGT​GGT​CCC​AGA
α-MHC-M	AGG​TGG​ACC​TGA​TCA​TGG​AG	ATA​CCG​GAG​ATC​ATG​CAA​GC
β-MHC-M	CCG​AGT​CCC​AGG​TCA​ACA​A	CTT​CAC​GGG​CAC​CCT​TGG​A
Collagen I-M	AGC​ACG​TCT​GGT​TTG​GAG​AG	GAC​ATT​AGG​CGC​AGG​AAG​GT
CTGF-M	AGA​CCT​GTG​CCT​GCC​ATT​AC	ACG​CCA​TGT​CTC​CGT​ACA​TC
Fibronectin-M	GAC​CCT​TAC​ACG​GTT​TCC​CA	AAG​CAC​TGG​CAT​GTG​AGC​TT
α-SMA-M	CCA​GCC​ATC​TTT​CAT​TGG​GAT	ACA​GGA​CGT​TGT​TAG​CAT​AGA​G
TGFβ1-M	GGT​GGT​ATA​CTG​AGA​CAC​CTT​G	CCC​AAG​GAA​AGG​TAG​GTG​ATA​G
GAPDH-M	ACT​CCA​CTC​ACG​GCA​AAT​TC	TCT​CCA​TGG​TGG​TGA​AGA​CA
ANP-R	CGG​TAC​CGA​AGA​TAA​CAG​CCA	TCA​CCA​CCT​CTC​AGT​GGC​AA
α-MHC-R	TGA​CGT​CAC​CTC​CAA​CAT​GG	AGC​TGG​GAA​ATC​AGT​GCC​TC
β-MHC-R	AGT​GAA​GAG​CCT​CCA​GAG​TTT​G	GTT​GAT​GAG​GCT​GGT​GTT​CTG​G
Collagen I-R	GAG​AGA​GCA​TGA​CCG​ATG​GAT​T	TGG​ACA​TTA​GGC​GCA​GGA​A
CTGF-R	AGA​CAC​ATT​TGG​CCC​TGA​CC	TCT​TAG​AAC​AGG​CGC​TCC​AC
GAPDH-R	GAC​ATG​CCG​CCT​GGA​GAA​AC	AGC​CCA​GGA​TGC​CCT​TTA​GT

ANP, Atrial natriuretic peptide; BNP, brain natriuretic peptide; α-MHC, alpha-myosin heavy chain; β-MHC, beta myosin heavy chain; CTGF, connective tissue growth factor; α-SMA, α-smooth muscle actin; TGFβ, transforming growth factor beta; GAPDH, glyceraldehyde-3-phosphate dehydrogenase.

### Western Blotting

Western blots were performed according to our previous study (Zhang X. et al., 2018). Heart tissues and cultured NRCMs were lysed with RIPA buffer, and protein concentrations were quantified using a BCA assay kit (Thermo Fisher Scientific). Equal amounts of protein lysates were loaded into 8%, 10%, or 12% SDS-PAGE gels and transferred to polyvinylidene difluoride (PVDF) membranes (Millipore). After 1 h blocking with 5% nonfat milk, the blots were incubated with primary antibodies overnight at 4°C. The next day, after washing three times with Tris-buffered saline containing 0.1% Tween 20, the blots were incubated at room temperature for 1 h with peroxidase-labeled secondary antibody (LI-COR Biosciences, 1:10,000), and signals detected using an ECL kit (Bio-Rad, United States). For semi-quantitative analysis, immunoblot signals were normalized to total protein or GAPDH. An Odyssey Infrared Imaging System (LI-COR Biosciences) was used to scan membranes, and Image Lab software (v3.0; Bio-Rad) was used to for densitometry.

### Statistical Analysis

All data are presented as the mean ± standard error derived from at least 5 replicates per experimental condition. GraphPad Prism 8.0.1 (GraphPad Software, La Jolla, CA, United States) was used to perform statistical analyses and to generate the graphs. One-way ANOVA followed by Tukey’s post hoc test was used to test statistical differences among multiple groups. *p* < 0.05 was considered significant. Whenever possible, experiments were conducted in a randomized and blinded manner.

## Results

### Liquiritin Treatment Improves Cardiac Dysfunction and Attenuates Pressure Overload-Induced Cardiac Hypertrophy in Mice

**FIGURE 1:**
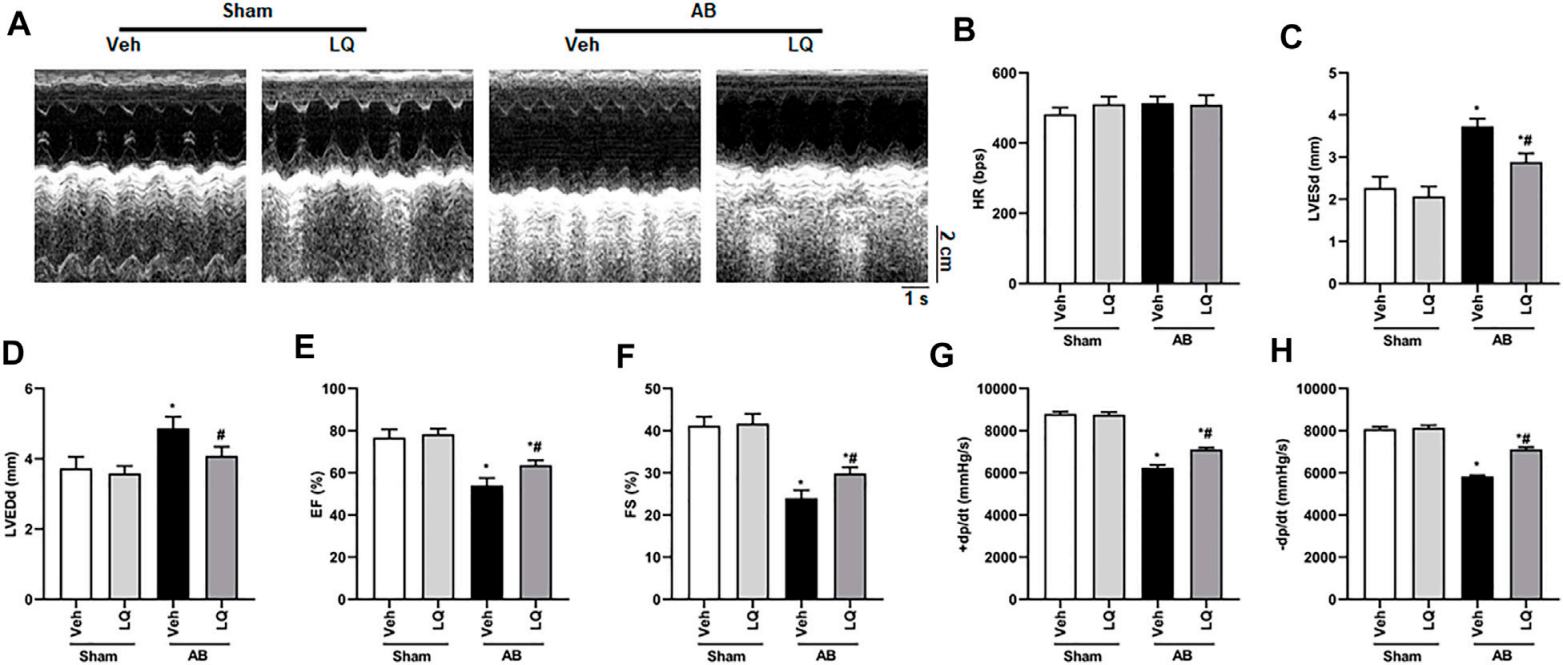
Liquiritin attenuates hypertrophy-related cardiac dysfunction. Echocardiography and PV loop analyses were performed 4 weeks after AB (*n* = 10) or sham (*n* = 6) surgery. **(A)** Representative echocardiographic images. **(B)** Heart rate (HR). **(C)** Left-ventricular end-systolic diameter (LVESd). **(D)** Left-ventricular end-diastolic diameter (LVEDd). **(E)** Ejection fraction (EF). **(F)** Fractional shortening (FS). **(G)** Left ventricular maximum rate of pressure rise (dP/dt max). **(H)** Left ventricular maximum rate of LV pressure decay (dP/dt min). Data are presented as the mean ± SEM. One-way ANOVA was performed to examine statistical differences among different groups. *p < 0.05 vs. Sham + Veh; #*p* < 0.05 vs. AB + Veh.

**FIGURE 2:**
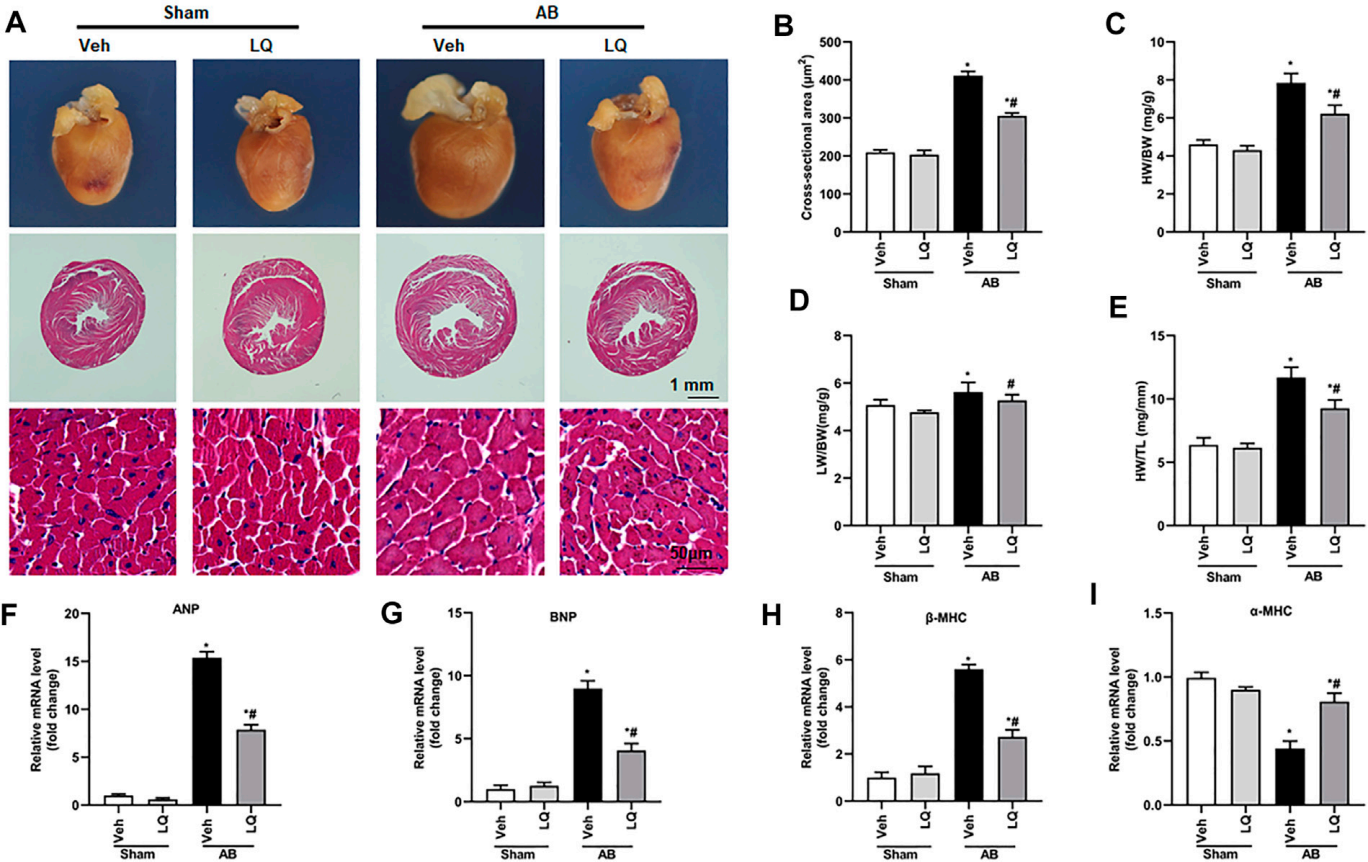
Liquiritin attenuates cardiac hypertrophy *in vivo*. **(A)** Representative gross heart and H&E images. **(B)** Statistical quantification of cross-sectional areas determined by H&E staining (*n* = 6). **(C–E)** Heart weight/body weight (HW/BW), lung weight/body weight (LW/BW), and heart weight/tibia length (HW/TL) measurements (*n* = 6). Relative mRNA levels of **(F)** ANP, **(G)** BNP, **(H)** β-MHC, and **(I)** α-MCH (*n* = 6). mRNA expression of target genes was normalized to GAPDH. Data are presented as the mean ± SEM. One-way ANOVA was performed to examine statistical differences among different groups. *p < 0.05 vs. Sham + Veh; #*p* < 0.05 vs. AB + Veh.

*In vivo* assessment of the potential beneficial effects of LQ on cardiac hypertrophy was conducted in mice subjected to AB-induced pressure overload. Following AB or sham surgery, mice were treated during 4 weeks with LQ or vehicle (Veh; saline), and echocardiography was performed to test cardiac function (Figure 1A). The heart rates showed no significant differences among the different groups (Figure 1B). In control mice (AB + Veh), cardiac dysfunction was evidenced by increased LVESd and LVEDd (Figures 1C,D) and decreased LVEF and LVFS (Figures 1E,F). However, LQ treatment significantly decreased LVESd and LVEDd and restored LVEF and LVFS (Figures 1C–F). PV-loop analysis further indicated significantly decreased LV maximum and minimum rates of pressure change (dP/dt max and dP/dt min, respectively) in AB + Veh mice were significantly improved following LQ treatment. In turn, no significant differences in these variables were detected between the Sham groups (Figures 1G,H). Of note, LQ treatment had no significant effect on end-systolic pressure (ESP) and maximum pressure (P_max_) (Supplementary Figures S1A,B). These data indicated that LQ treatment prevents pressure overload-induced cardiac dysfunction in mice without directly regulating blood pressure. Compared to the Sham groups, increased heart size, cardiac cross-sectional area (CSA), and heart weight/body weight (HW/BW), lung weight/BW (LW/BW), and HW/tibial length (HW/TL) ratios were observed in the AB + Veh group, and these changes were significantly attenuated or prevented in LQ-treated mice (Figures 2A–E).

Cardiac mRNA levels of atrial natriuretic peptide (ANP), B-type natriuretic peptide (BNP), and β-myosin heavy chain (β-MHC) were increased, while α-myosin heavy chain (α-MHC) mRNA expression was decreased, in the AB + Veh group compared to the Sham groups. In contrast, marked inhibition of ANP, BNP, and β-MHC mRNA expression and restored α-MHC expression were observed in AB + LQ mice (Figures 2F–I). These data illustrated that LQ effectively inhibited the expression of hypertrophy-associated genes in AB-treated mice.

### Liquiritin Attenuates Pressure Overload-Induced Cardiac Fibrosis

**FIGURE 3:**
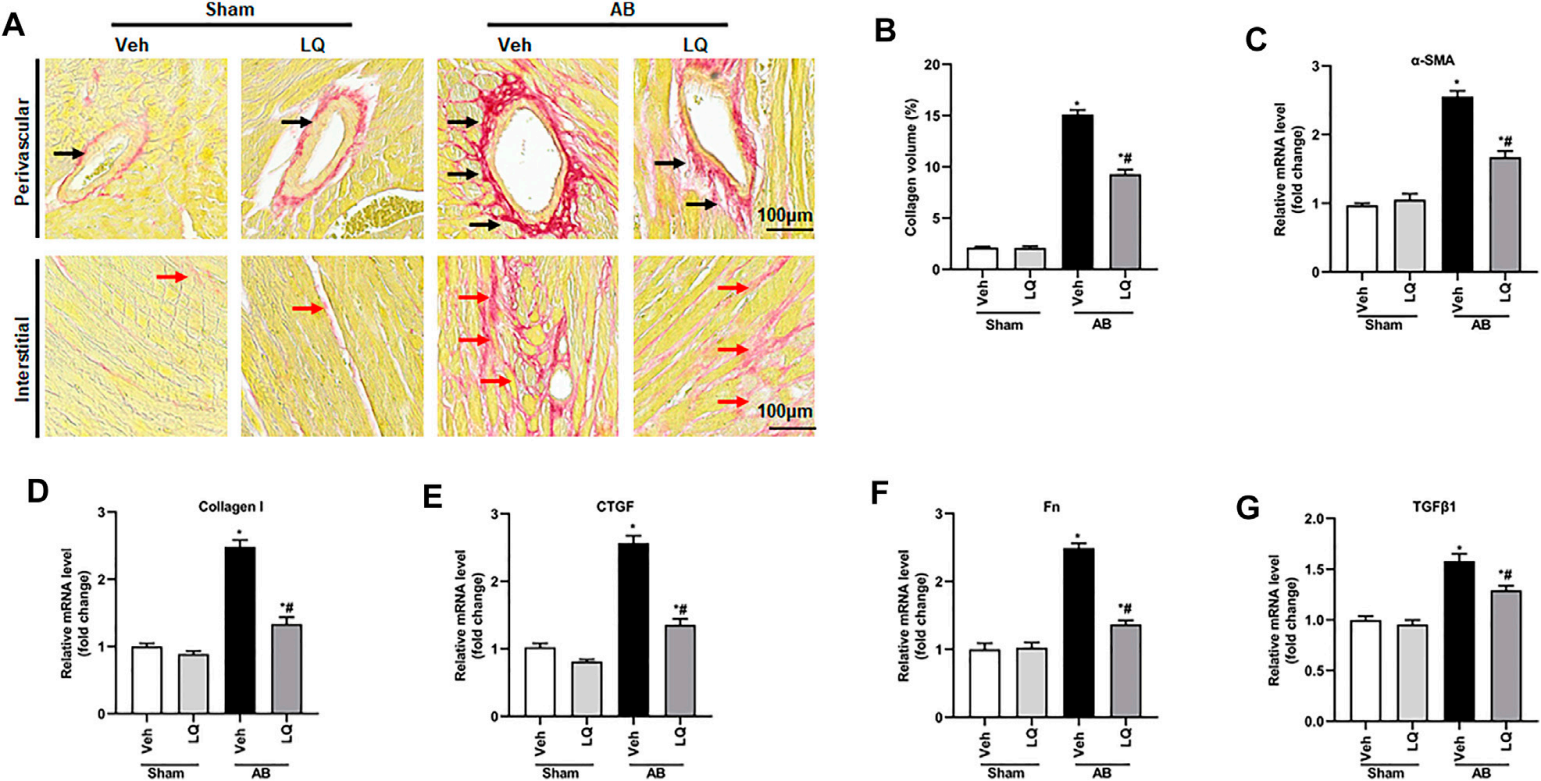
Liquiritin attenuates cardiac fibrosis *in vivo*. **(A)** Representative images of picrosirius red (PSR) staining to assess interstitial (red arrows) and perivascular (black arrow) fibrosis. **(B)** Estimation of cardiac fibrosis area according to PSR staining (*n* = 6). Relative mRNA levels of **(C)** α-SMA, **(D)** collagen I, **(E)** CTGF, **(F)** Fn, and **(G)** TGFβ1 (*n* = 6). mRNA expression of target genes was normalized to GAPDH. Data are presented as the mean ± SEM. One-way ANOVA was performed to examine statistical differences among different groups. *p < 0.05 vs. Sham + Veh; #*p* < 0.05 vs. AB + Veh.

Myocardial fibrosis, characterized by collagen accumulation and extracellular matrix deposition, is an essential feature of pathological cardiac hypertrophy (Ai et al., 2010). Cardiac PSR staining showed significant interstitial and perivascular fibrosis in the AB + Veh group compared to the Sham groups, whereas reduced myocardial fibrosis was seen in the AB + LQ (Figures 3A,B). Consistently, the mRNA levels of fibrotic markers, including α-smooth muscle actin (α-SMA), collagen I, connective tissue growth factor (CTGF), fibronectin (Fn), and transforming growth factor β1 (TGF-β1), were significantly upregulated in the AB + Veh group compared to the Sham groups. However, the expression of these pro-fibrotic markers was significantly inhibited in the AB + LQ group (Figures 3C–G). These data indicated that LQ treatment remarkably suppresses cardiac fibrosis induced by pressure overload in mice.

### Liquiritin Reduces Pressure Overload-Induced Cardiomyocyte Apoptosis *in Vivo*

**FIGURE 4:**
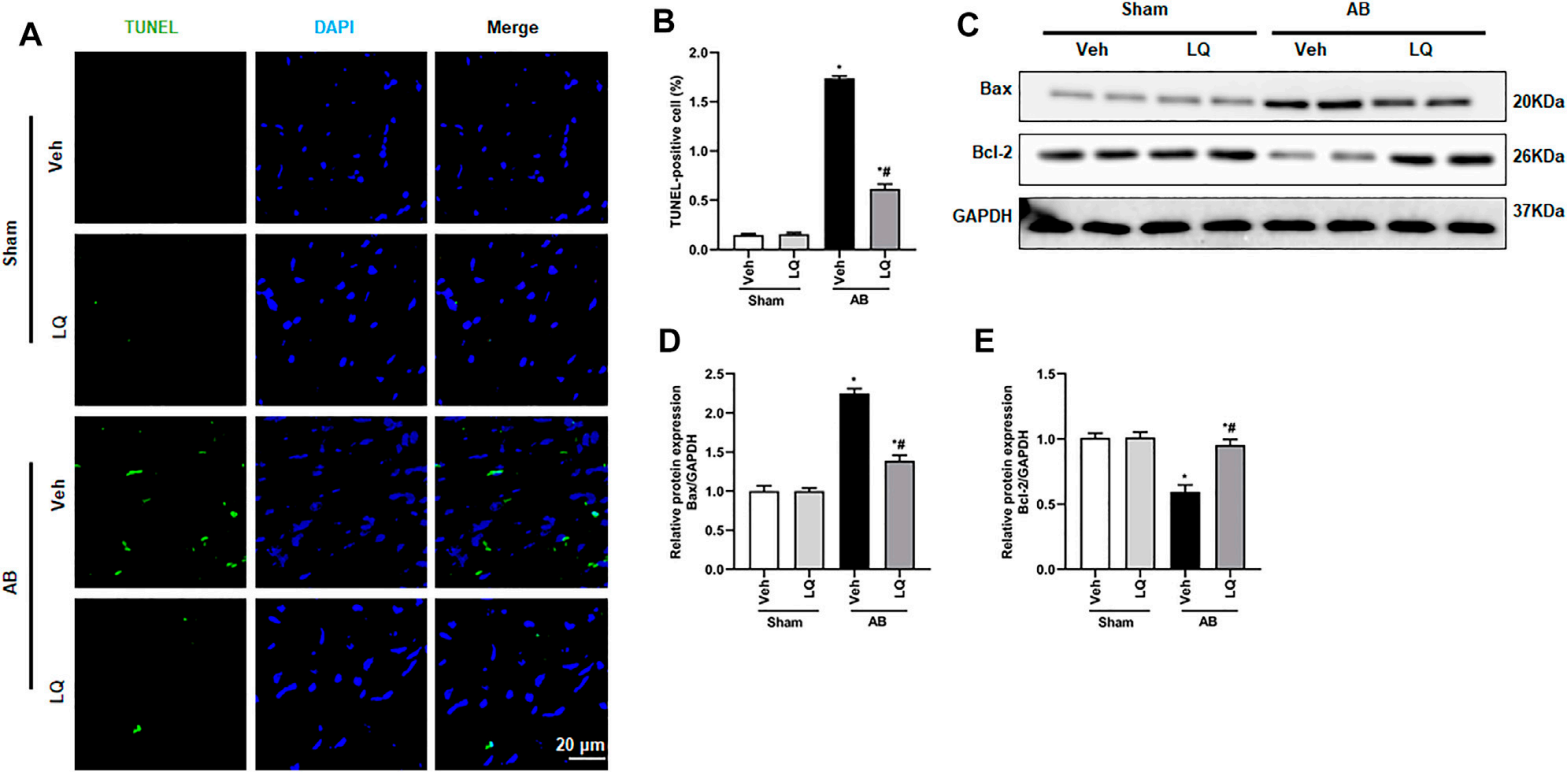
Liquiritin reduces cardiomyocyte apoptosis *in vivo*. **(A)** Representative images of TUNEL staining to examine myocardial apoptosis. **(B)** Quantification of TUNEL-positive cells (*n* = 6). **(C)** Representative immunoblots for Bax and Bcl-2. **(D,E)** Densitometric analysis of Bax and Bcl-2 expression. Bax2 and Bcl-2 signals were normalized to those of GAPDH. Data are presented as the mean ± SEM. One-way ANOVA was performed to examine statistical differences among different groups. *p < 0.05 vs. Sham + Veh. #*p* < 0.05 vs. AB + Veh.

Previous studies demonstrated that pressure overload and Ang II treatment induce cardiomyocyte apoptosis (Ma et al., 2016). Based on evidence indicated that LQ protects against oxidative stress and neuronal apoptosis triggered by cerebral ischemia/reperfusion (Wang et al., 2008), we performed TUNEL staining to evaluate whether LQ counteracts cardiac hypertrophy-related cardiomyocyte death. Results showed that cardiomyocyte apoptosis was markedly inhibited in the AB + LQ group compared to the AB + Veh group (Figures 4A,B). Moreover, significant upregulation of Bax and decreased Bcl-2 expression were detected by western blotting in heart samples from the AB + Veh group compared to the Sham groups, and these changes were reversed in the AB + LQ group (Figures 4C–E). These results showed that LQ treatment prevents pressure overload-induced apoptosis in the mouse heart.

### Liquiritin Promotes LKB1/AMPKα/ACC Pathway Activation and Inhibits mTORC1 Phosphorylation

**FIGURE 5:**
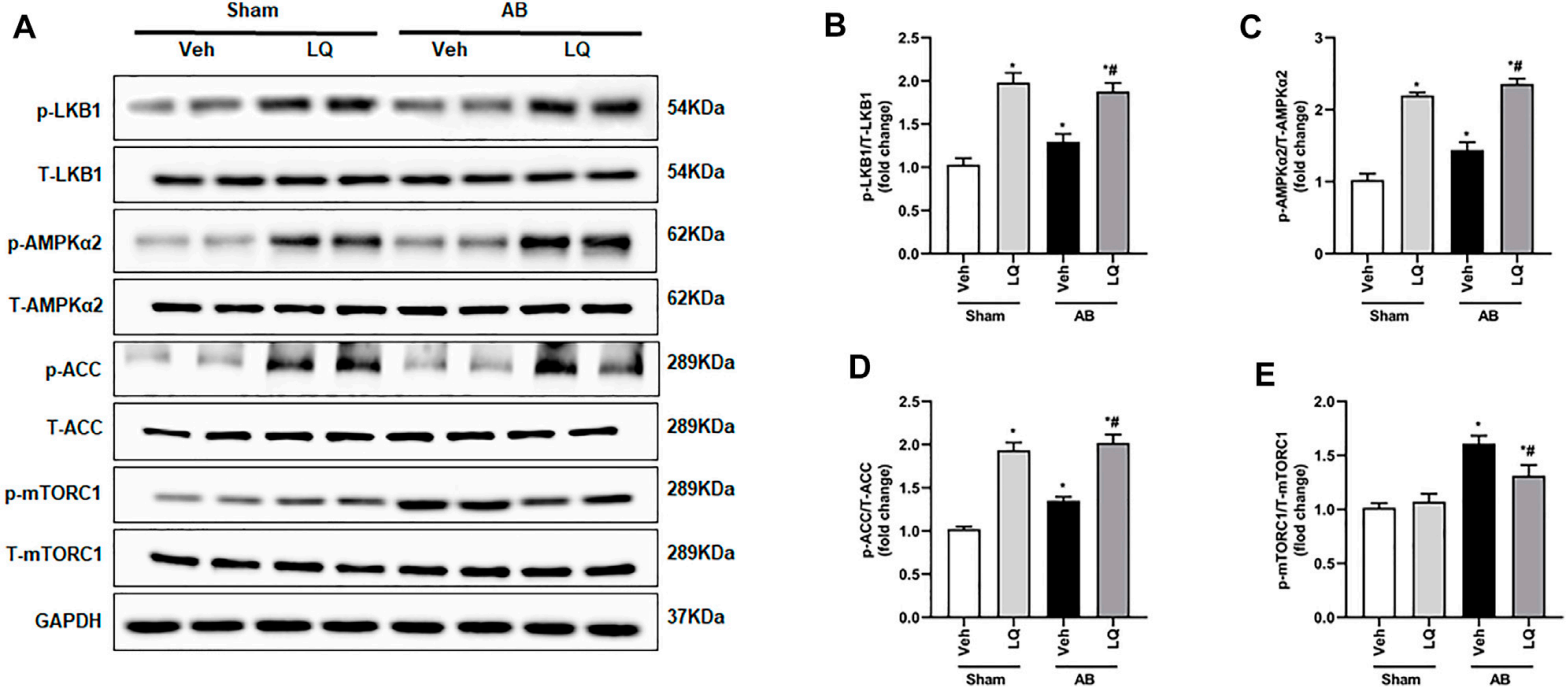
Liquiritin activates the LKB1/AMPKα/ACC/mTORC1 signaling pathway in the myocardium. **(A)** Representative immunoblots for phosphorylated LKB1 (p-LKB1), total LKB1 (T-LBK1), p-AMPKα2, T-AMPKα2, p-Acetyl-CoA carboxylase (p-ACC), T-ACC, p-mTORC1, and T-mTORC1. Relative expression of **(B)** p-LKB1/T-LKB1, **(C)** p-AMPKα2/T-AMPKα2, **(D)** p-ACC/T-ACC, and **(E)** p-mTORC1/T-mTORC1 (*n* = 6). Protein expression levels were normalized to those of GAPDH. Data are presented as the mean ± SEM. One-way ANOVA was performed to examine statistical differences among different groups. *p < 0.05 vs. Sham + Veh; #*p* < 0.05 vs. AB + Veh.

The AMPKα/mTOR pathway is an important regulator of pathological cardiac hypertrophy (Nakamura and Sadoshima, 2018). A previous study indicated that LQ-mediated AMPKα activation attenuates cardiac dysfunction in a mouse model of septic cardiomyopathy (Mou et al., 2021). Thus, we examined whether LQ treatment affects the phosphorylation status of LKB1 (a serine–threonine kinase that directly phosphorylates and activates AMPKα), AMPKα2, and two AMPKα targets (i.e., mTORC1 and ACC) known to influence the heart’s hypertrophic response. Western blot assays on cardiac tissues showed markedly enhanced LKB1, AMPKα2, and ACC phosphorylation and decreased mTORC1 phosphorylation in the AB + LQ group compared to the AB + Veh group (Figures 5A–E). These data suggested that LQ attenuates pressure overload-induced cardiac hypertrophy through LKB1/AMPKα2/ACC pathway activation and inhibition of mTORC1 signaling.

### Liquiritin-Mediated Cardioprotection is Blunted in AMPKα2 Knockout Mice

**FIGURE 6:**
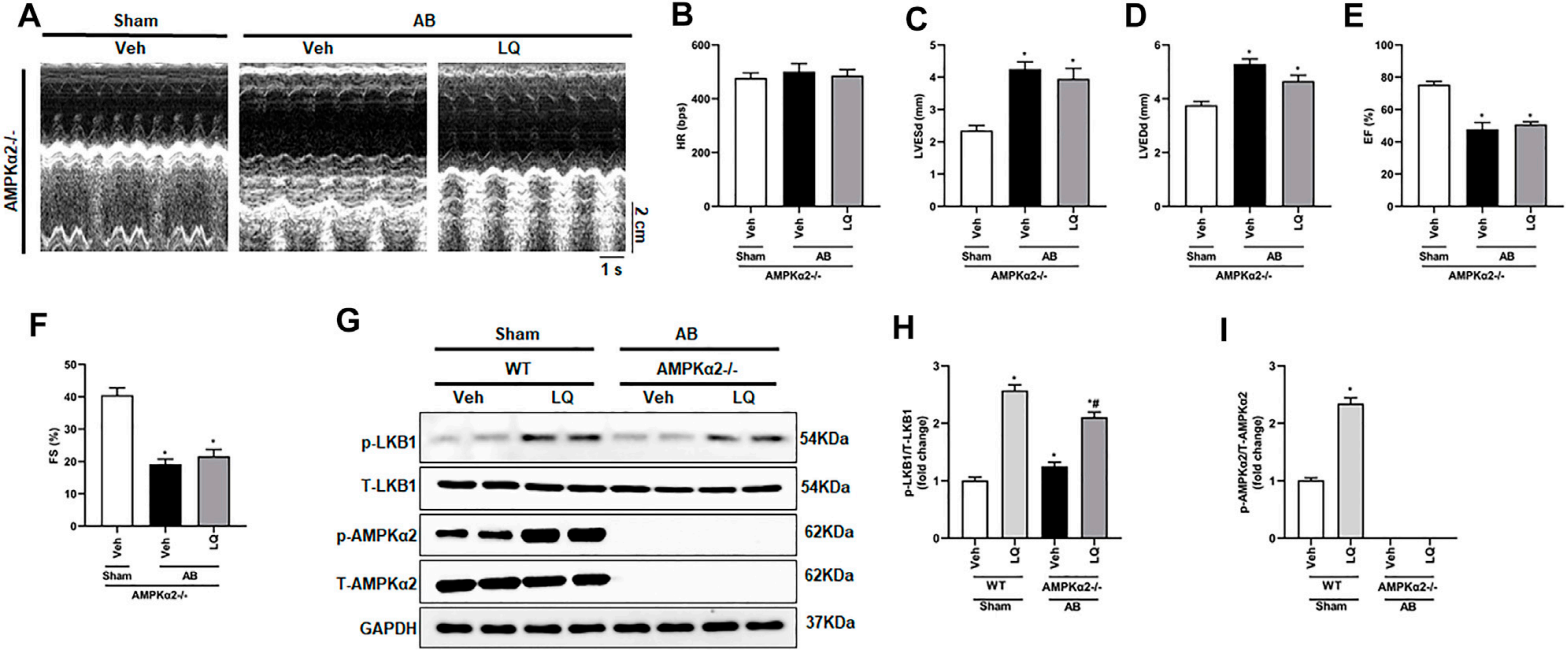
Liquiritin does not prevent hypertrophy-related cardiac dysfunction in AMPKα2 knockout mice. **(A)** Representative echocardiographic images. **(B)** Heart rate (HR). **(C)** Left-ventricular end-systolic diameter (LVESd). **(D)** Left ventricular end-diastolic diameter (LVEDd). **(E)** Ejection fraction (EF). **(F)** Fractional shortening (FS); (Sham, *n* = 6; AB, *n* = 8). **(G)** Representative immunoblots depicting cardiac expression of AMPKα2 and LKB1 in AMPKα2^−/−^ mice. Relative expression of **(H)** LKB1 and **(I)** AMPKα2 based on western blot results (*n* = 6); GAPDH was used for normalization. Data are presented as the mean ± SEM. One-way ANOVA was performed to examine statistical differences among different groups. *p < 0.05 vs. Sham + Veh; #*p* < 0.05 vs. AB + Veh.

To clarify whether LQ treatment attenuates cardiac hypertrophy and improves cardiac function by activating AMPKα2 signaling, AB was performed in AMPKα2 knockout mice (AMPKα2^−/−^). Confirming a central role of AMPKα2 in LQ-mediated cardioprotection, echocardiography demonstrated that the beneficial effects of LQ on HR, LVESd, LVEDd, LVEF, and LVFS observed in WT mice were abrogated in AMPKα2^−/−^ mice (Figures 6A–F). Western blot analysis confirmed efficient AMPKα2 silencing and demonstrated that LQ treatment still promoted cardiac LKB1 phosphorylation in AMPKα2^−/−^ mice (Figures 6G–I). However, no significant differences in cardiac CSA, as well as in HW/BW, LW/BW, and HW/TL values, were noted between LQ- and Veh-treated AMPKα2^−/−^mice (Supplementary Figures S2A–E). Moreover, the expression of hypertrophy-associated genes, including ANP, BNP, and β-MHC, was not significantly different between these two groups (Supplementary Figures S2F–H). Likewise, LQ treatment failed to significantly attenuate cardiac fibrosis and to prevent cardiac expression of collagen I and CTGF in AMPKα2^−/−^ mice (Supplementary Figures S2I–L). These data demonstrated that the beneficial effects of LQ on cardiac hypertrophy and fibrosis were dependent on AMPKα2.

### Liquiritin Inhibits Ang II-Induced Cardiomyocyte Hypertrophy *in Vitro*

**FIGURE 7:**
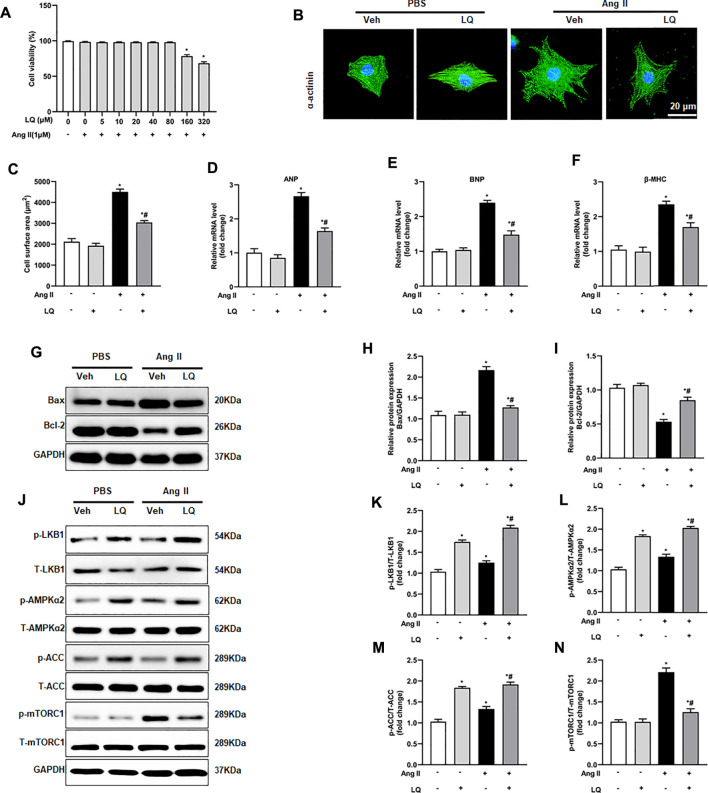
Liquiritin attenuates Ang II-induced cardiomyocyte hypertrophy *in vitro.* **(A)** Analysis of cell viability (CCK-8 assay) in neonatal rat ventricular cardiomyocytes (NRCMs) exposed to different concentrations of LQ (0, 5, 10, 20, 40, 80, 160, and 320 µM) and 1 mM Ang II (*n* = 6). **(B)** Representative images of a-actinin immunofluorescence (green) in NRCMs. Nuclei were stained with DAPI (blue). **(C)** Cell surface area (CSA) estimations based on quantification of a-actinin immunofluorescence (*n* = 6 replicates per group). Relative mRNA expression levels of **(D)** ANP, **(E)** BNP, and **(F)** β-MHC in NRCMs (*n* = 6 per group). **(G)** Representative immunoblots for Bax and Bcl-2. Relative expression levels of **(H)** Bax and **(I)** Bcl-2 based on western blot results (*n* = 6). Signals were normalized to GAPDH. **(J)** Representative immunoblots for p-LKB1, T-LKB1, p-AMPKα2, T-AMPKα2, p-ACC, T-ACC, p-mTORC1, and T-mTORC1, and **(K–N)** corresponding quantification data (*n* = 6). All *in vitro* experiments were repeated three times independently. Data are presented as the mean ± SEM. One-way ANOVA was performed to examine statistical differences among different groups. *p < 0.05 vs. the corresponding PBS + Veh group; #*p* < 0.05 vs. Ang II + Veh.

**FIGURE 8:**
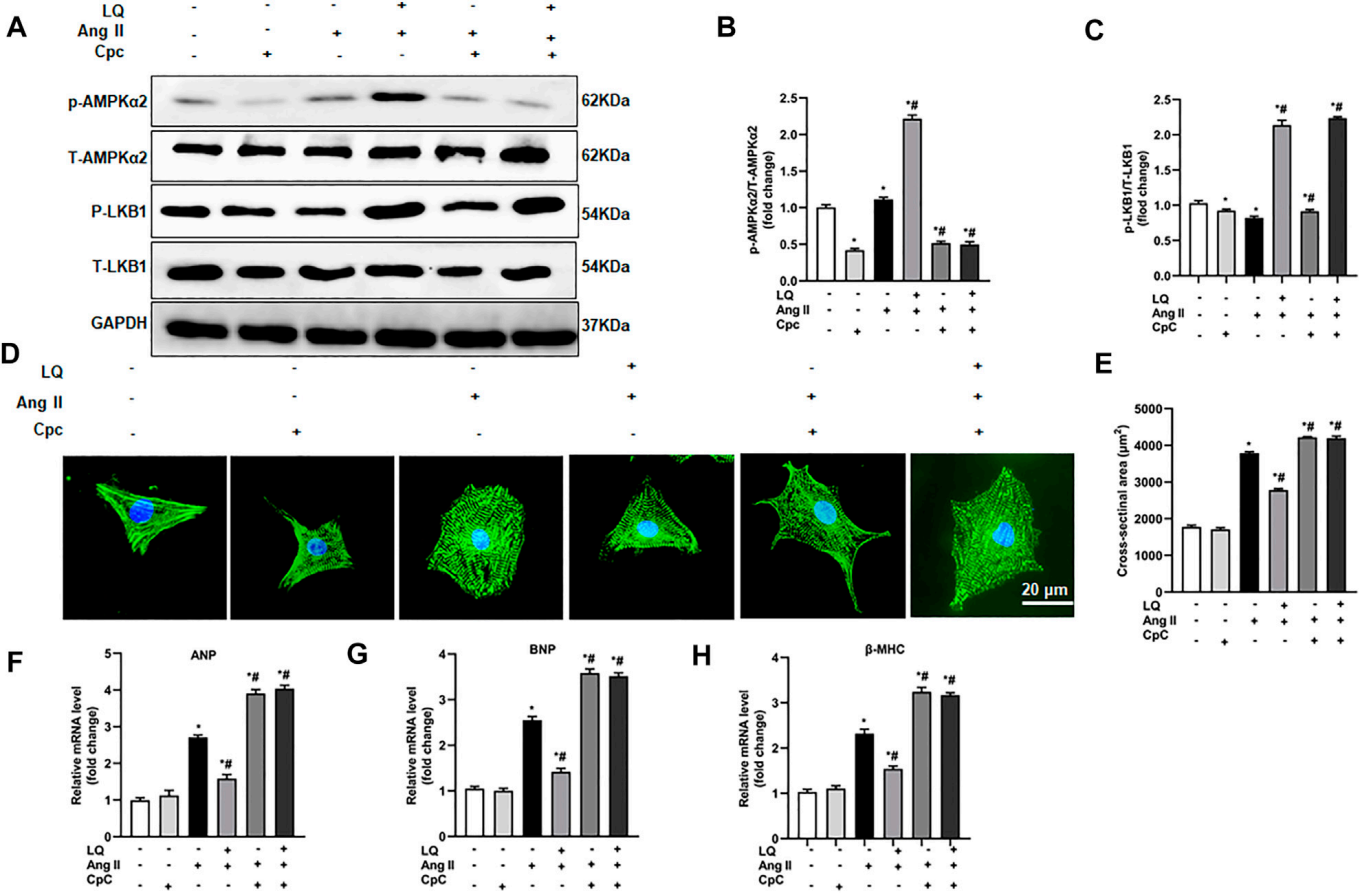
AMPK inhibition abolishes liquiritin’s protective effect against Ang II-induced cardiomyocyte hypertrophy. **(A)** Representative immunoblots for p-AMPKα2, T-AMPKα2, p-LB1, and T-LKB1. Relative expression levels of **(B)** p-AMPKα2/T-AMPKα2 and **(C)** p-LKB1/T-LKB1 based on western blot results (*n* = 6). **(D)** Representative images of α-actinin immunofluorescence (green) in NRCMs. Nuclei were stained with DAPI (blue). **(E)** CSA estimations based on quantification of a-actinin immunofluorescence in NRCMs (*n* = 6 replicates per group). Relative mRNA expression levels of **(F)** ANP, **(G)** BNP, and **(H)** β-MHC in NRCMs (*n* = 6). All *in vitro* experiments were repeated three times independently. Data are presented as the mean ± SEM. One-way ANOVA was performed to examine statistical differences among different groups. *p < 0.05 vs. the corresponding PBS + Veh group; #*p* < 0.05 vs. Ang II + Veh.

To further verify the above findings, an *in vitro* model of cardiomyocyte hypertrophy was established by exposing NRCMs to Ang II. Optimal *in vitro* dosis of LQ was determined through CCK-8 cytotoxicity assays in NRCMs exposed to different concentrations of LQ (0, 5, 10, 20, 40, 80, 160, and 320 μM) and Ang II (1 mM) for 48 h. Since no cytotoxicity was observed with LQ doses ≤80 μM (Figure 7A), in subsequent experiments NRCMs were pre-incubated with 80 μM LQ for 2 h before Ang II exposure. Analysis of NRCM surface area through α-actinin immunofluorescence showed that Ang II treatment (1 mM; 48 h) induced marked NRCM hypertrophy, which could be blocked by pretreatment with LQ (Figures 7B,C). In addition, Ang II treatment markedly increased the transcription of ANP, BNP, and β-MHC, and these changes were significantly inhibited by LQ (Figures 8D–F). Moreover, consistent with the antiapoptotic effect observed *in vivo*, LQ treatment effectively suppressed Bax upregulation and restored Bcl-2 expression in Ang II-treated NRCMs (Figures 7G–I). In line also with its *in vivo* effects, LQ treatment considerably enhanced the phosphorylation of LKB1, AMPKα2, and ACC, and inhibited mTORC1 phosphorylation, in Ang II-treated cells (Figures 7J–N). These data demonstrated that LQ prevents Ang II-induced hypertrophy in NRCMs *in vitro via* regulating the LKB1/AMPKα2/ACC/mTORC1 pathway.

### AMPKα2 Inhibition Abolishes Liquiritin-Mediated Suppression of Ang II-Induced Hypertrophy in Cultured Cardiomyocytes

To confirm the essential role of AMPKα in LQ-mediated inhibition of Ang II-induced hypertrophy in cultured cardiomyocytes, NRCMs were preincubated with compound C (CpC), which reduces AMPKα activity by inhibiting its phosphorylation (Lee et al., 2020). Western blot assays showed that upon co-incubation with CpC, LQ still induced LKB1 phosphorylation but failed to promote AMPKα2 phosphorylation in Ang II-treated NRCMs (Figures 8A–C). Consistently, upon co-incubation with CpC, α-actinin immunofluorescence showed that LQ failed to prevent Ang II-induced hypertrophy (Figures 8D,E). Moreover, when applied along with CpC, LQ no longer inhibited Ang II-induced overexpression of ANP, BNP, and β-MHC in NRCMs (Figures 8F–H). These data confirmed that LQ inhibits Ang II-induced hypertrophy in cultured NRCMs by activating AMPKα2*.*

### Liquiritin Activates AMPKα2 *via* cAMP/PKA Signaling

**FIGURE 9:**
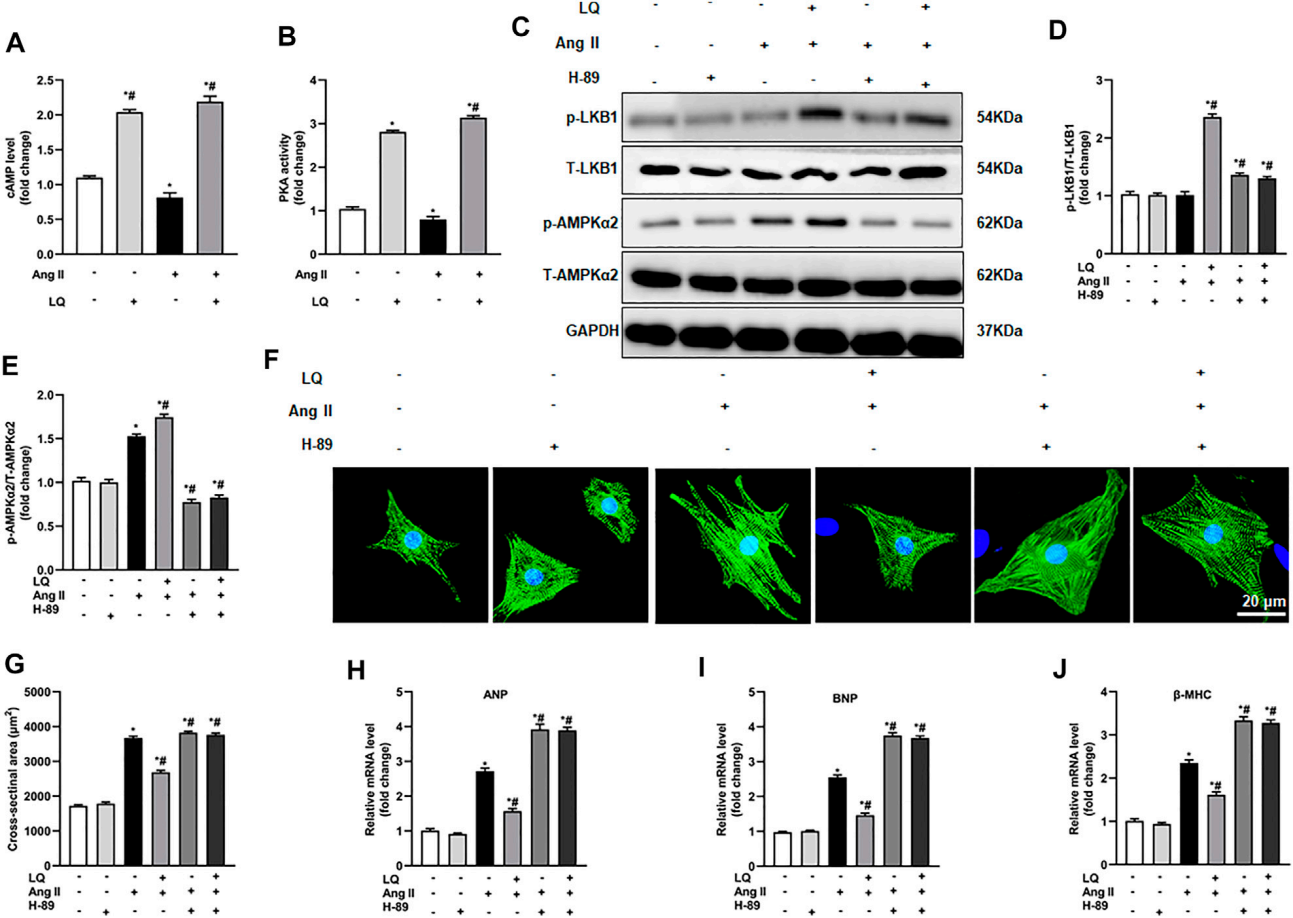
Liquiritin-induced LKB1 phosphorylation is abrogated by PKA inhibition. **(A)** Quantification of cAMP levels in the soluble fraction of NRCM homogenates (*n* = 6). **(B)** Quantification of PKA activity in the soluble fraction of NRCM homogenates (*n* = 6). **(C)** Representative immunoblots for p-LKB1, T-LKB1, p-AMPKα2, and T-AMPKα2. Relative expression levels of **(D)** p-LKB1/T-LKB1 and **(E)** p-AMPKα2/T-AMPKα2 (*n* = 6). **(F)** Representative images of α-actinin immunofluorescence (green) in NRCMs. Nuclei were stained with DAPI (blue). **(G)** CSA estimations based on α-actinin immunofluorescence (*n* = 6 replicates per group). Relative mRNA expression levels of **(H)** ANP, **(I)** BNP, and **(J)** β-MHC (*n* = 6). All *in vitro* experiments were repeated three times independently. Data are presented as the mean ± SEM. One-way ANOVA was performed to examine statistical differences among different groups. *p < 0.05 vs. the corresponding PBS + Veh group; #*p* < 0.05 vs. Ang II + Veh.

Since LQ failed to reduce cardiomyocyte hypertrophy after AMPKα2 knockdown or inhibition but still induced LKB1 phosphorylation, we speculated that LQ might influence AMPKα2 activity indirectly, by targeting upstream regulatory signals. As direct upstream regulation of LKB1/AMPKα2 activation was shown to be effected by signaling through the cyclic adenosine monophosphate (cAMP)/protein kinase A (PKA) pathway (Moberly et al., 2013), and a previous study indicated that LQ activates PKA (Uto et al., 2019), the effect of LQ on cAMP levels and PKA activity were examined in NRCMs. Results showed that Ang II treatment decreased cAMP levels and PKA activity. In contrast, LQ treatment significantly upregulated cAMP levels and increased PKA activity under both control conditions and after Ang II treatment (Figures 9A,B). Of note, co-treatment with the highly selective PKA inhibitor H-89 completely inhibited LQ-mediated LKB1 and AMPKα2 phosphorylation (Figures 9C–E). Moreover, LQ treatment failed to prevent NRCM hypertrophy and to inhibit hypertrophy marker expression after H-89 co-treatment (Figures 9F–J). These data demonstrated that LQ promotes LKB1/AMPKα2 phosphorylation in a cAMP/PKA-dependent manner.

## Discussion

This study illustrated that LQ protected against pressure overload-induced cardiac hypertrophy *in vivo* and Ang II-induced cardiomyocyte hypertrophy *in vitro via* activation of an AMPKα2-dependent pathway. Although AMPKα2 knockdown or inhibition abrogated the beneficial effects of LQ on cardiomyocyte hypertrophy, LQ was still able to activate LKB1, a major upstream activator of AMPKα2, through phosphorylation. Since LQ increased cardiomyocyte cAMP levels, and LQ-mediated LKB1 phosphorylation was prevented after PKA inhibition, we propose that LQ attenuates cardiac hypertrophy by enhancing cAMP/PKA/LKB1/AMPKα pathway activation.

Pathological cardiac hypertrophy is initiated in response to pathological stressors such as hypertension, ischemia, or valvular heart disease. Hypertrophic hearts show enlargement of non-proliferative cardiomyocytes to reduce ventricular wall stress and preserve normal heart function (Nakamura and Sadoshima, 2018). However, sustained pressure overload causes contractile dysfunction, evidenced by decreased LVEF, which eventually leads to heart failure (Nakamura and Sadoshima, 2018). Heart failure is a leading cause of morbidity and mortality worldwide. Despite tremendous progress in drug treatment for managing heart failure, 1-year mortality and hospitalization rates of 7.2% and 31.9%, respectively, have been recently estimated for chronic heart failure patients (Ma et al., 2018). It has therefore been suggested that intervening on pathological cardiac hypertrophy might be a valuable strategy for preventing the development and progress of heart failure at an early stage. In this regard, therapeutic modulation of AMPK-dependent signaling is one of the most promising strategies to effectively treat pathological cardiac hypertrophy (Feng et al., 2018).

AMPK is a central sensor of cellular energy and nutritional status and a key regulator of ATP production during both physiological and pathological conditions. In virtually all eukaryotes, AMPK is formed by heterotrimeric complexes composed of a catalytic subunit (α1-2) and two regulatory subunits (β1-2 and γ1-3) (Hardie, 2014). The α1 subunit is widely expressed, while the α2 subunit is prominently expressed in heart tissue (Nagata and Hirata, 2010). The heart has a high energy demand, required for constant production of ATP necessary for maintaining cardiac functions (Karwi et al., 2018; Lopaschuk et al., 2021). Studies have demonstrated that in the healthy heart, acetyl-CoA-derived ATP generated from fatty acid oxidation (FAO) provides about 40%–70% of the energy supply (Karwi et al., 2018; Sithara and Drosatos, 2021). However, the expression of FAO enzymes is characteristically downregulated in hypertrophic and failing hearts (Sack et al., 1996), resulting in decreased ATP production. AMPK activation phosphorylates and depresses ACC activity; this suppresses the conversion of acetyl-CoA to malonyl-CoA, a potent inhibitor of carnitine acyltransferase I (CPT-1), the rate-limiting enzyme in FAO (Zordoky et al., 2014). Thus, AMPK activation exerts a protective effect against cardiac hypertrophy by promoting FAO and increasing ATP generation in cardiomyocytes (He et al., 2021). Under stress conditions, AMPK plays a fundamental role in cell survival by regulating the mTOR/p70S6K pathway to promote mRNA transcription, protein synthesis, and cell growth (Feng et al., 2018). In this regard, it was shown that excessive activation of mTOR/p70S6K exacerbates pathological hypertrophy and heart failure in AMPKα2 knockout mice (Zhang et al., 2008). The evidence outlined above provides strong support for our findings and suggests that LQ-mediated AMPK activation might be therapeutically effective to alleviate pathological cardiac hypertrophy.

Several upstream signal transduction molecules, including CAMKK2, Akt, and LKB1, may activate AMPKα through phosphorylation (Yan et al., 2018). CAMKK2 is mainly expressed in the brain and endothelial cells, rather than cardiomyocytes. In turn, our previous study demonstrated that LQ does not significantly induce Akt phosphorylation (Mou et al., 2021). LKB1 is encoded by a tumor suppressor gene extensively expressed in all tissues and regulates various signal transduction pathways involved in cell polarity and motility, protein translation, and energy metabolism (Zheng et al., 2009; McCabe et al., 2010). Several lines of evidence stress the fundamental role of the LKB1/AMPK axis in cardiac function and homeostasis. The basal activity of AMPKα2 was significantly decreased in mice with skeletal muscle- and cardiac-specific LKB1 deficiency, and neither ischemia nor anoxia induced AMPKα2 and ACC2 phosphorylation in their hearts (Sakamoto et al., 2006). Cardiac-specific LKB1 deletion caused left ventricular hypertrophy in mice, an effect accompanied by reduced AMPK phosphorylation and increased mTORC1 phosphorylation (Ikeda et al., 2009). Contrarily, the expression of an active LKB1 complex effectively inhibited phenylephrine-induced protein synthesis and hypertrophy in NRCMs (Noga et al., 2007). Along these lines, experiments in rodents indicated that treatment with NAD (Pillai et al., 2010) and the RXRα agonist bexarotene (Zhu et al., 2014) protected against cardiac hypertrophy *via* activating LKB1-AMPK signaling. The present study showed that LQ treatment led to increased phosphorylation (activation) of both LKB1 and AMPK in both mouse hearts and NRCMs, which indicated that LKB1 phosphorylation is the upstream event mediating AMPKα2 activation by LQ.

Several signaling pathways regulate LKB1 activity during pathological cardiac remodeling. Ras/MAPK signaling activation was shown to decrease LKB1/AMPK activation, resulting in impaired mitochondrial homeostasis (Dard et al., 2022). Upregulation of the E3 ubiquitin ligase RNF146 increases LKB1 ubiquitination, resulting in decreased LKB1/AMPK phosphorylation (Sheng et al., 2022). The development of cardiac hypertrophy is accompanied by an overproduction of the lipid peroxidation byproduct 4-hydroxy-2-nonenal (4-HNE), which promotes the formation of HNE-LKB1 adducts, resulting in inhibition of LKB1 phosphorylation (Dolinsky et al., 2009). Conversely, the adiponectin paralog C1q/TNF-related protein 9 (CTRP9) was shown to alleviate high fat diet-induced cardiac hypertrophy by promoting LKB1 phosphorylation (Zuo et al., 2020). On the other hand, it has been firmly established that exacerbation of pressure overload-induced cardiac hypertrophy occurs after AMPKα2 knockout (Viollet et al., 2009). Given the central role of AMPKα2 in supporting myocardial function in the hypertrophied or failing heart, it is possible that alterations in upstream signaling pathways governing LKB1 activation may be responsible for the apparently smaller induction of LKB1 phosphorylation by LQ in AMPKα2^−/−^ compared to WT mice.

**FIGURE 10:**
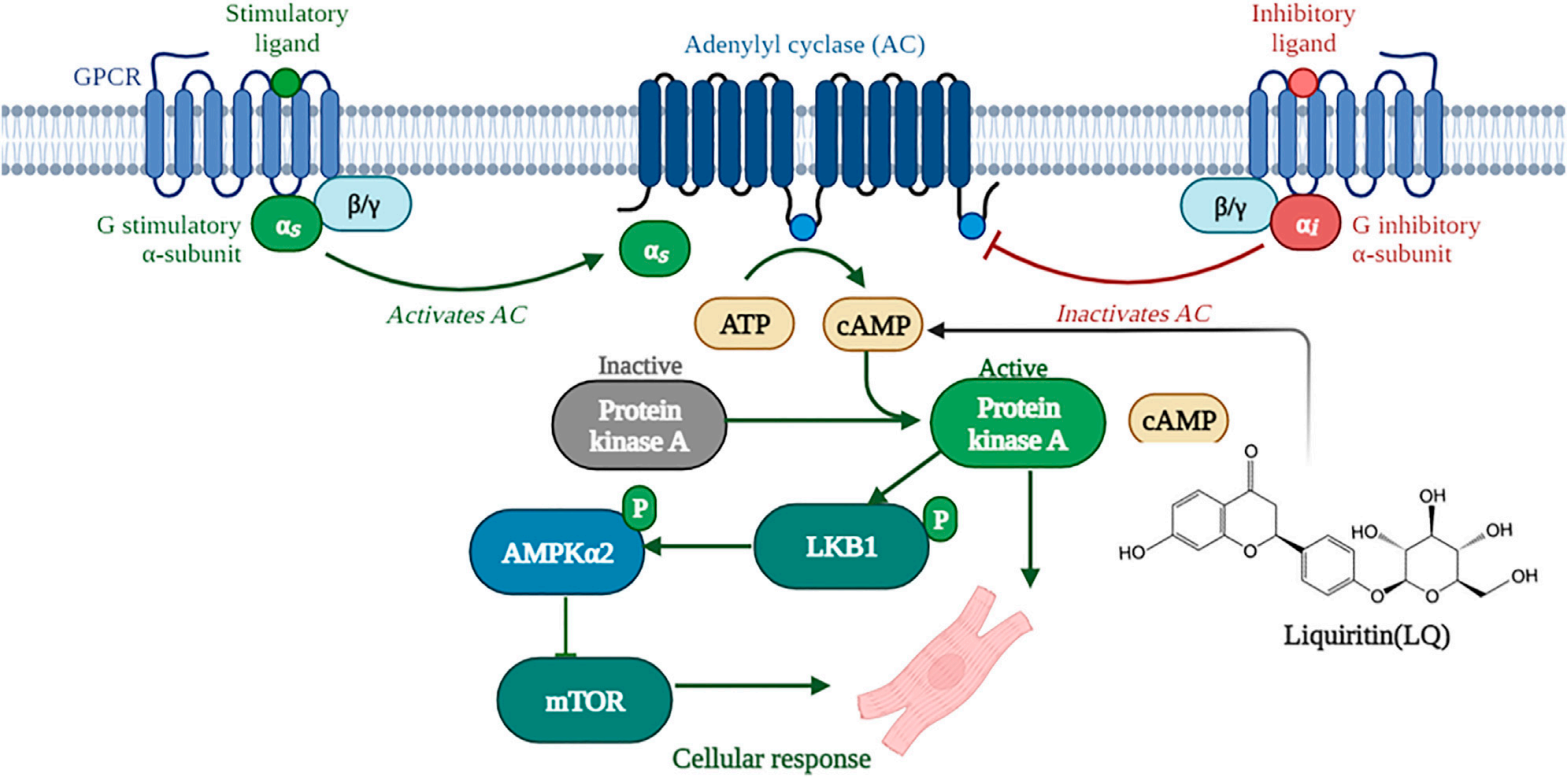
Schematic depiction of the mechanisms underlying liquiritin-mediated protection against cardiac hypertrophy (Created with BioRender.com).

A previous study showed that LQ enhanced melanin synthesis in murine and human melanoma cells lines by promoting cAMP response element-binding protein (CREB) phosphorylation, and this effect was significantly blocked by pretreatment with the PKA inhibitor H-89 (Uto et al., 2019). Similarly, PKA-dependent signaling was shown to underlie LQ-mediated production of nerve growth factor (NGF) and glial cell line-derived neurotrophic factor (GDNF) in rat glial cells (Guo et al., 2020). PKA is the primary effector of cAMP signaling and a direct upstream regulator of LKB1 (Collins et al., 2000). In the heart, PKA becomes activated when β-adrenoceptors on the surface of cardiomyocytes transduce various neurohumoral and mechanical stress signals, resulting in the production of cAMP (Cuello et al., 2021). Activated PKA phosphorylates LKB1 at Ser 431; once activated, LKB1 phosphorylates AMPKα at Thr 172 within its catalytic subunit, leading to AMPK-mediated inhibition of mTORC1 and AAC activity (Huang et al., 2019; Kari et al., 2019). Our study indicated that LQ increased cAMP expression and PKA activity in NRCMs treated with or without Ang II. Moreover, LQ treatment no longer increased LKB1 and AMPKα2 phosphorylation and failed to prevent Ang II-induced hypertrophy in NRCMs after inhibiting PKA with H-89. Based on the evidence discussed above, we conclude that LQ protects against cardiac hypertrophy by activating the cAMP/PKA/LKB1/AMPKα2 signaling pathway (Figure 10).

## Conclusion

Our study revealed that LQ treatment reduced hypertrophy-related cardiac remodeling *in vivo* and prevented Ang II-mediated cardiomyocyte hypertrophy *in vitro*. Mechanistically, our data indicated that LQ increased cAMP levels and activated PKA activity, which subsequently promoted LBK1-mediated AMPKα2 phosphorylation leading to inhibition of mTORC1 and ACC activity. These findings suggest that LQ is a potential drug candidate for therapy or adjuvant therapy of cardiac remodeling.

## Data Availability

The original contributions presented in the study are included in the article/Supplementary Material, further inquiries can be directed to the corresponding author.
