# Supplementary figures and images for: Liquiritin Attenuates Pathological Cardiac Hypertrophy by Activating the PKA/LKB1/AMPK Pathway (part 2 of 2)
Source: Front Pharmacol. 2022 May 3;13:870699. doi: 10.3389/fphar.2022.870699 (PMC9110825; doi:10.3389/fphar.2022.870699)

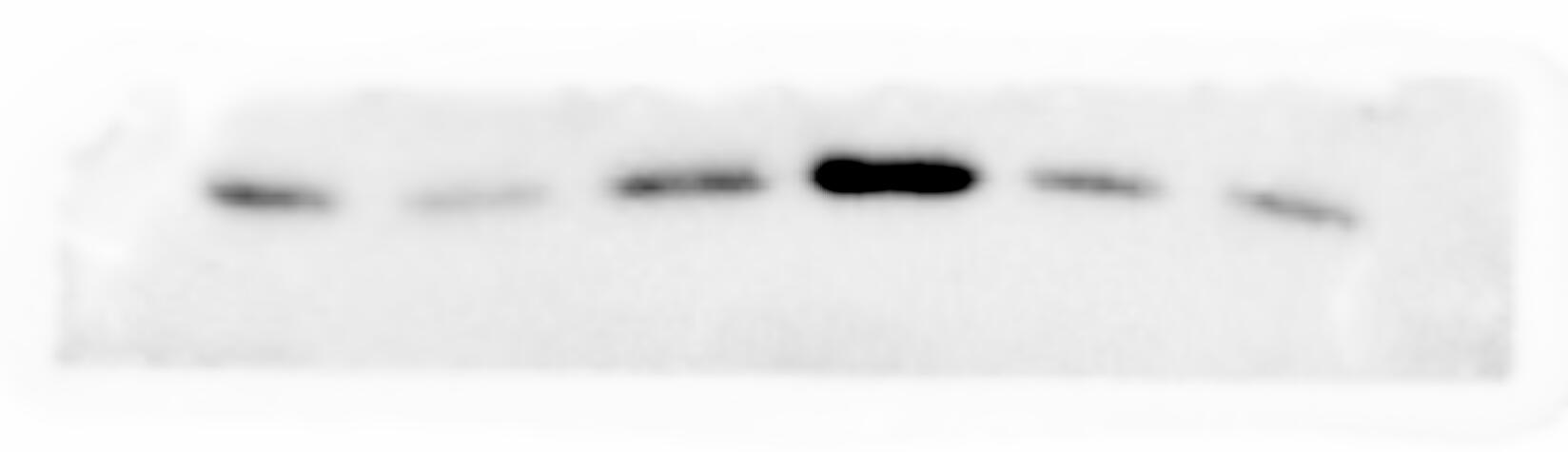

Supplement: Supplementary file 1 [file DataSheet1.ZIP › Additinal files/Western blots/Figure 8.D Western blots/Figure 8.D p-AMPKa2.jpg]

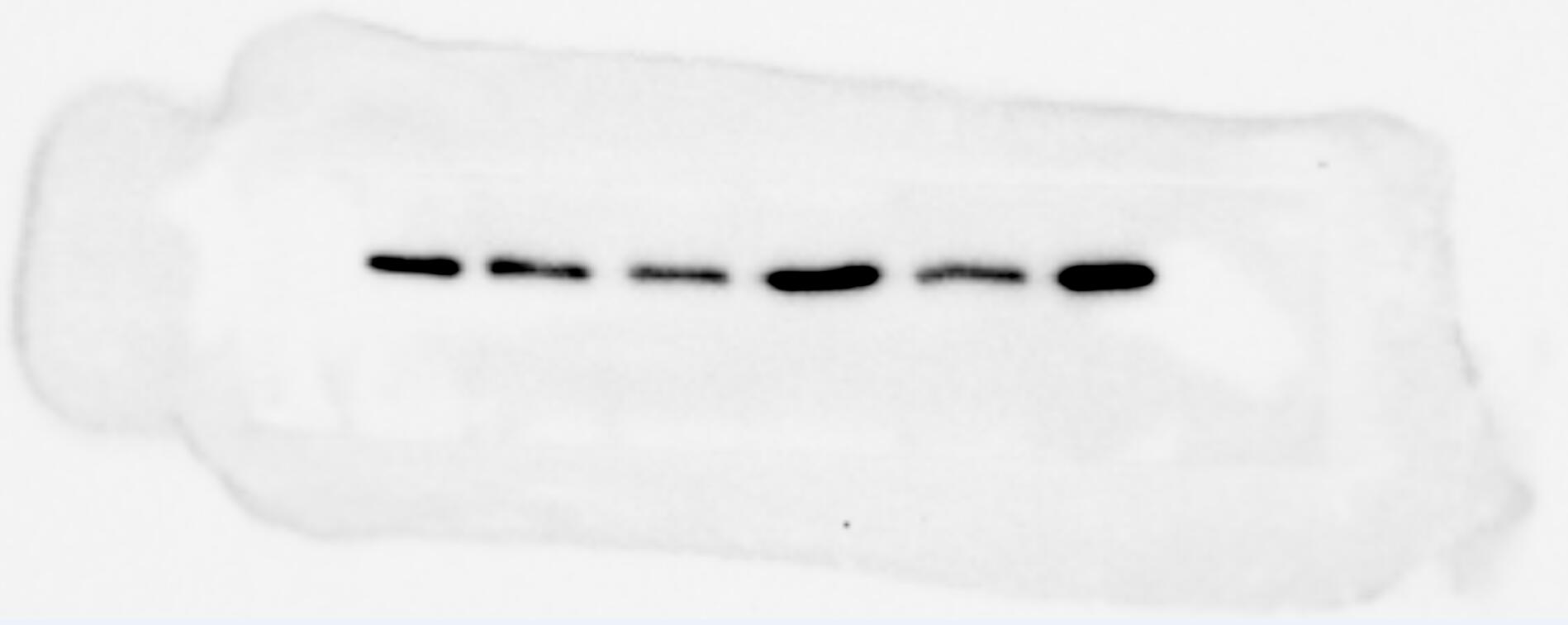

Supplement: Supplementary file 1 [file DataSheet1.ZIP › Additinal files/Western blots/Figure 8.D Western blots/Figure 8.D p-LKB1.jpg]

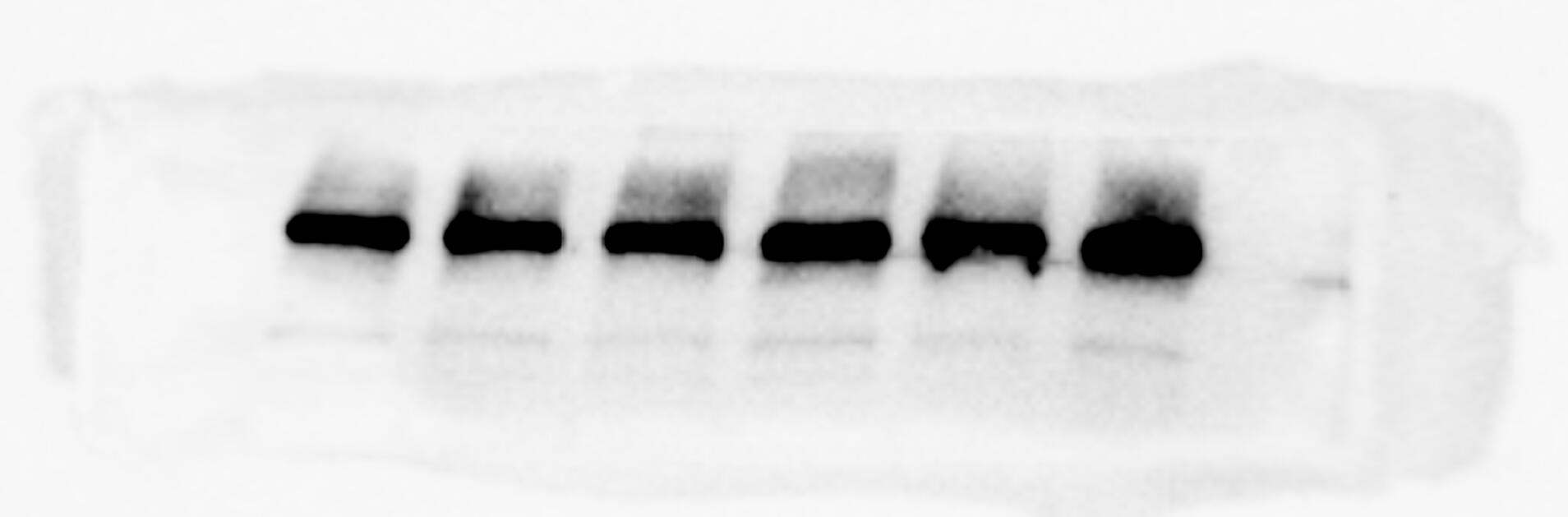

Supplement: Supplementary file 1 [file DataSheet1.ZIP › Additinal files/Western blots/Figure 8.D Western blots/Figure 8.D T-AMPKa2.jpg]

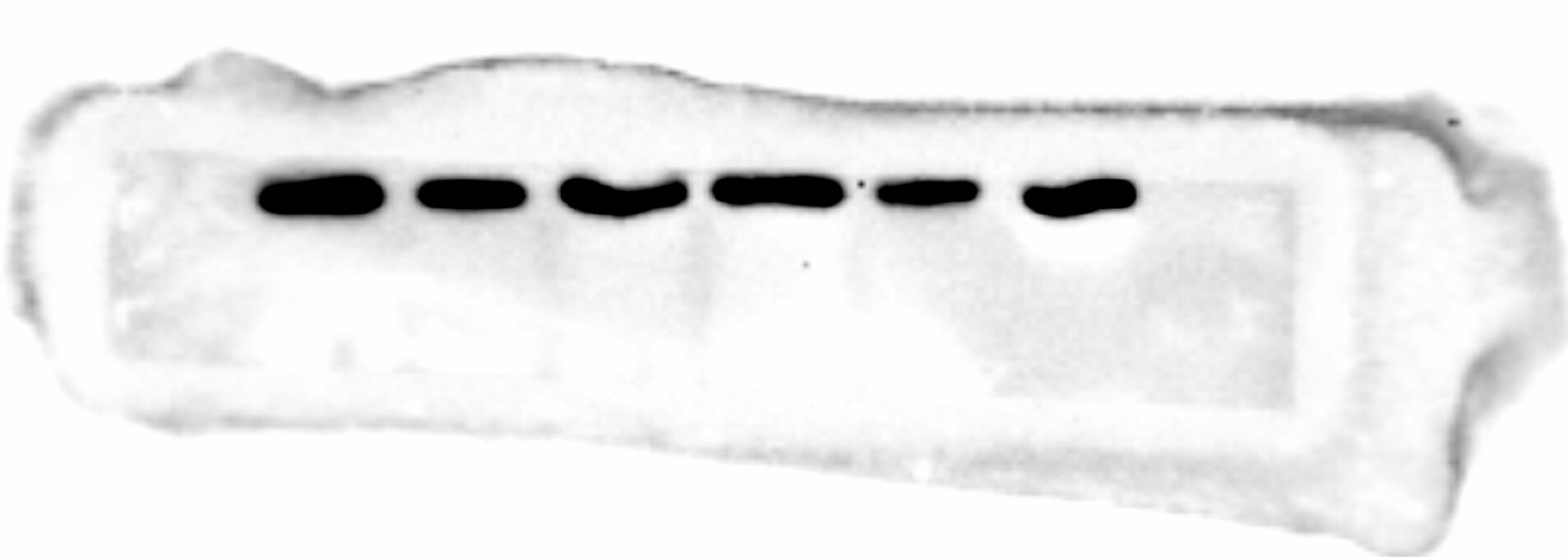

Supplement: Supplementary file 1 [file DataSheet1.ZIP › Additinal files/Western blots/Figure 8.D Western blots/Figure 8.D T-LKB1.jpg]

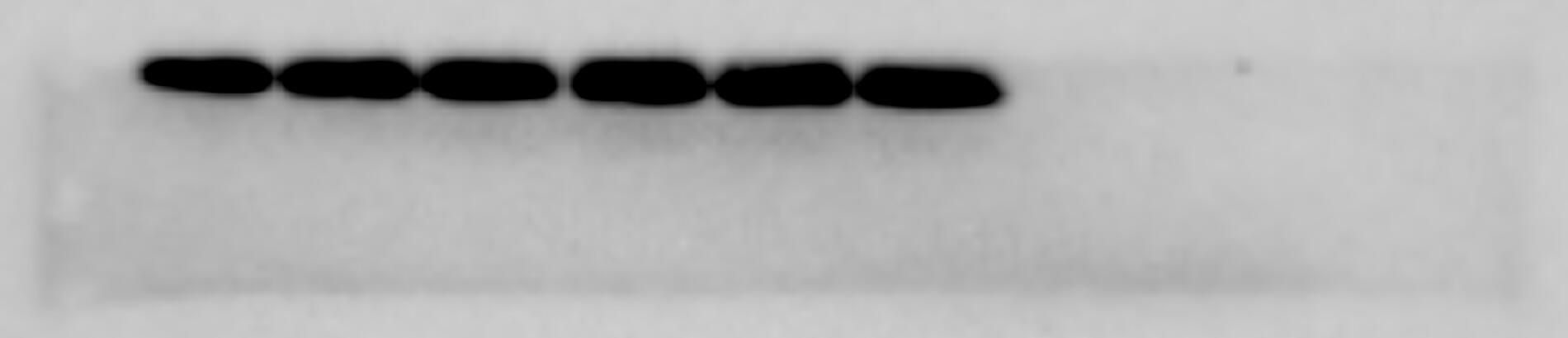

Supplement: Supplementary file 1 [file DataSheet1.ZIP › Additinal files/Western blots/Figure 9.C Western blots/Figure 9.C GAPDH.jpg]

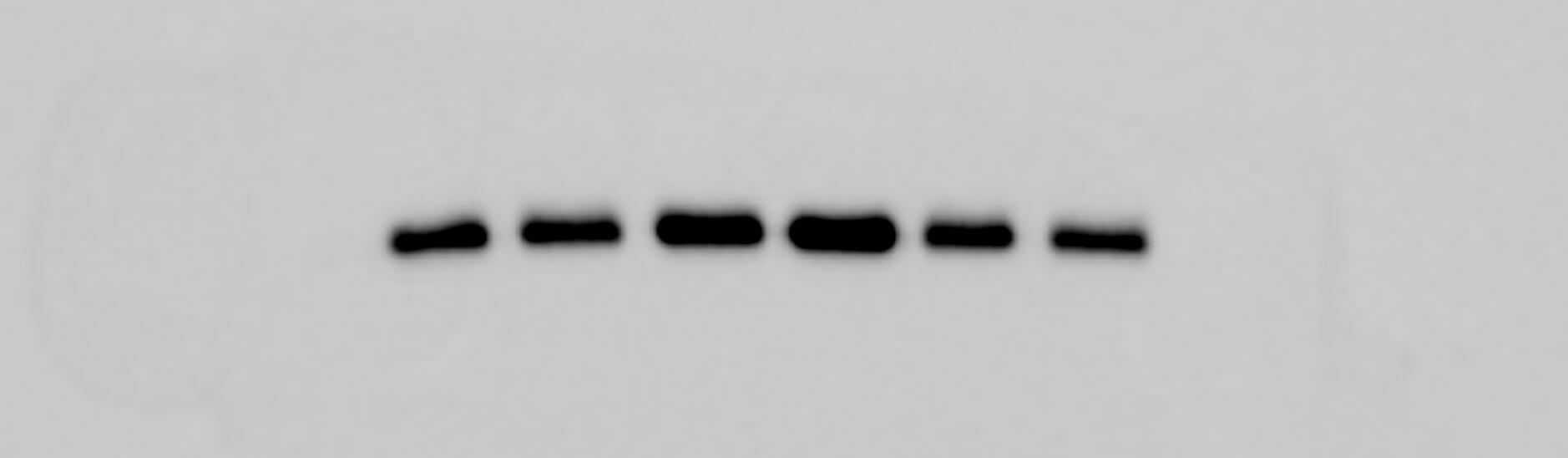

Supplement: Supplementary file 1 [file DataSheet1.ZIP › Additinal files/Western blots/Figure 9.C Western blots/Figure 9.C p-AMPKa2.jpg]

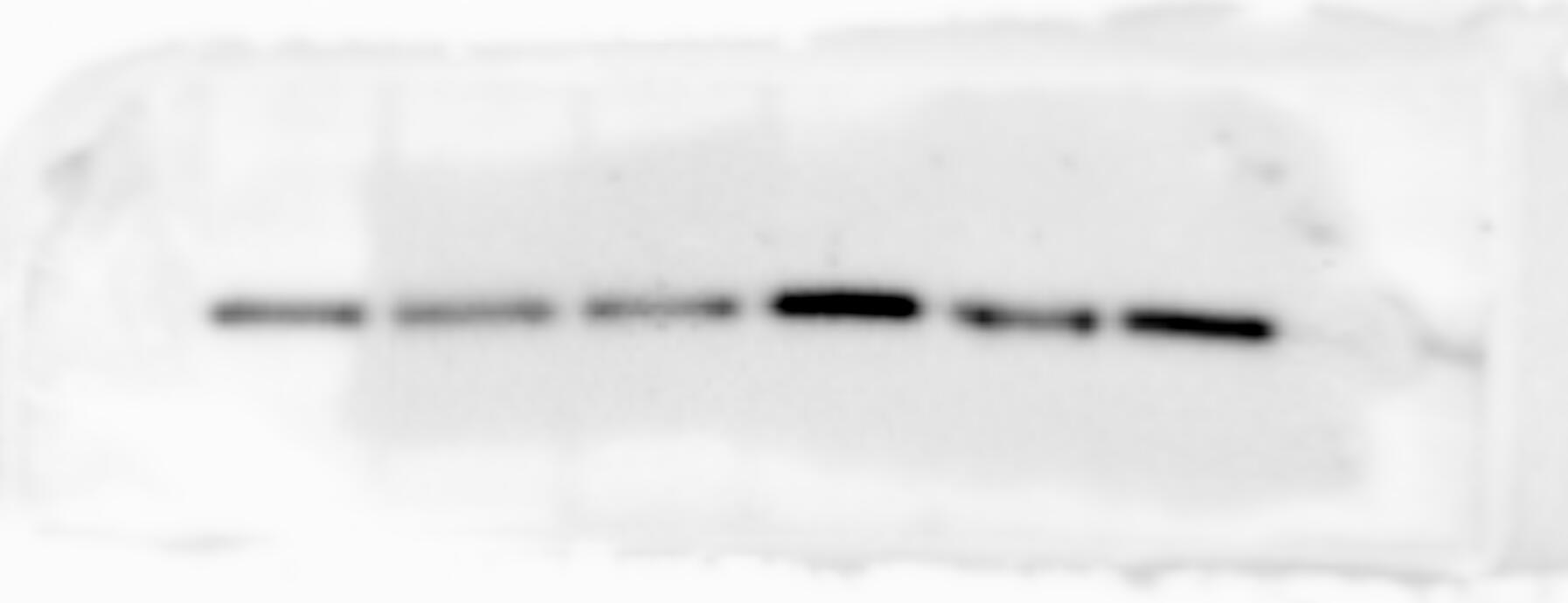

Supplement: Supplementary file 1 [file DataSheet1.ZIP › Additinal files/Western blots/Figure 9.C Western blots/Figure 9.C p-LKB1.jpg]

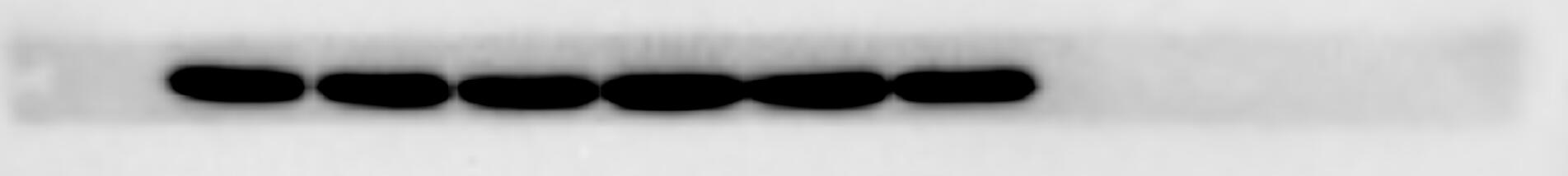

Supplement: Supplementary file 1 [file DataSheet1.ZIP › Additinal files/Western blots/Figure 9.C Western blots/Figure 9.C T-AMPKa2.jpg]

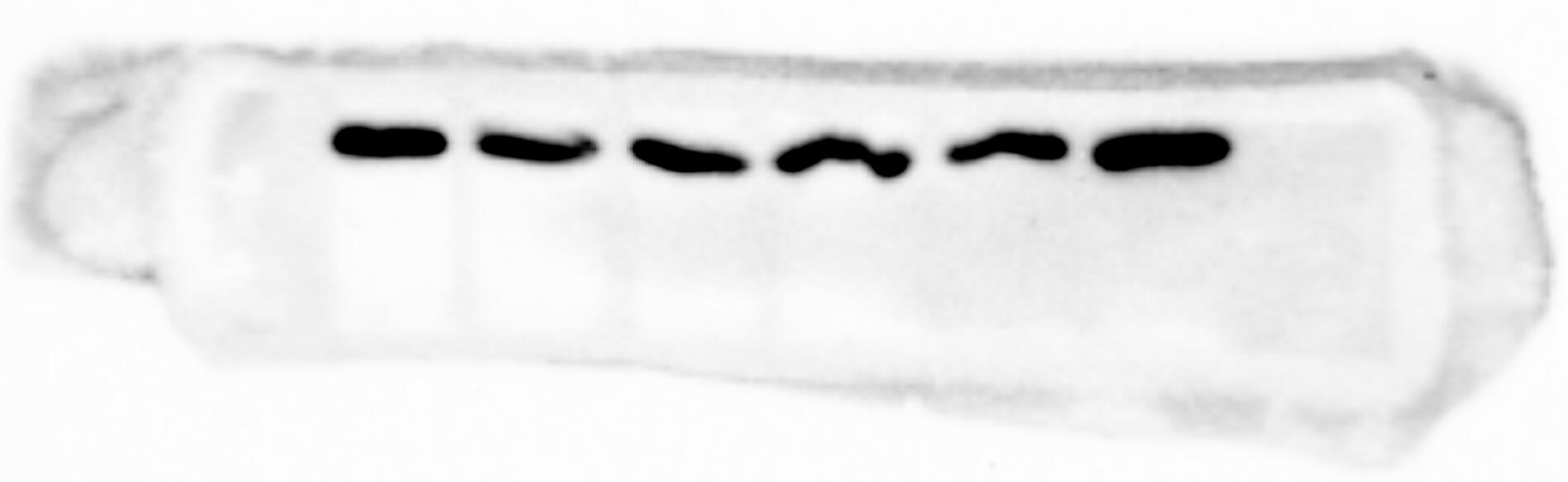

Supplement: Supplementary file 1 [file DataSheet1.ZIP › Additinal files/Western blots/Figure 9.C Western blots/Figure 9.C T-LKB1.jpg]

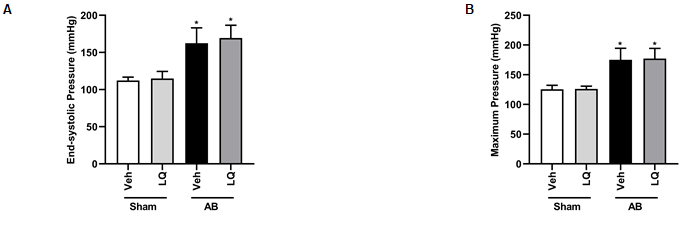

Supplement: Supplementary file 2 [file DataSheet2.ZIP › Supplementary Figures/supplementary Figure S1.tif]

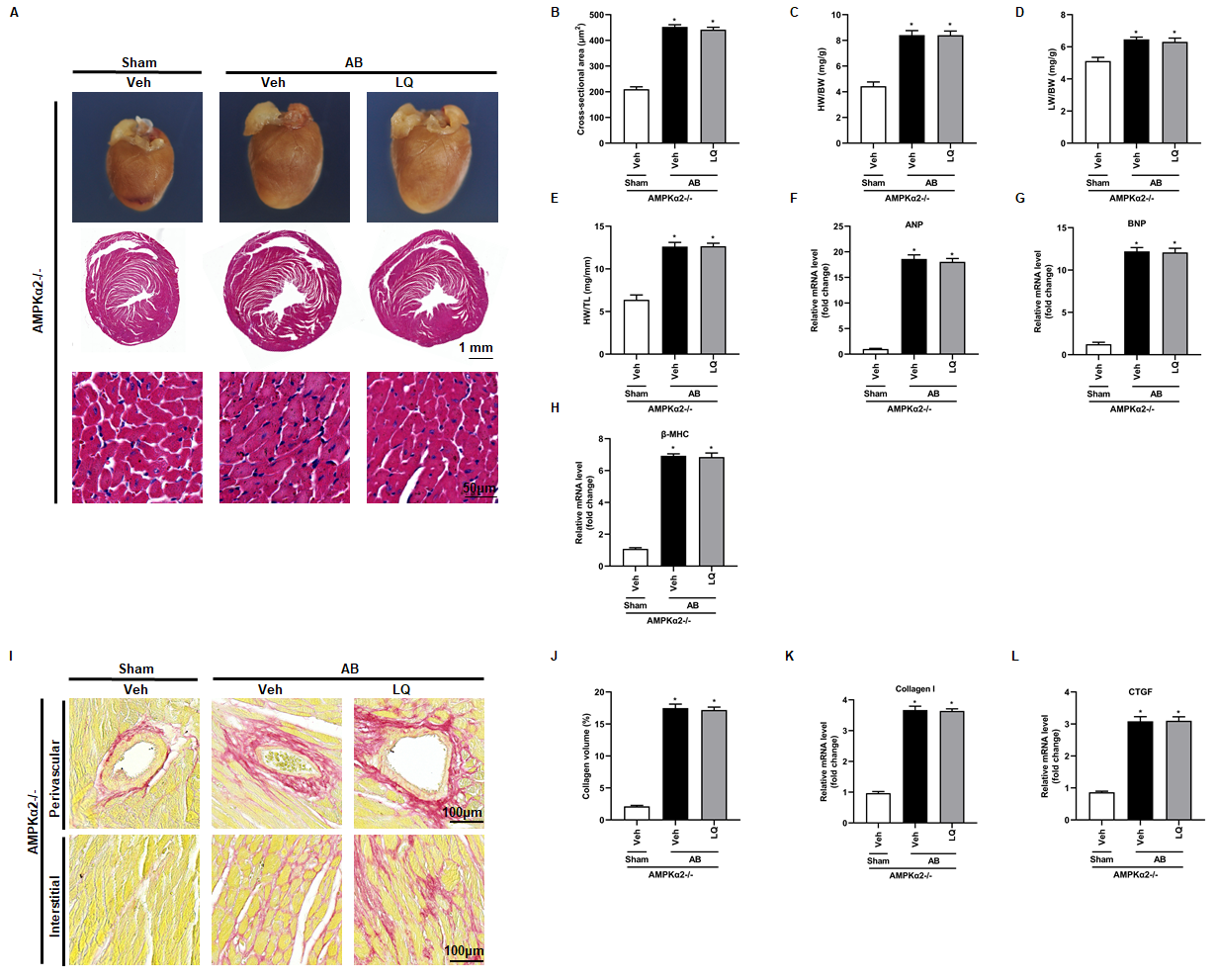

Supplement: Supplementary file 2 [file DataSheet2.ZIP › Supplementary Figures/supplementary Figure S2.tif]
