# Supplementary figures and images for: Liquiritin Attenuates Pathological Cardiac Hypertrophy by Activating the PKA/LKB1/AMPK Pathway (part 1 of 2)
Source: Front Pharmacol. 2022 May 3;13:870699. doi: 10.3389/fphar.2022.870699 (PMC9110825; doi:10.3389/fphar.2022.870699)

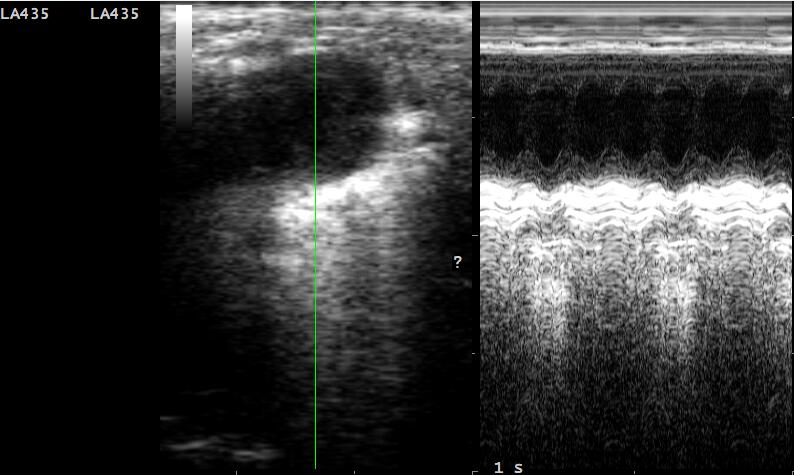

Supplement: Supplementary file 1 [file DataSheet1.ZIP › Additinal files/Echocardiography/Figure 1.A/AB+LQ.jpg]

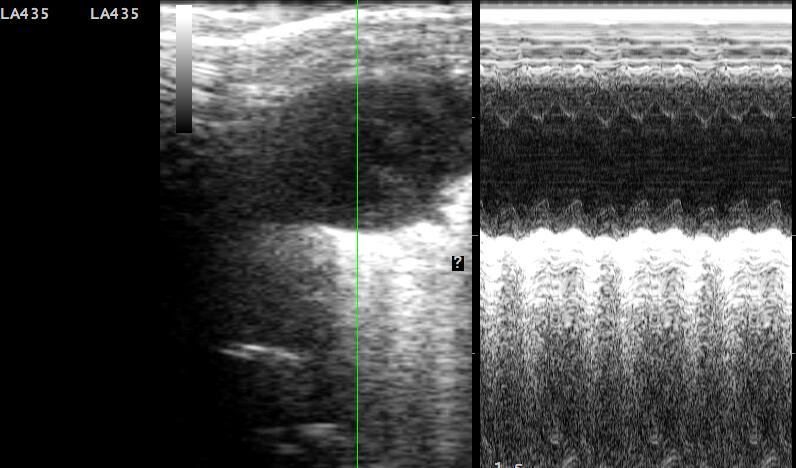

Supplement: Supplementary file 1 [file DataSheet1.ZIP › Additinal files/Echocardiography/Figure 1.A/AB+Veh.jpg]

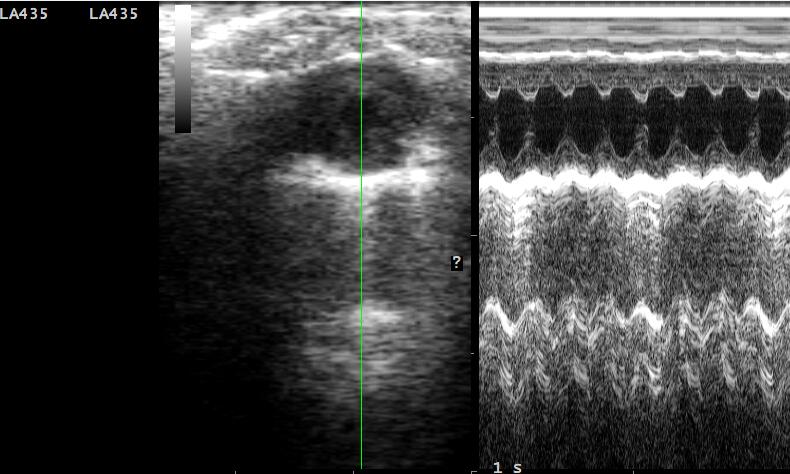

Supplement: Supplementary file 1 [file DataSheet1.ZIP › Additinal files/Echocardiography/Figure 1.A/Sham+LQ.jpg]

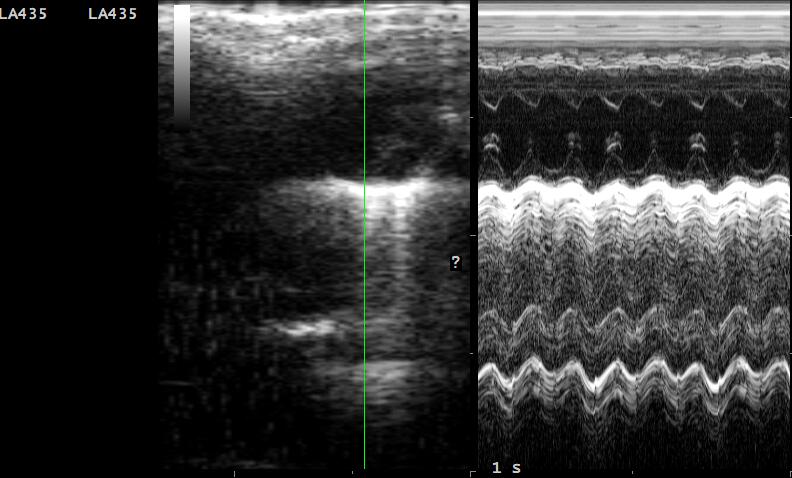

Supplement: Supplementary file 1 [file DataSheet1.ZIP › Additinal files/Echocardiography/Figure 1.A/Sham+Veh.jpg]

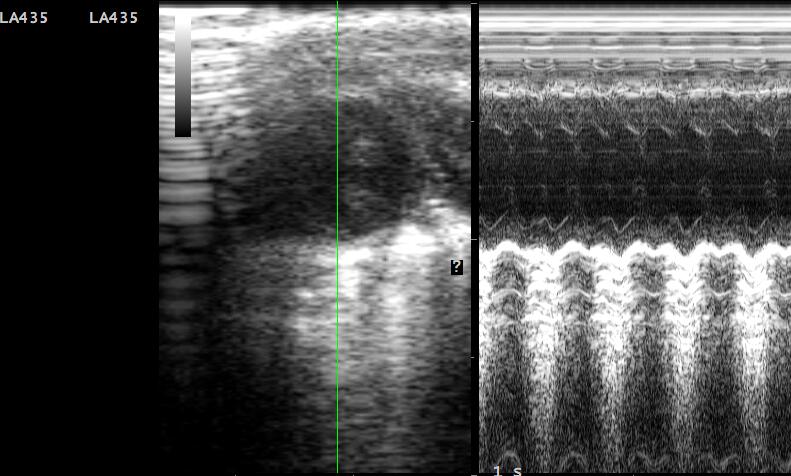

Supplement: Supplementary file 1 [file DataSheet1.ZIP › Additinal files/Echocardiography/Figure 6.A/KO AB+LQ.jpg]

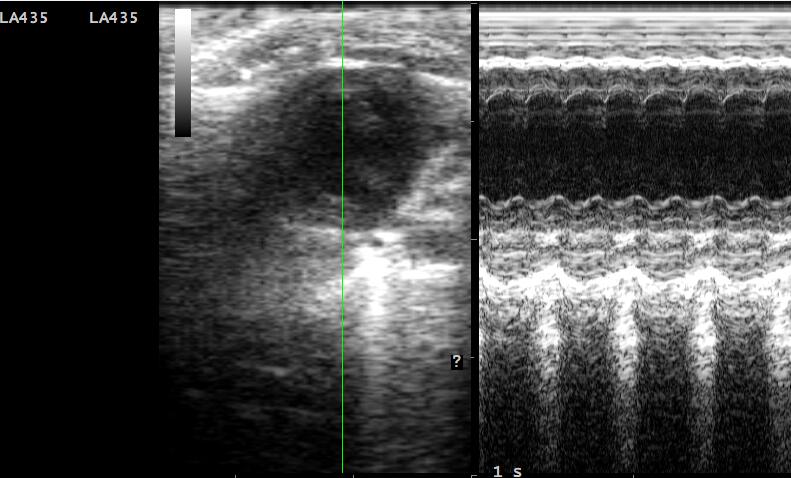

Supplement: Supplementary file 1 [file DataSheet1.ZIP › Additinal files/Echocardiography/Figure 6.A/KO AB+Veh.jpg]

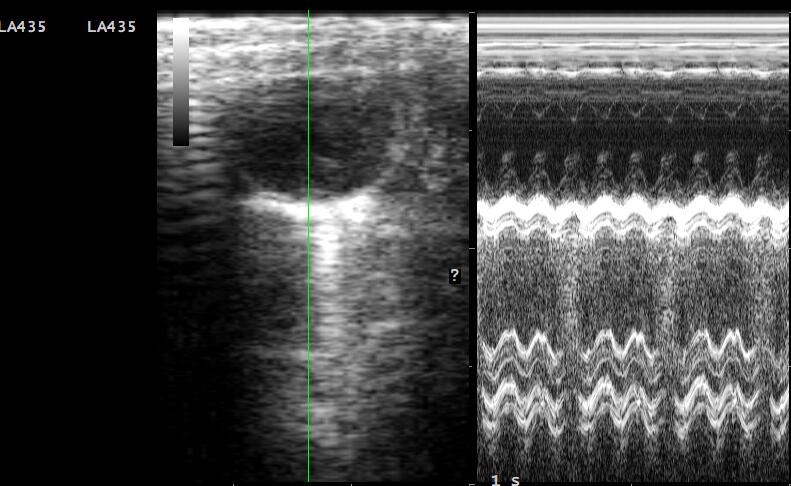

Supplement: Supplementary file 1 [file DataSheet1.ZIP › Additinal files/Echocardiography/Figure 6.A/KO Sham+Veh.jpg]

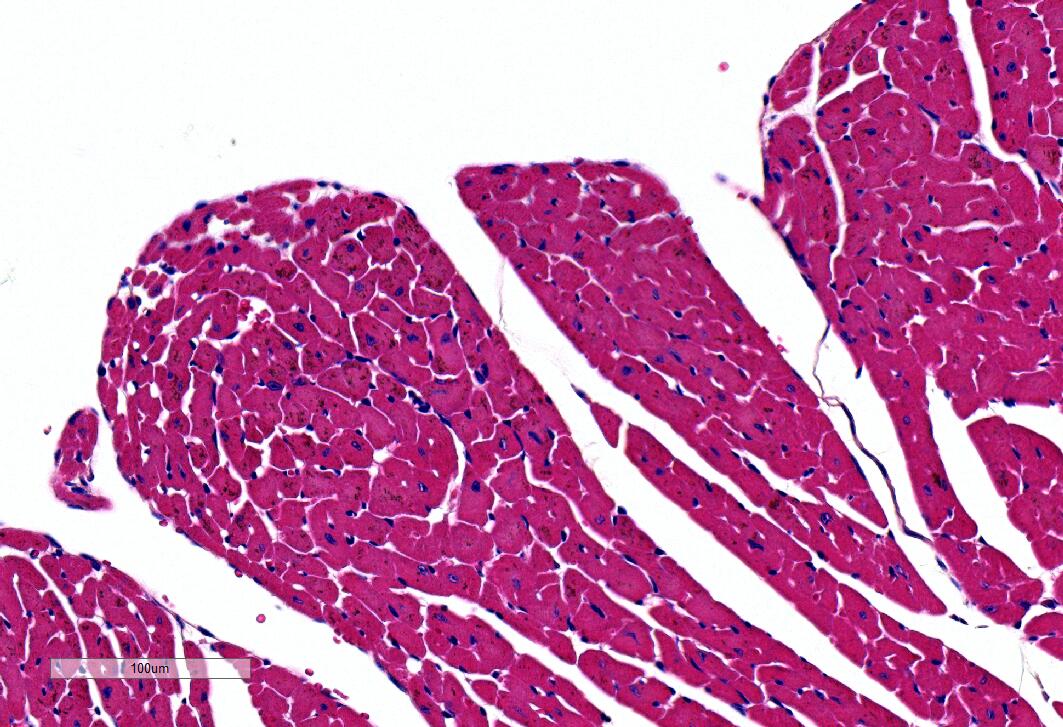

Supplement: Supplementary file 1 [file DataSheet1.ZIP › Additinal files/HE staining/Figure 2.A HE/AB+LQ 200X.jpg]

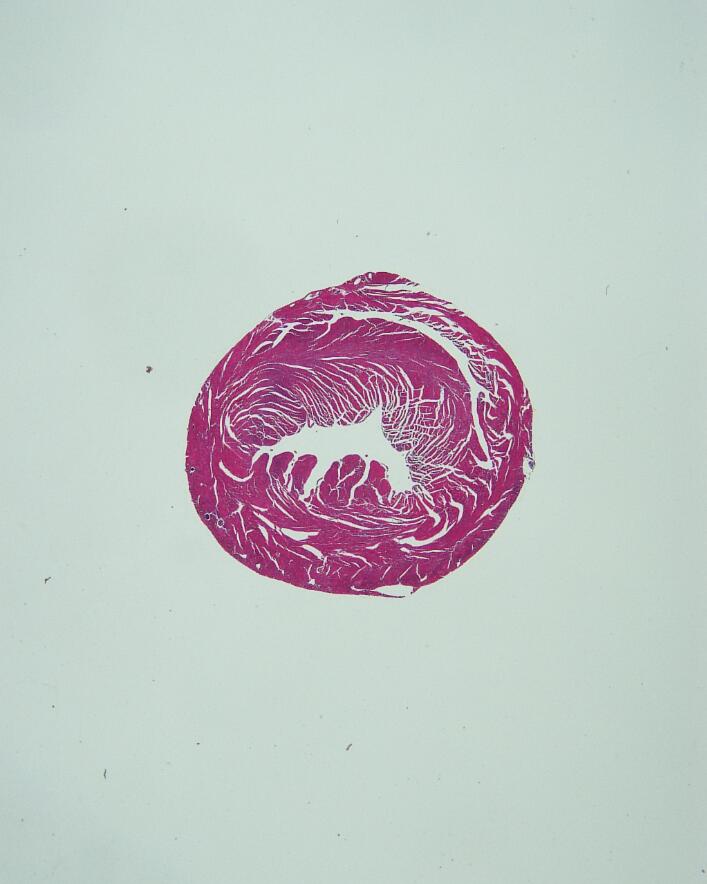

Supplement: Supplementary file 1 [file DataSheet1.ZIP › Additinal files/HE staining/Figure 2.A HE/AB+LQ 40X.jpg]

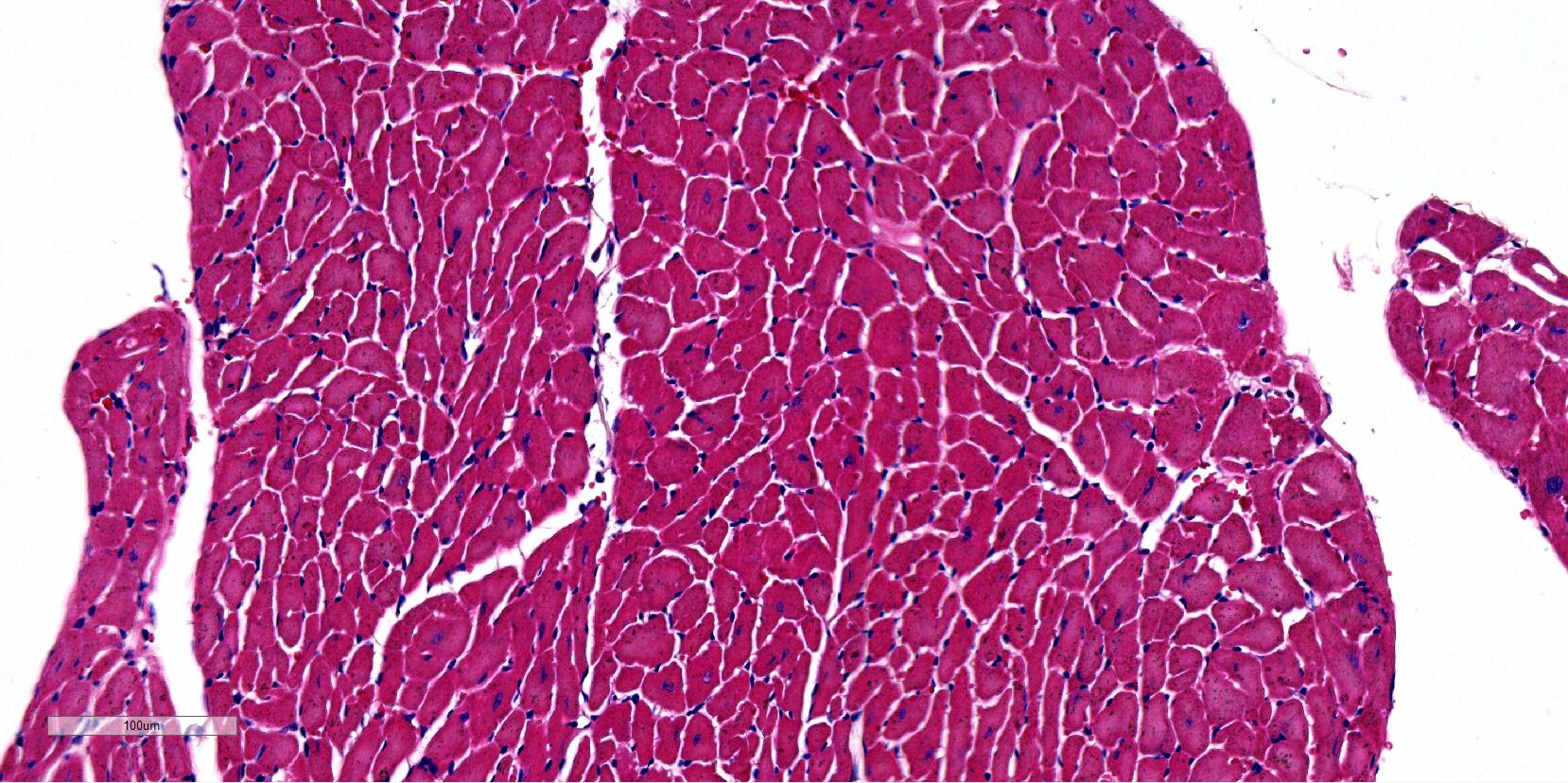

Supplement: Supplementary file 1 [file DataSheet1.ZIP › Additinal files/HE staining/Figure 2.A HE/AB+Veh 200X.jpg]

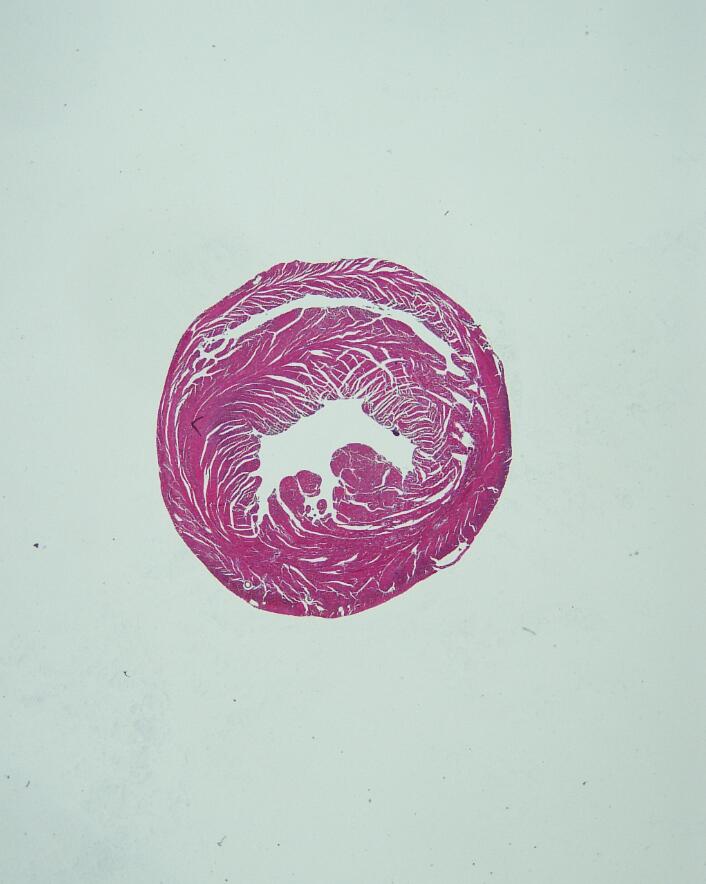

Supplement: Supplementary file 1 [file DataSheet1.ZIP › Additinal files/HE staining/Figure 2.A HE/AB+Veh 40X.jpg]

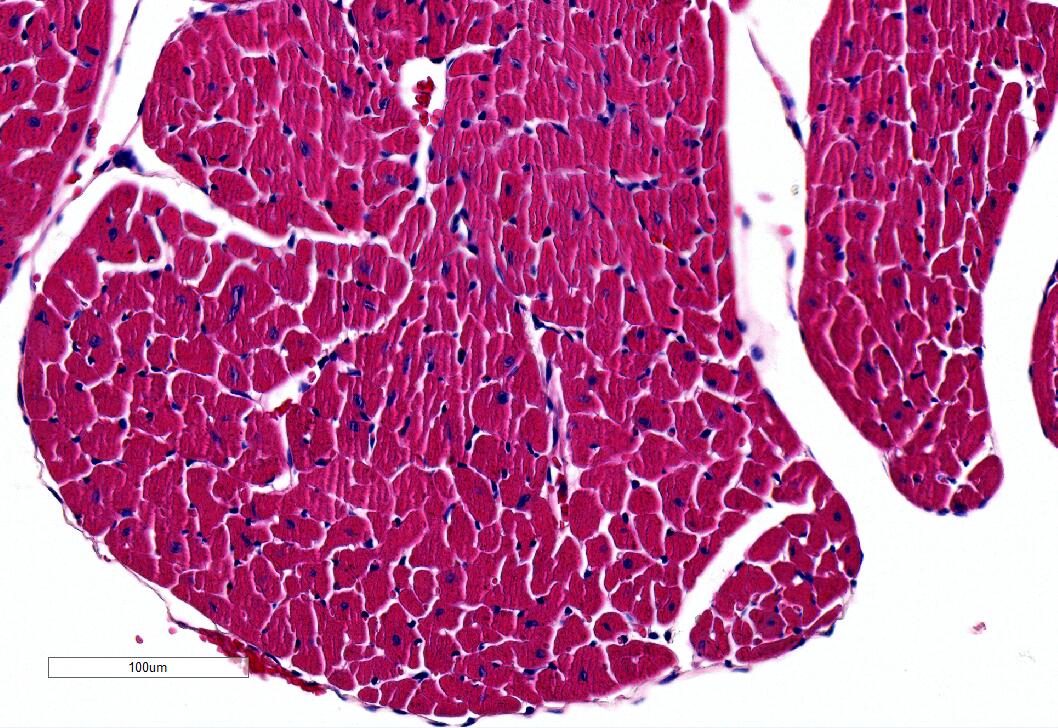

Supplement: Supplementary file 1 [file DataSheet1.ZIP › Additinal files/HE staining/Figure 2.A HE/Sham+LQ 200X.jpg]

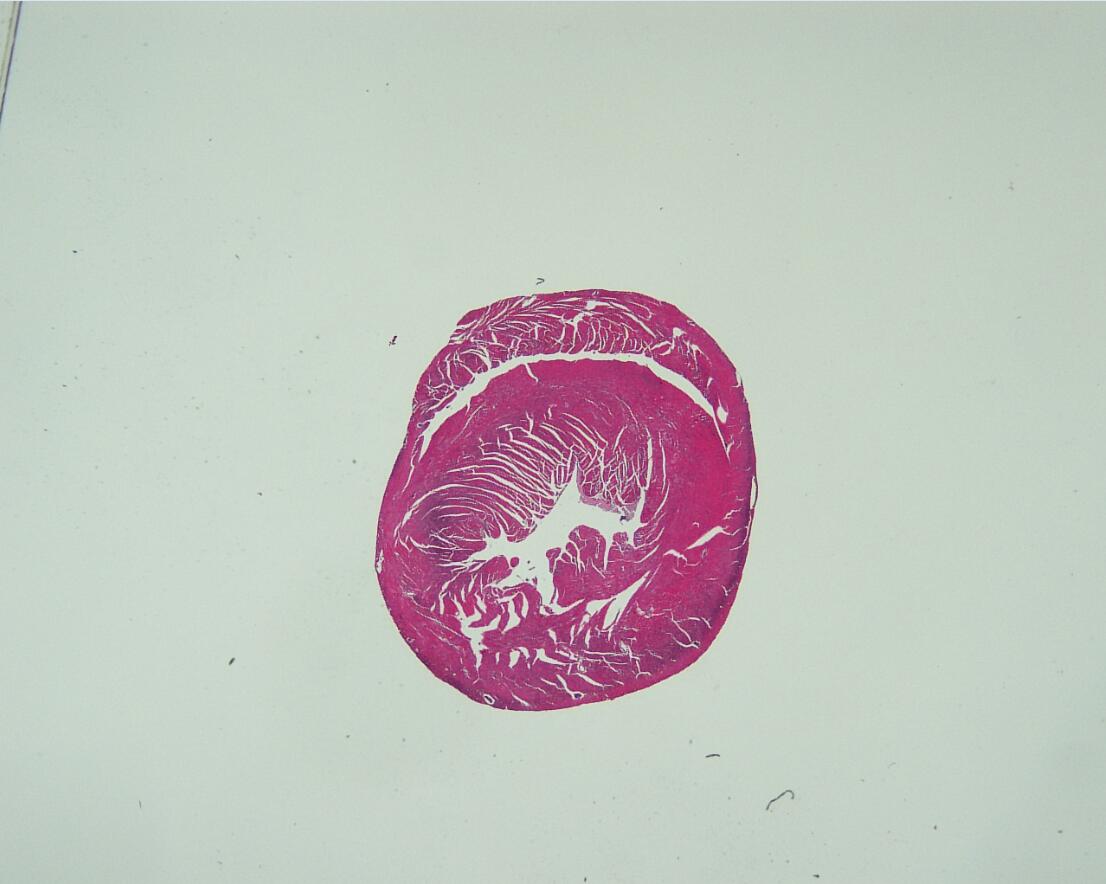

Supplement: Supplementary file 1 [file DataSheet1.ZIP › Additinal files/HE staining/Figure 2.A HE/Sham+LQ 40X.jpg]

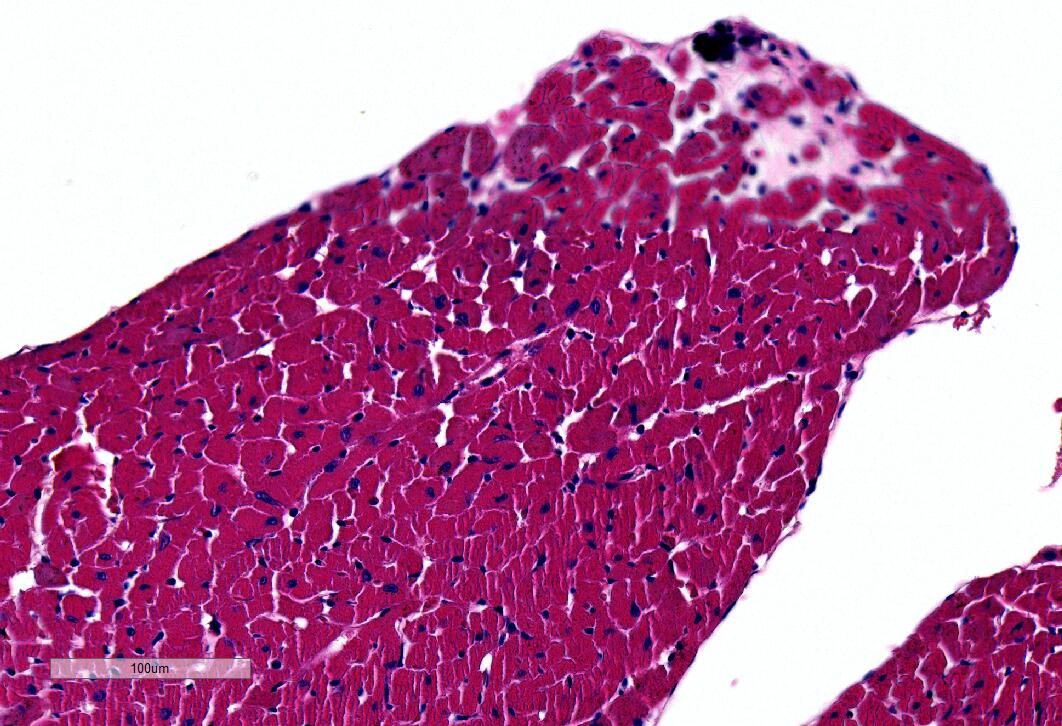

Supplement: Supplementary file 1 [file DataSheet1.ZIP › Additinal files/HE staining/Figure 2.A HE/Sham+Veh 200X.jpg]

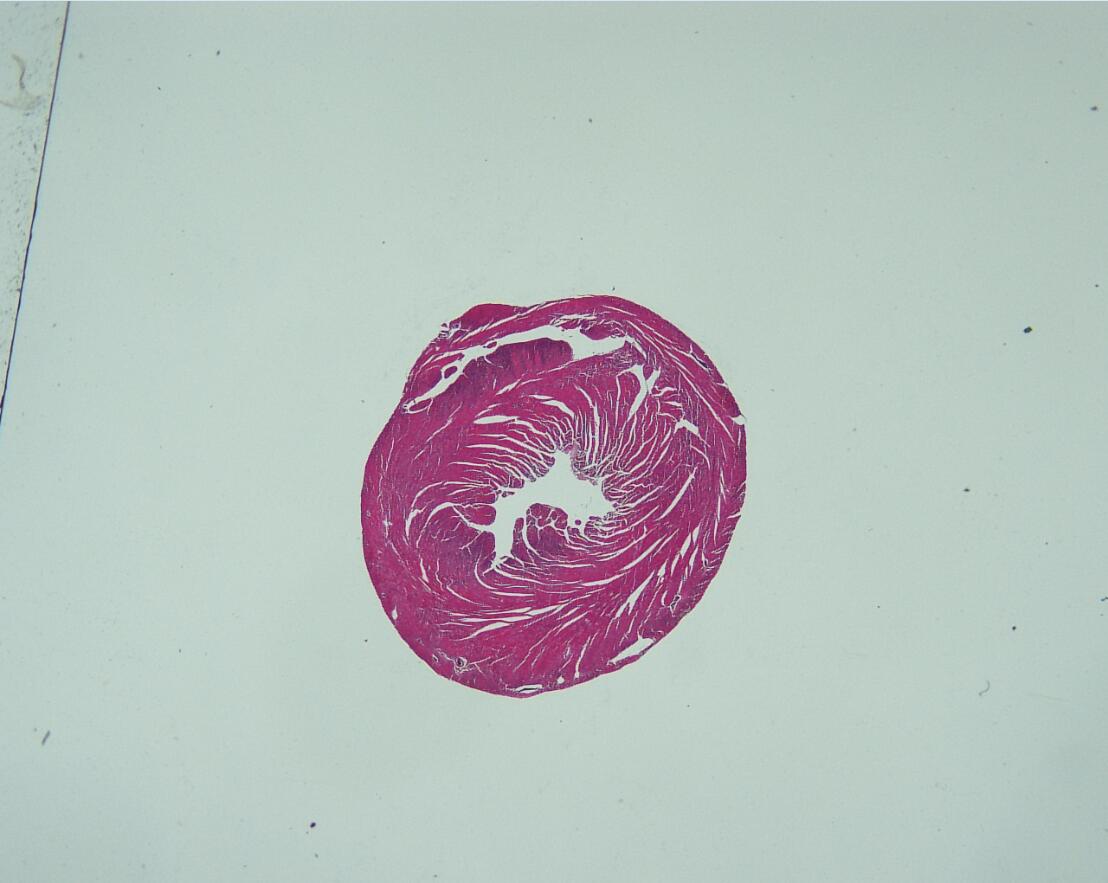

Supplement: Supplementary file 1 [file DataSheet1.ZIP › Additinal files/HE staining/Figure 2.A HE/Sham+Veh 40X.jpg]

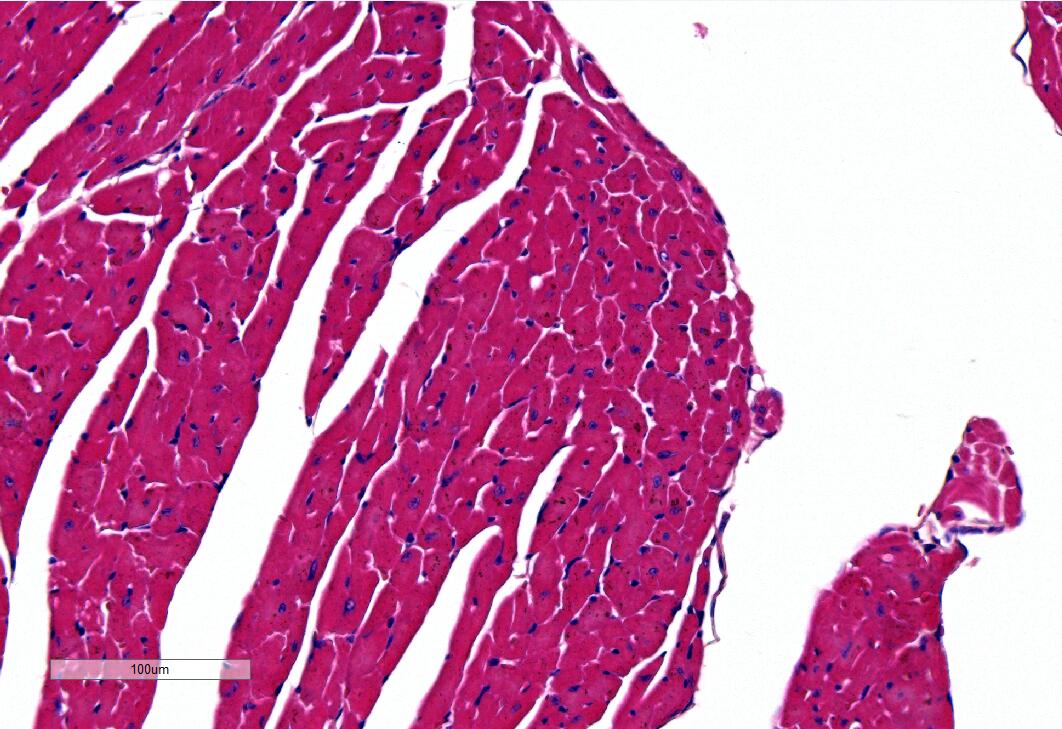

Supplement: Supplementary file 1 [file DataSheet1.ZIP › Additinal files/HE staining/Supplementary Figure S1.A HE/KO AB+LQ 200X.jpg]

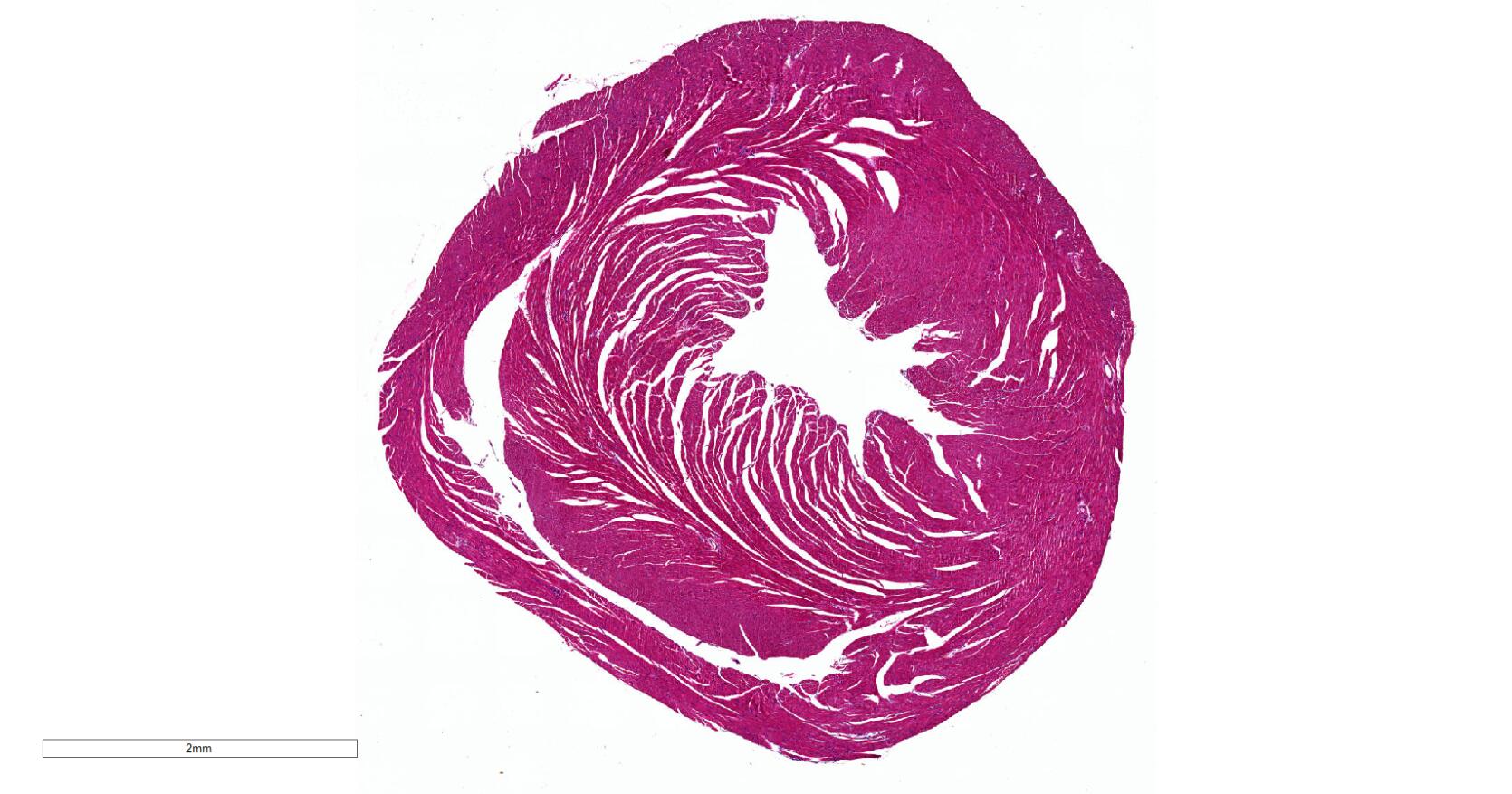

Supplement: Supplementary file 1 [file DataSheet1.ZIP › Additinal files/HE staining/Supplementary Figure S1.A HE/KO AB+LQ 40X.jpg]

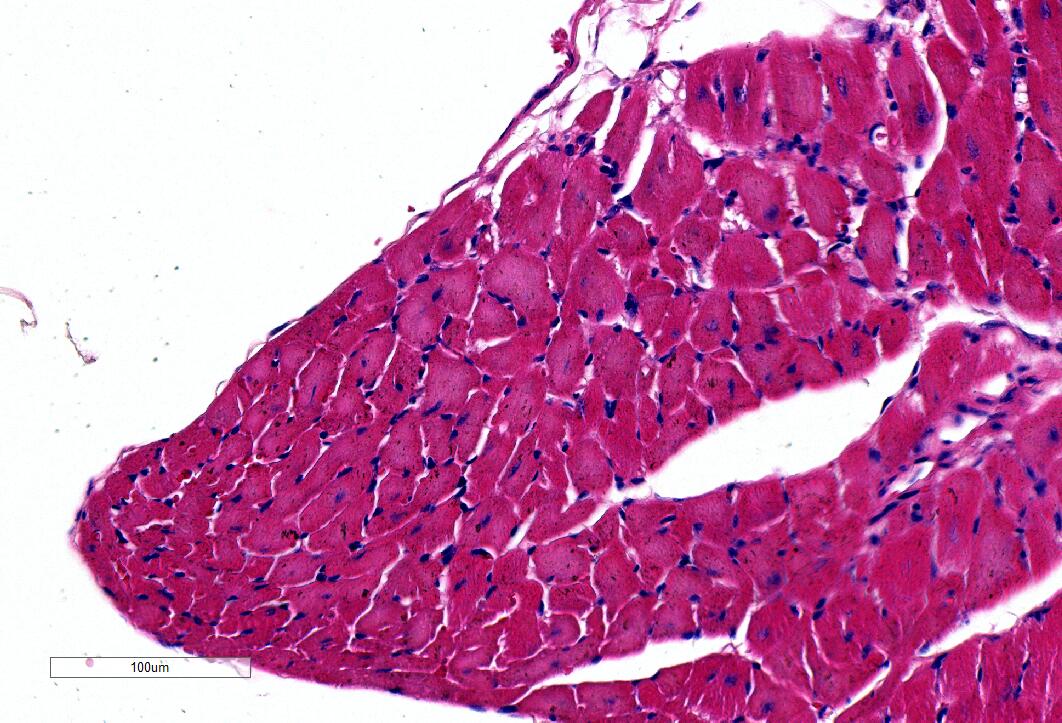

Supplement: Supplementary file 1 [file DataSheet1.ZIP › Additinal files/HE staining/Supplementary Figure S1.A HE/KO AB+Veh 200X.jpg]

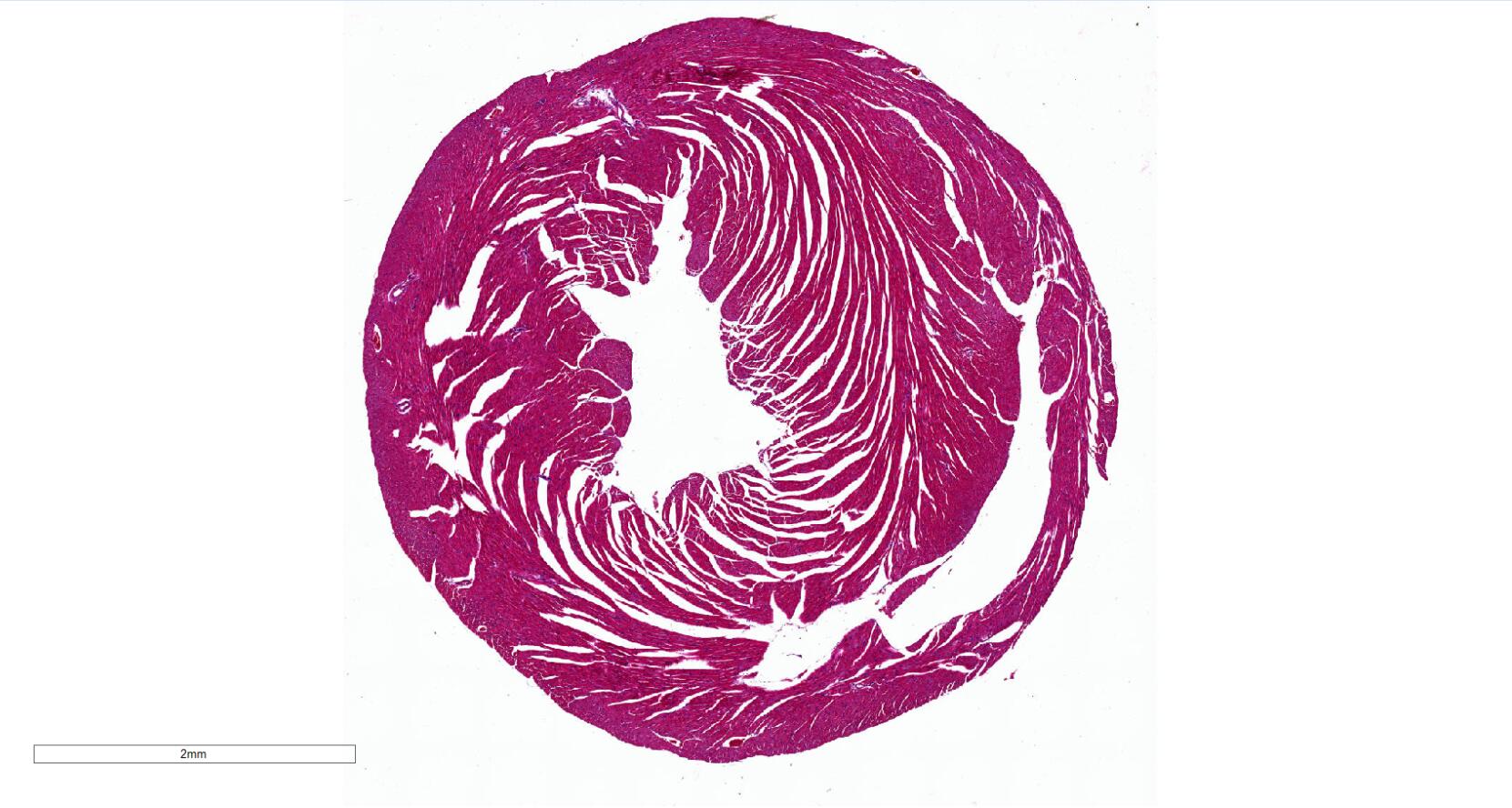

Supplement: Supplementary file 1 [file DataSheet1.ZIP › Additinal files/HE staining/Supplementary Figure S1.A HE/KO AB+Veh 40X.jpg]

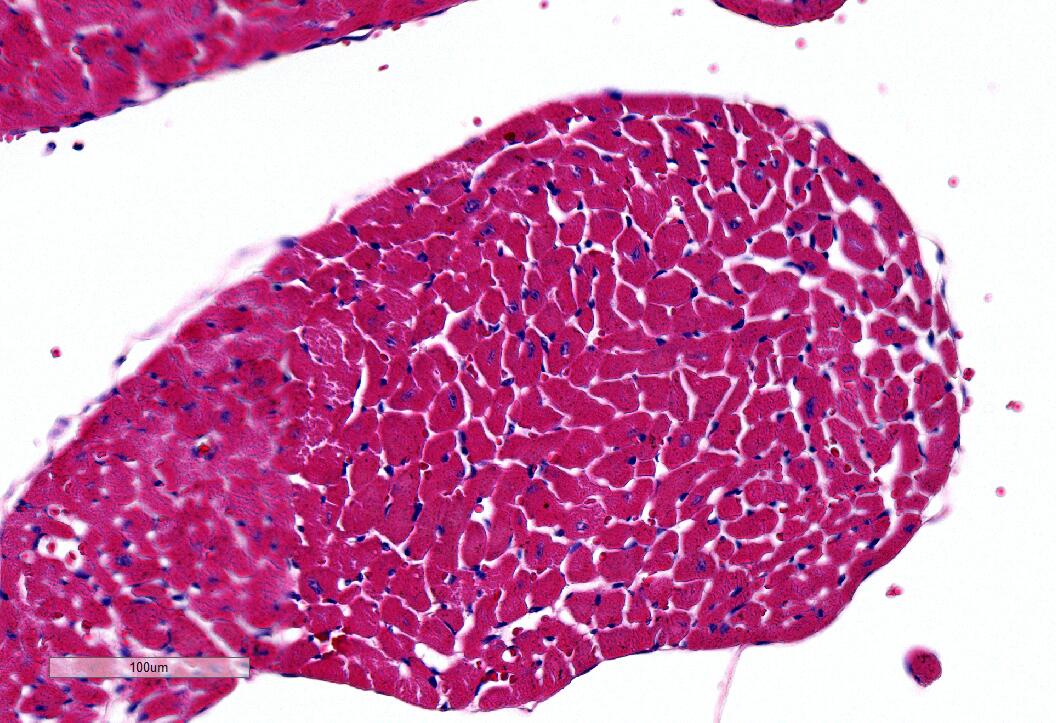

Supplement: Supplementary file 1 [file DataSheet1.ZIP › Additinal files/HE staining/Supplementary Figure S1.A HE/KO Sham+Veh 200X.jpg]

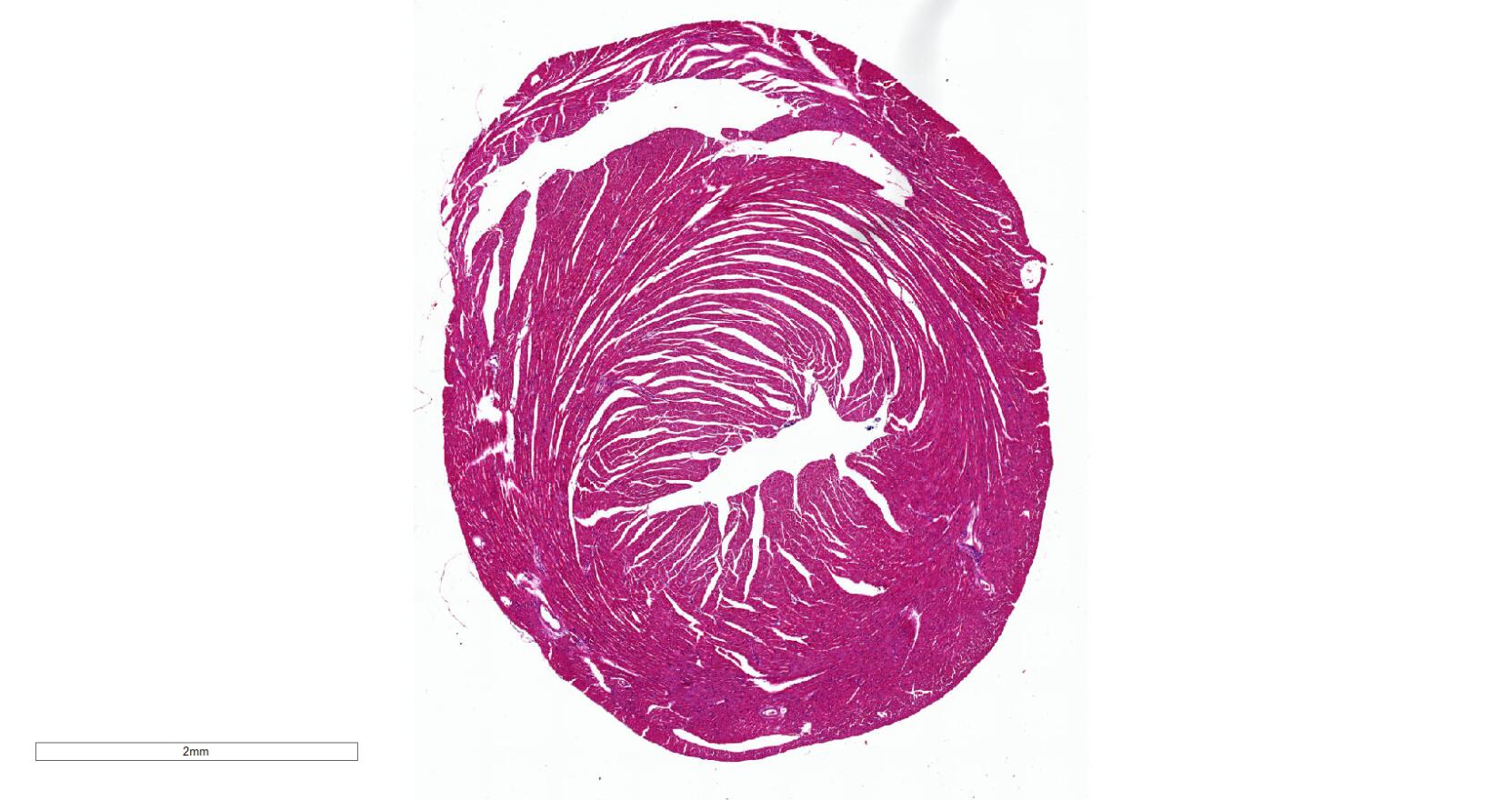

Supplement: Supplementary file 1 [file DataSheet1.ZIP › Additinal files/HE staining/Supplementary Figure S1.A HE/KO Sham+Veh 40X.jpg]

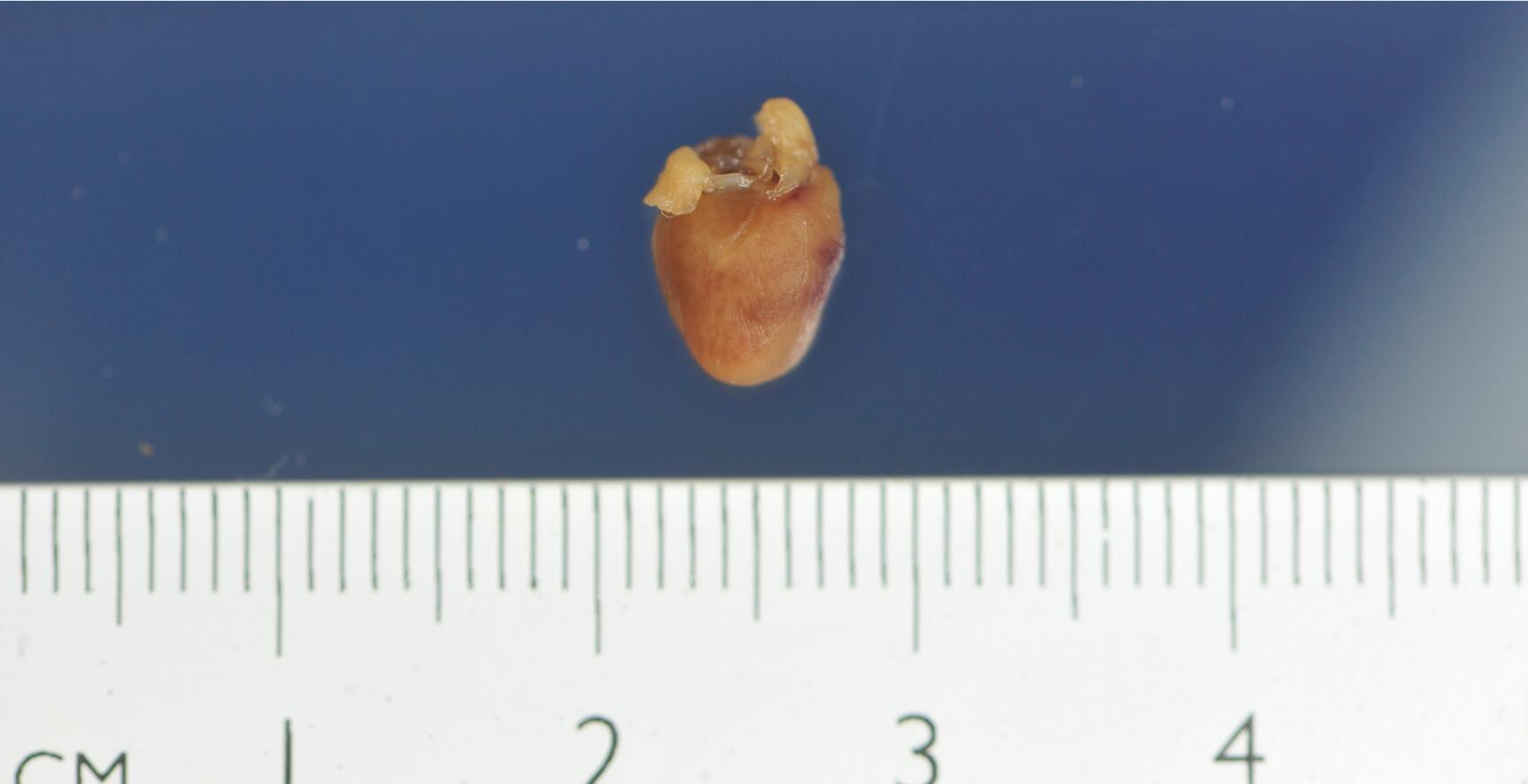

Supplement: Supplementary file 1 [file DataSheet1.ZIP › Additinal files/Heart size/Figure 2.A heart size/AB+LQ.jpg]

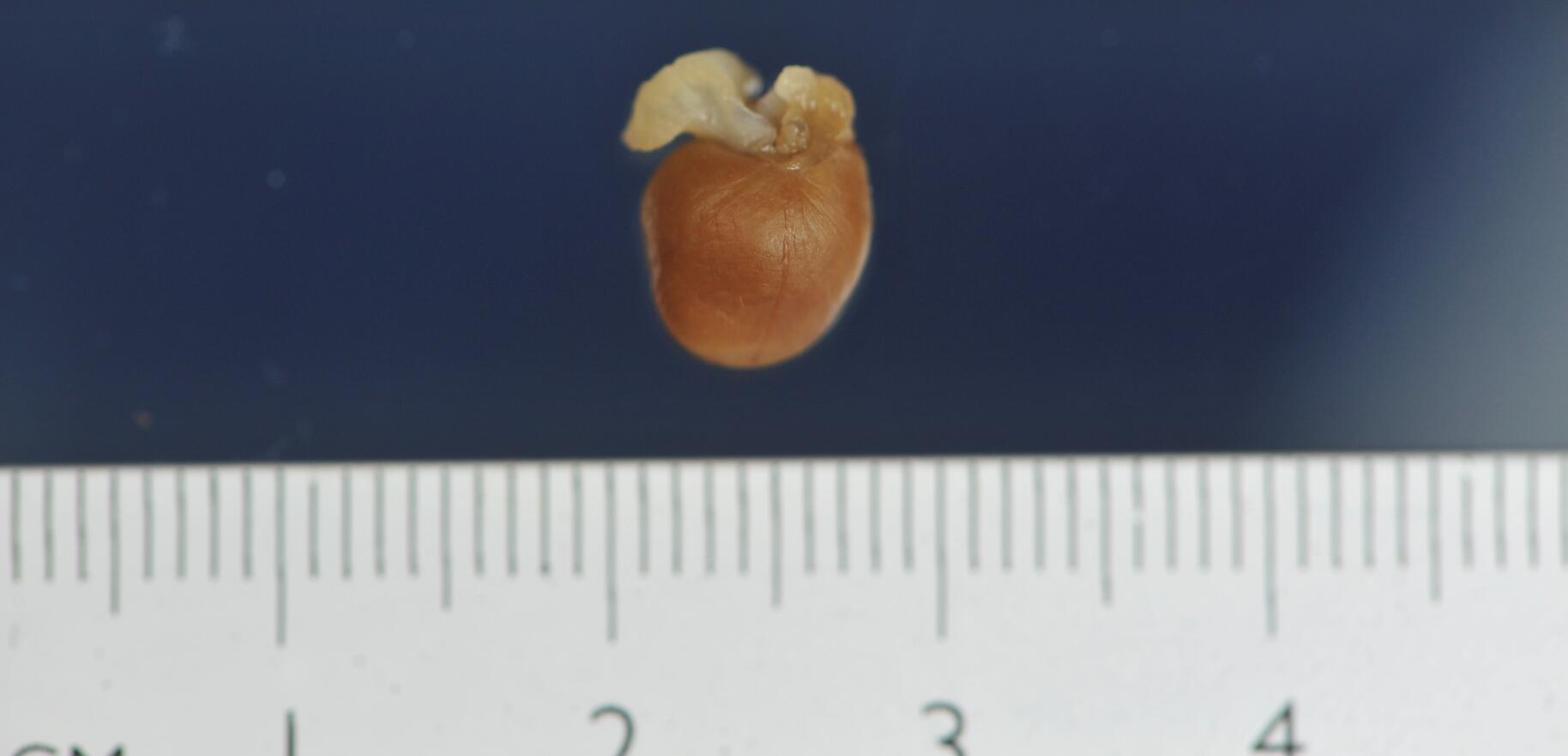

Supplement: Supplementary file 1 [file DataSheet1.ZIP › Additinal files/Heart size/Figure 2.A heart size/AB+Veh.jpg]

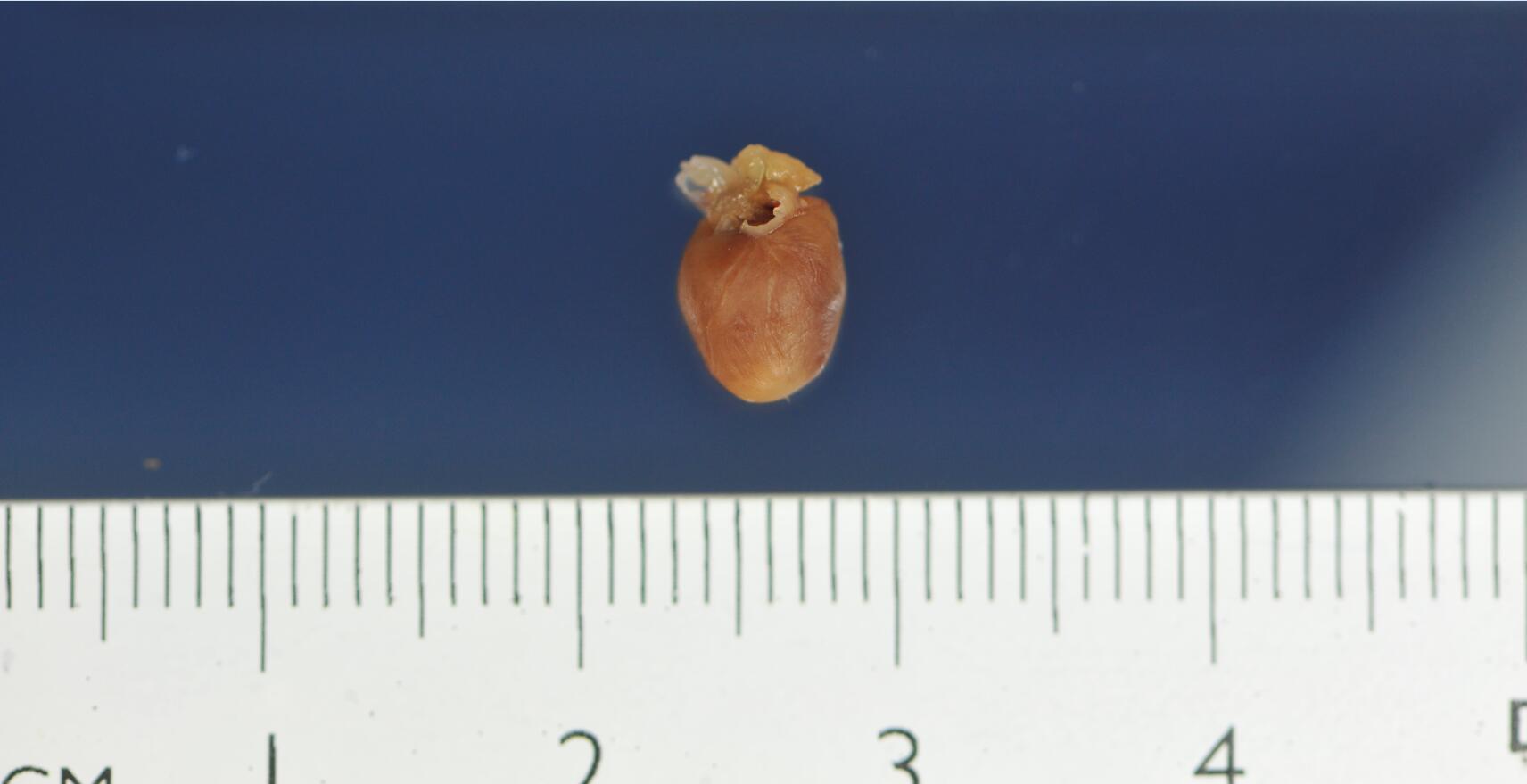

Supplement: Supplementary file 1 [file DataSheet1.ZIP › Additinal files/Heart size/Figure 2.A heart size/Sham+LQ.jpg]

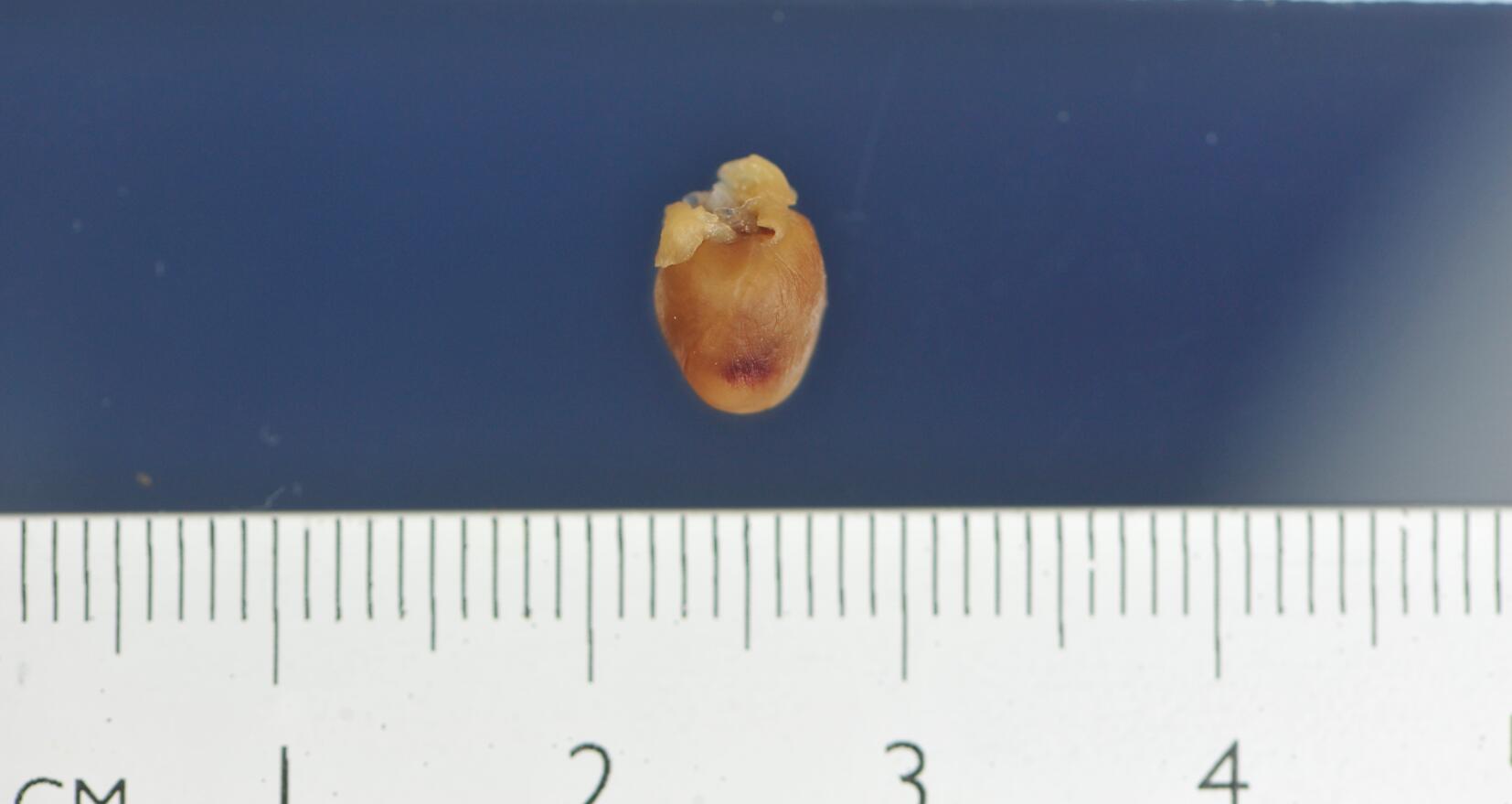

Supplement: Supplementary file 1 [file DataSheet1.ZIP › Additinal files/Heart size/Figure 2.A heart size/Sham+Veh.jpg]

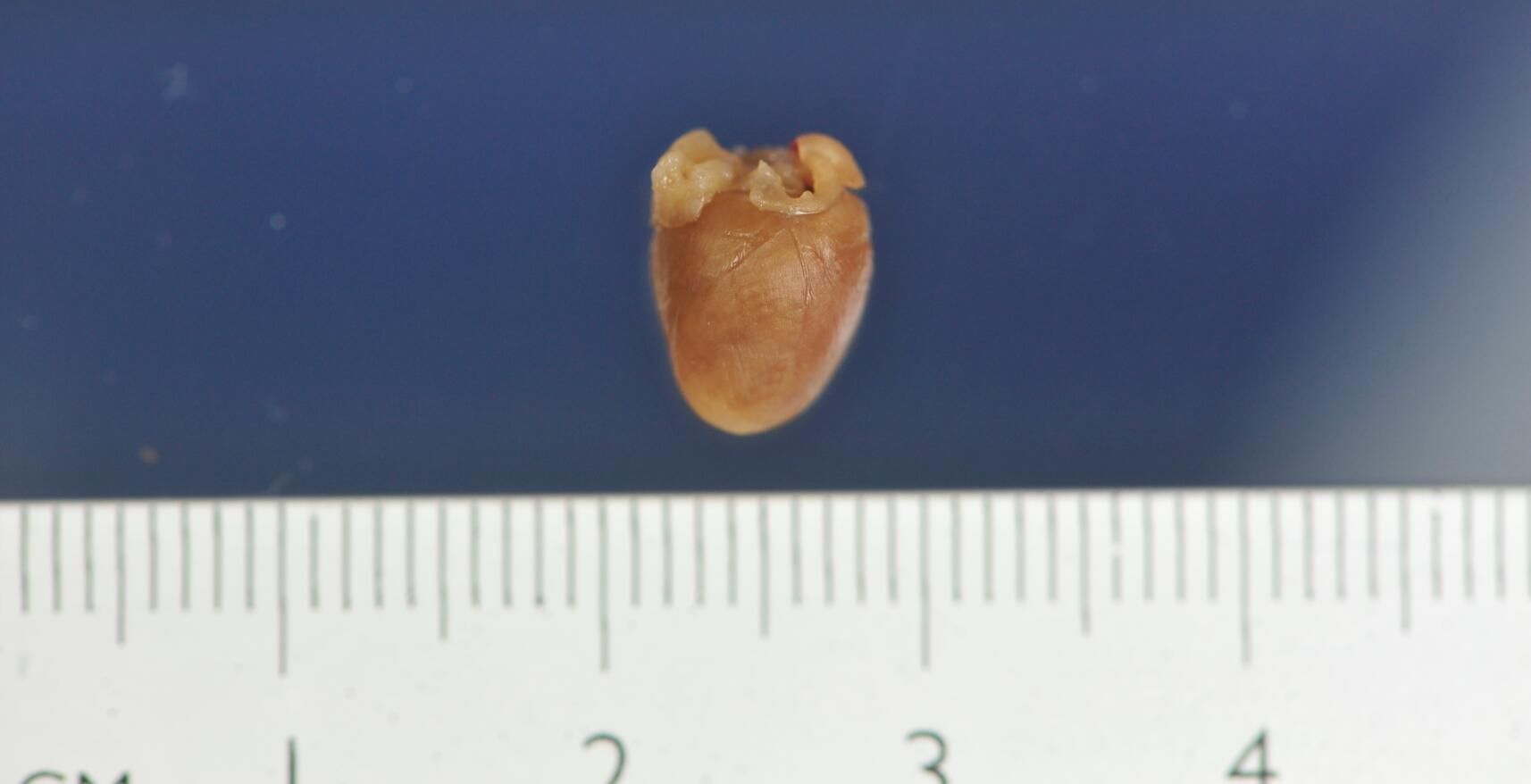

Supplement: Supplementary file 1 [file DataSheet1.ZIP › Additinal files/Heart size/Supplementary Figure S1.A heart size/KO AB+LQ.jpg]

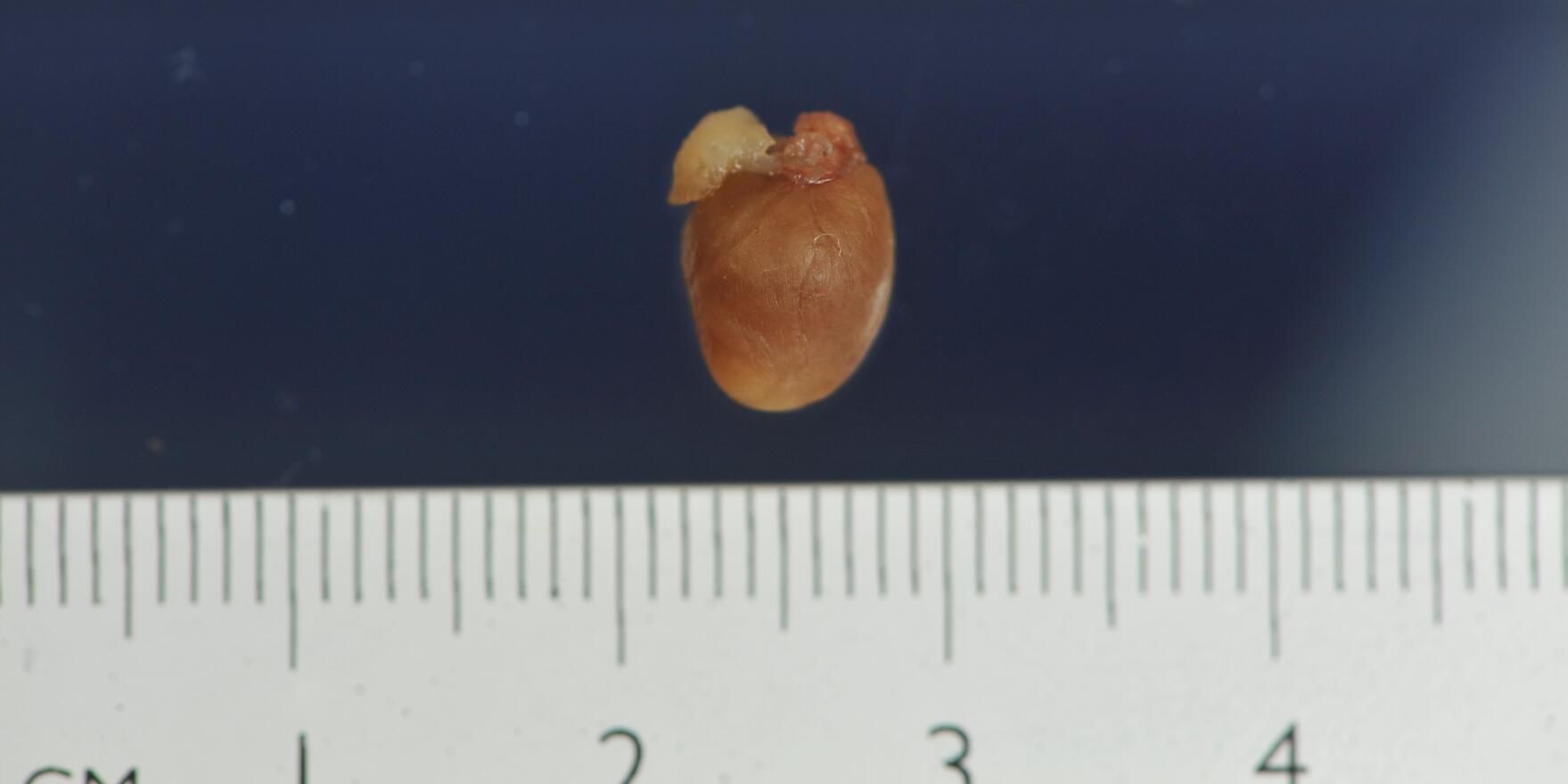

Supplement: Supplementary file 1 [file DataSheet1.ZIP › Additinal files/Heart size/Supplementary Figure S1.A heart size/KO AB+Veh.jpg]

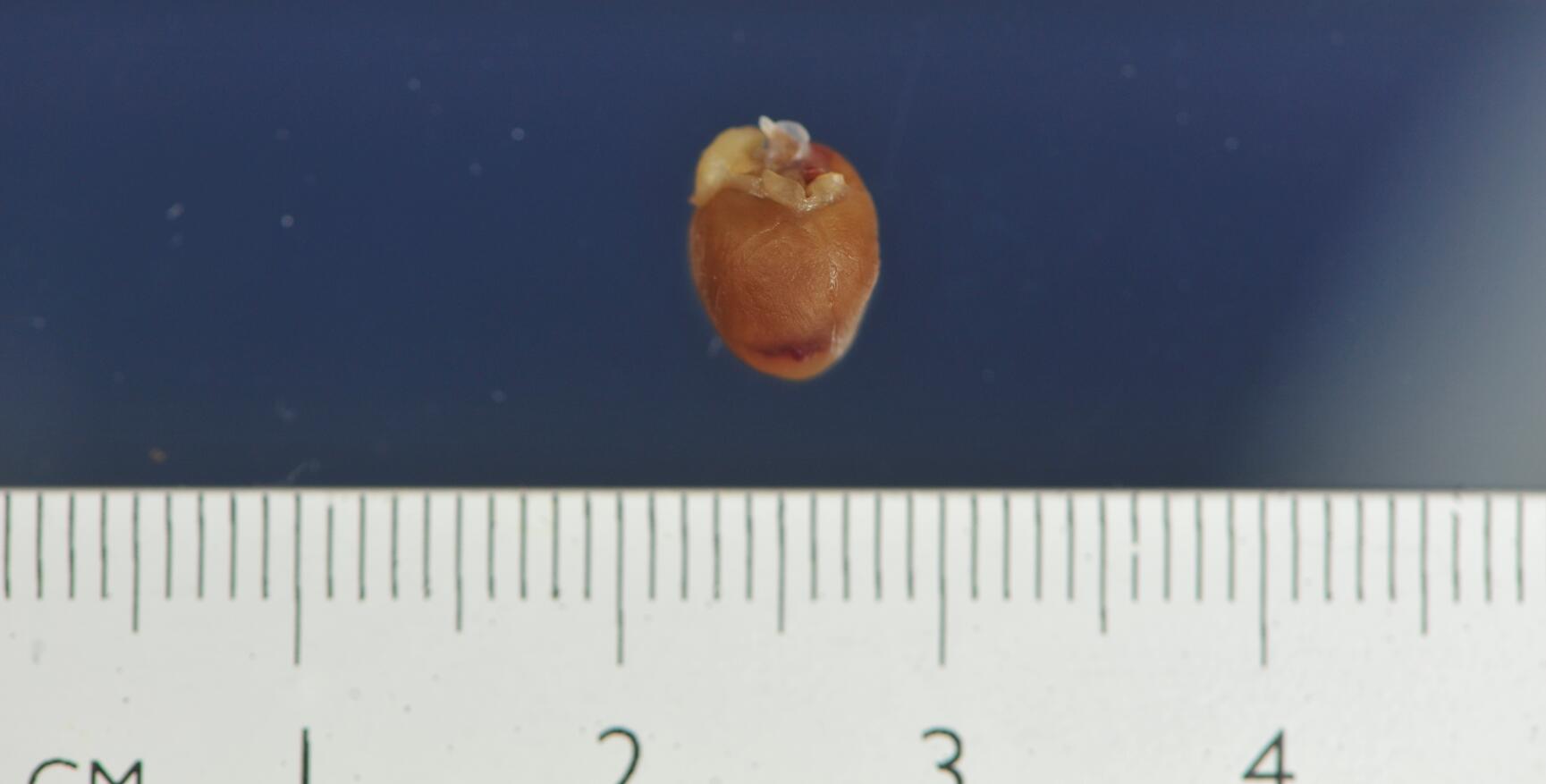

Supplement: Supplementary file 1 [file DataSheet1.ZIP › Additinal files/Heart size/Supplementary Figure S1.A heart size/KO Sham+Veh.jpg]

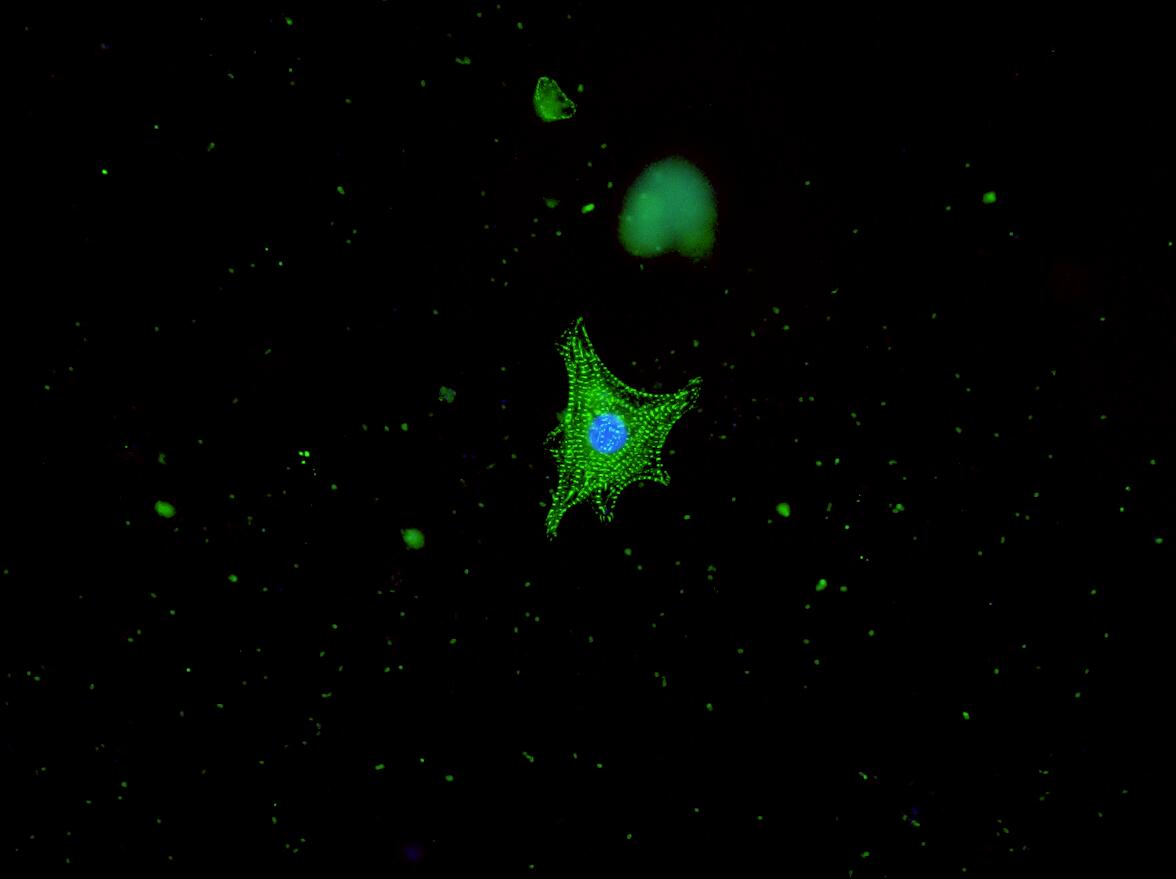

Supplement: Supplementary file 1 [file DataSheet1.ZIP › Additinal files/IF a-actinin/Figure 7.B a-actinin/Ang II+LQ.jpg]

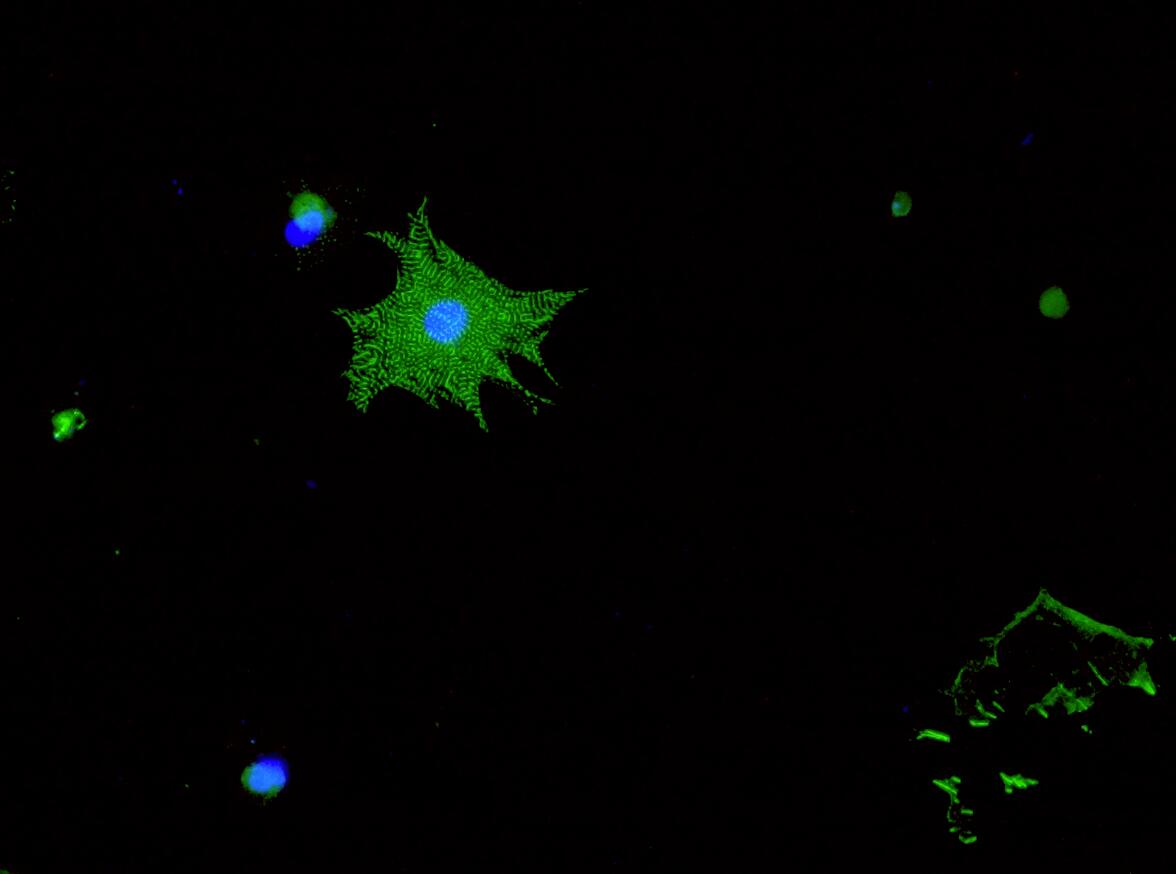

Supplement: Supplementary file 1 [file DataSheet1.ZIP › Additinal files/IF a-actinin/Figure 7.B a-actinin/Ang II+Veh.jpg]

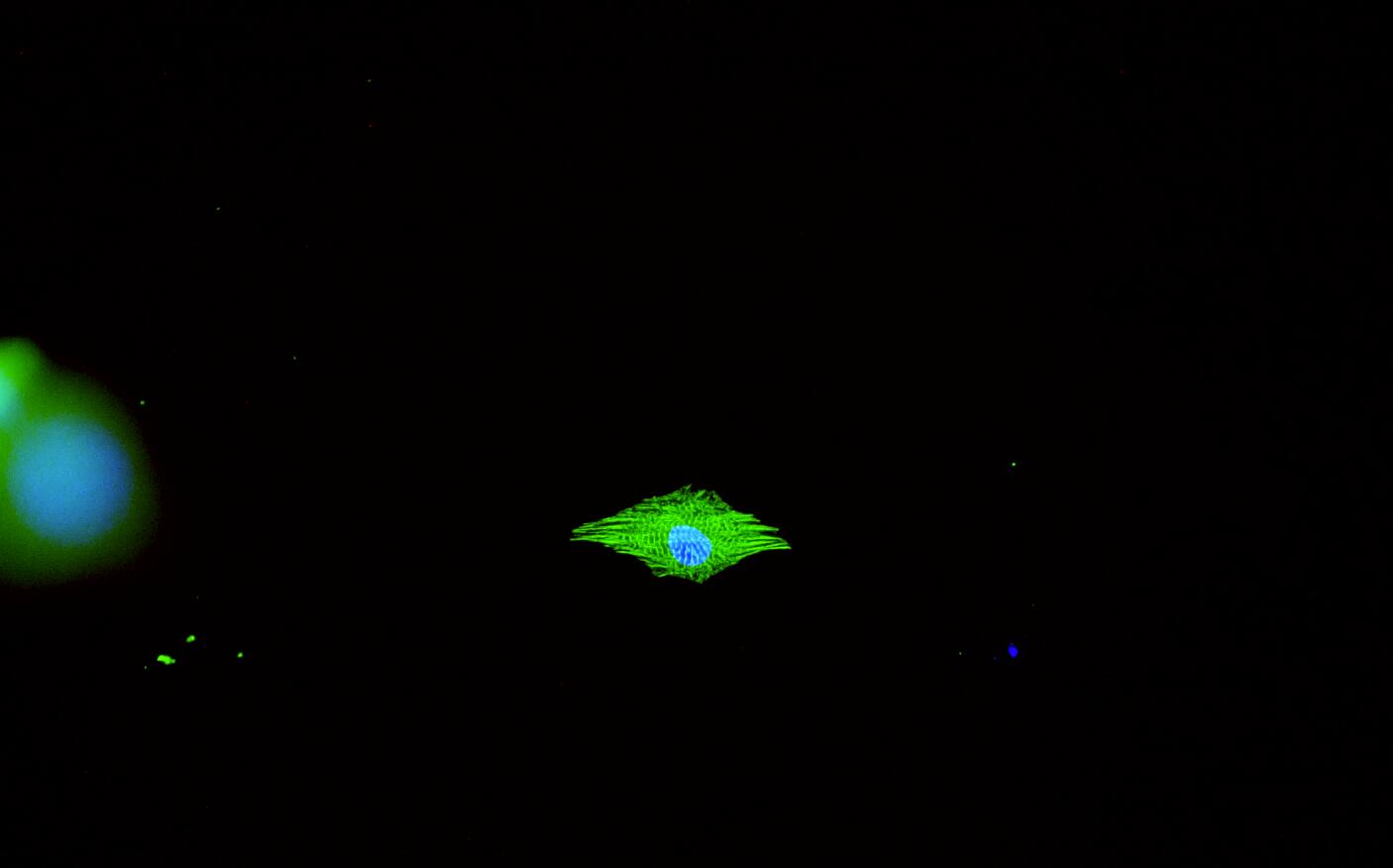

Supplement: Supplementary file 1 [file DataSheet1.ZIP › Additinal files/IF a-actinin/Figure 7.B a-actinin/PBS+LQ.jpg]

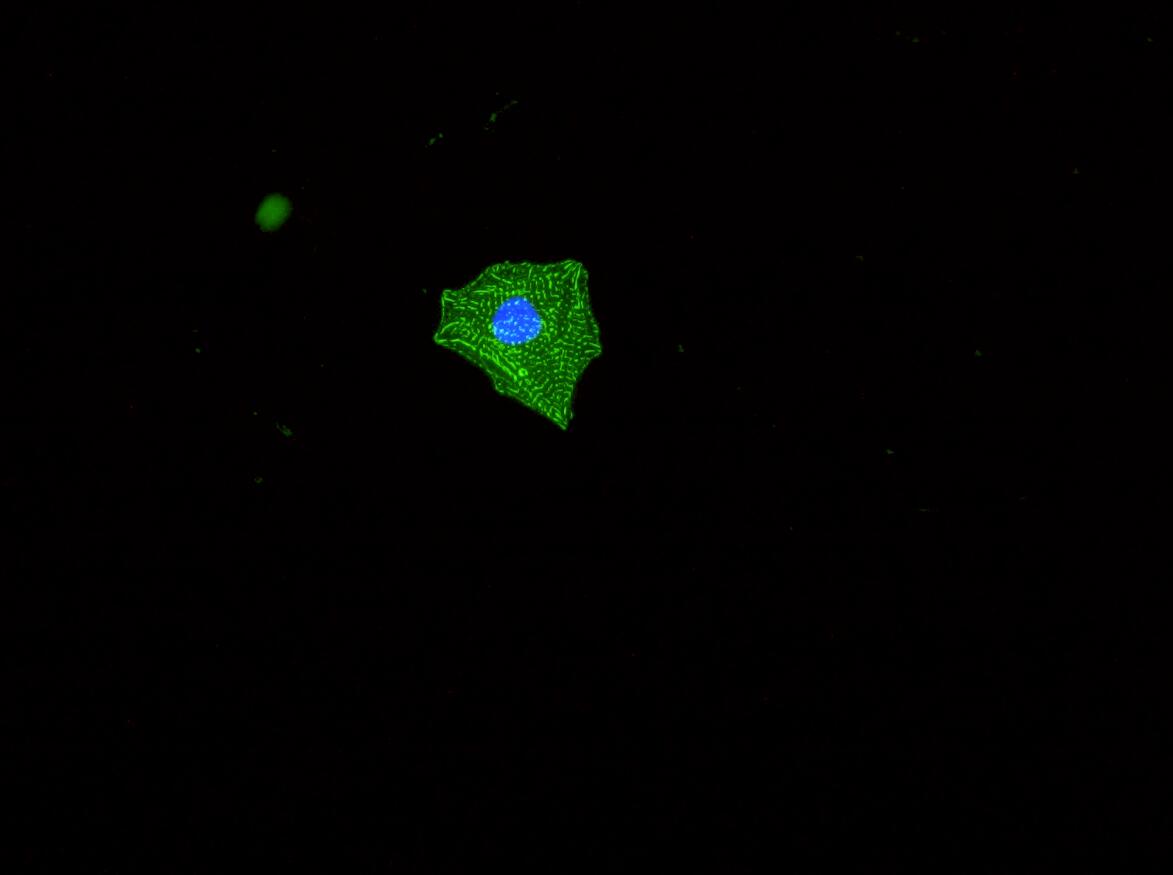

Supplement: Supplementary file 1 [file DataSheet1.ZIP › Additinal files/IF a-actinin/Figure 7.B a-actinin/PBS+Veh.jpg]

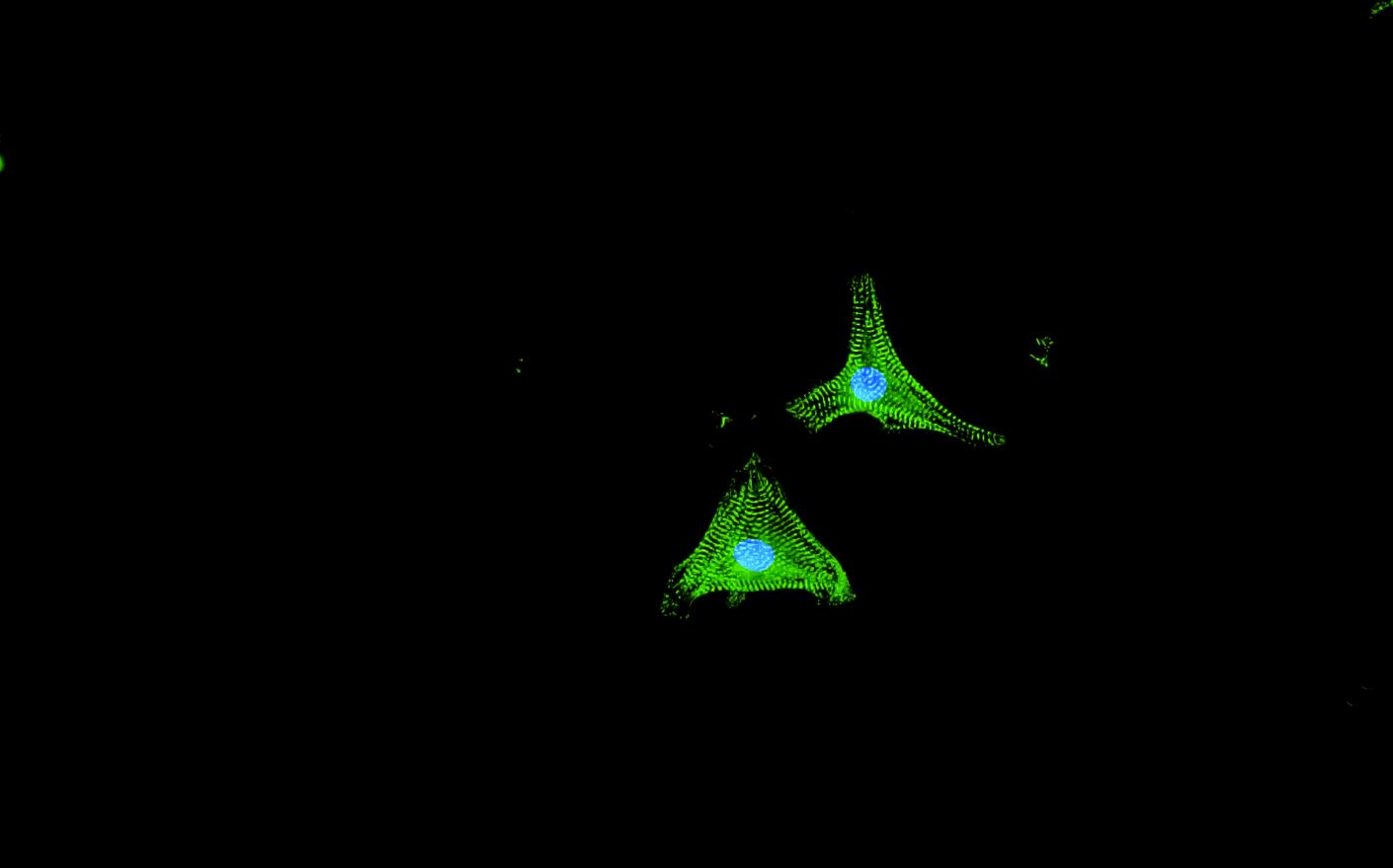

Supplement: Supplementary file 1 [file DataSheet1.ZIP › Additinal files/IF a-actinin/Figure 8.A a-actinin/LQ+Ang II+CpC-.jpg]

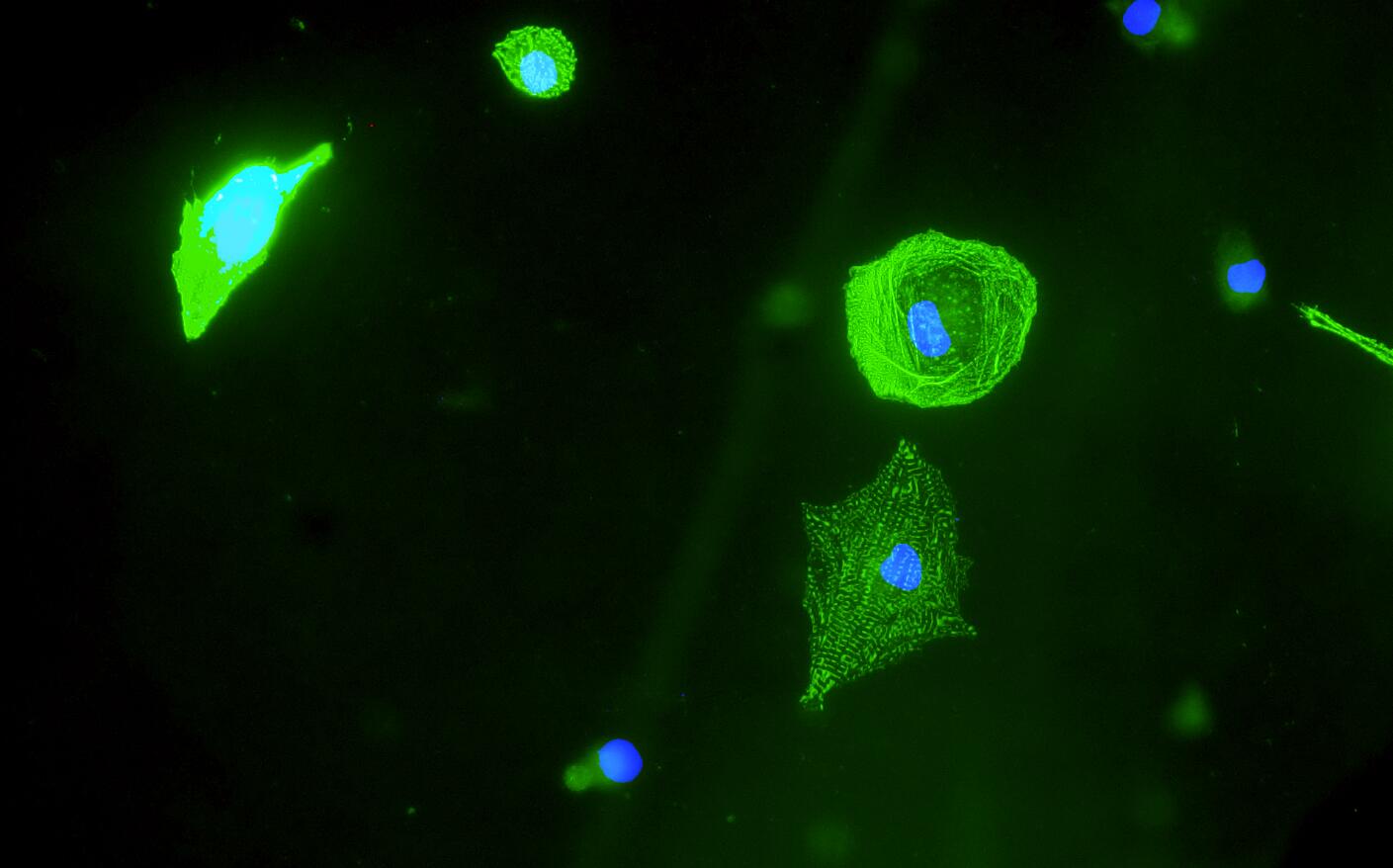

Supplement: Supplementary file 1 [file DataSheet1.ZIP › Additinal files/IF a-actinin/Figure 8.A a-actinin/LQ+Ang II+CpC+.jpg]

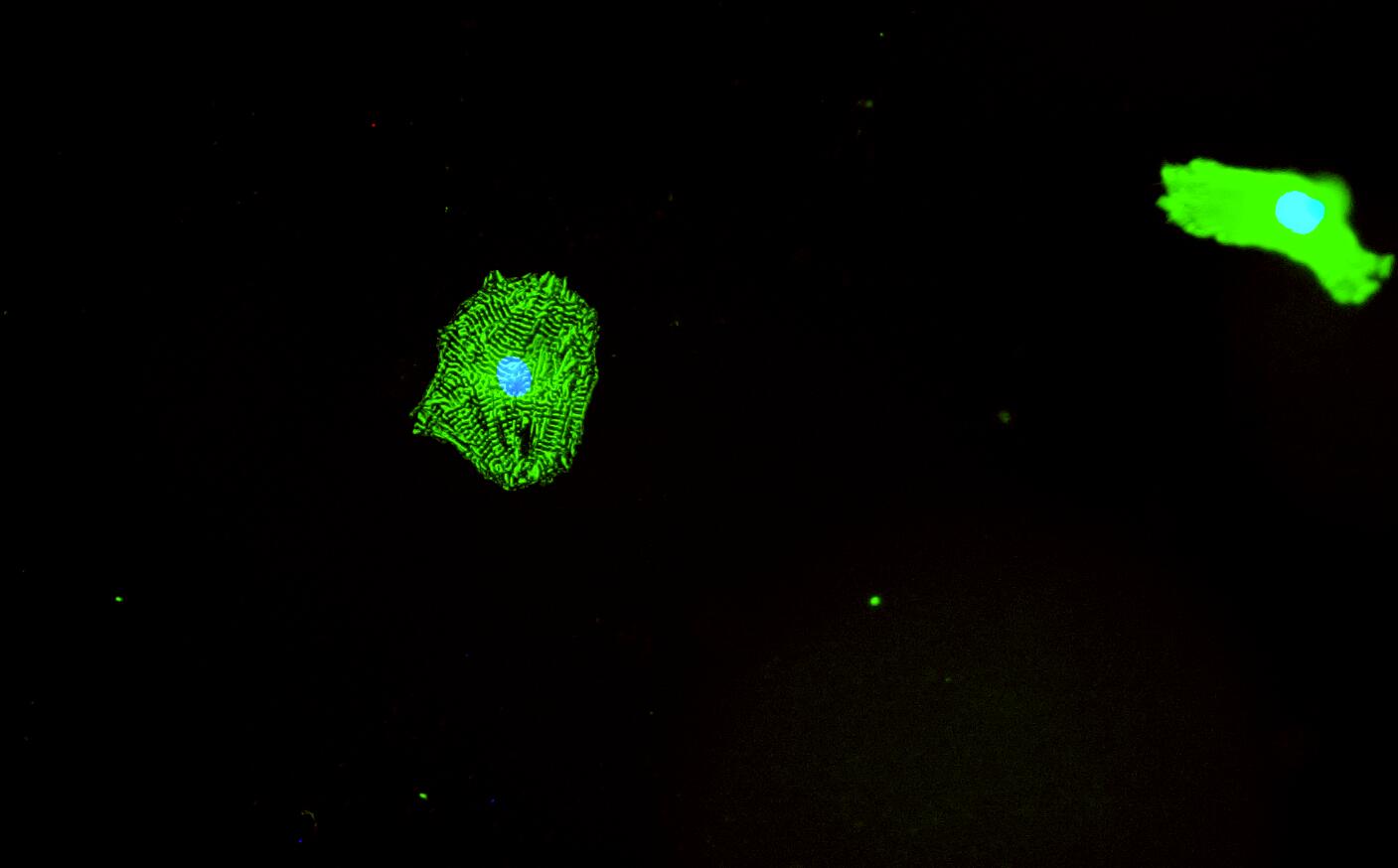

Supplement: Supplementary file 1 [file DataSheet1.ZIP › Additinal files/IF a-actinin/Figure 8.A a-actinin/LQ-Ang II+CpC-.jpg]

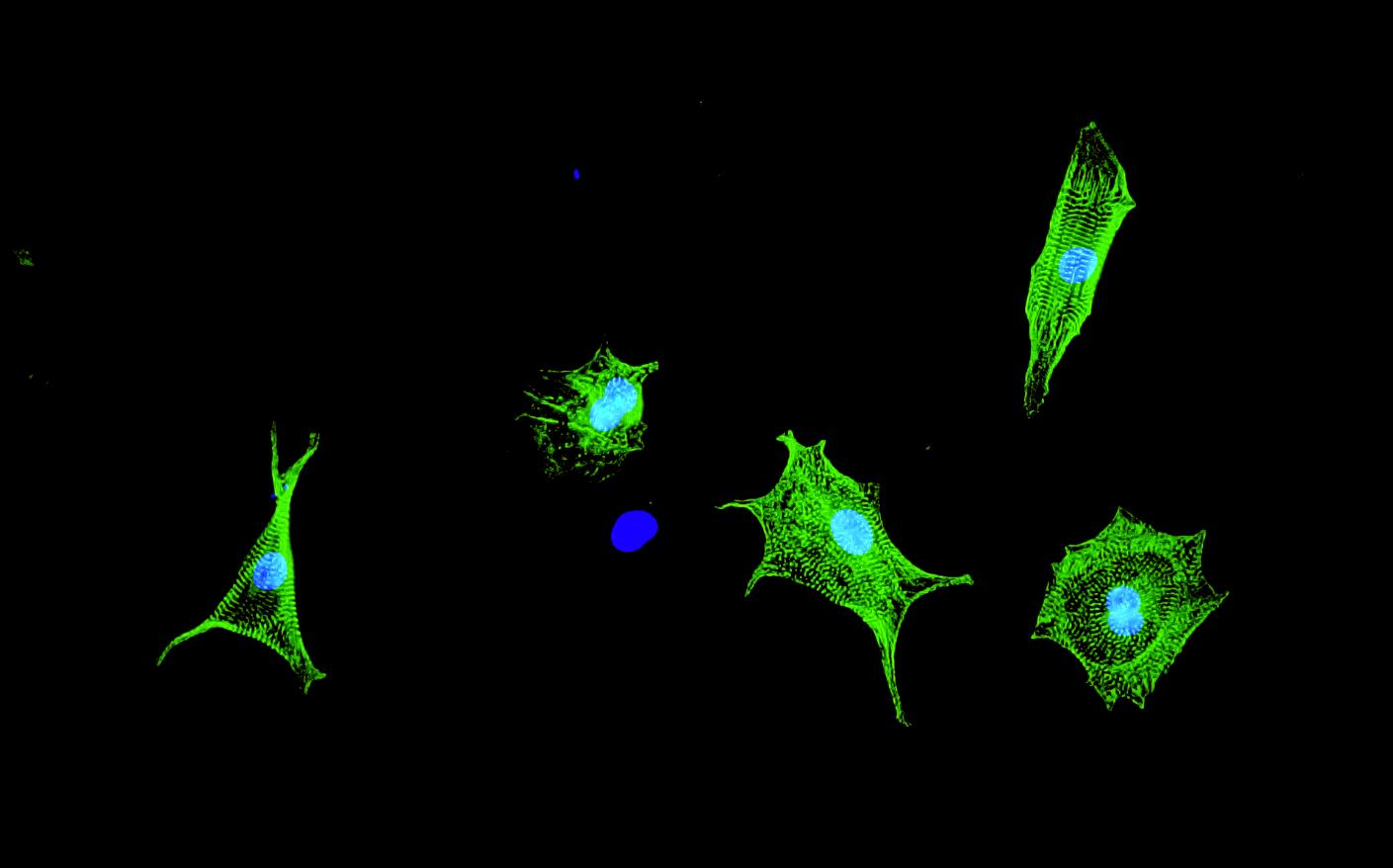

Supplement: Supplementary file 1 [file DataSheet1.ZIP › Additinal files/IF a-actinin/Figure 8.A a-actinin/LQ-Ang II+CpC+.jpg]

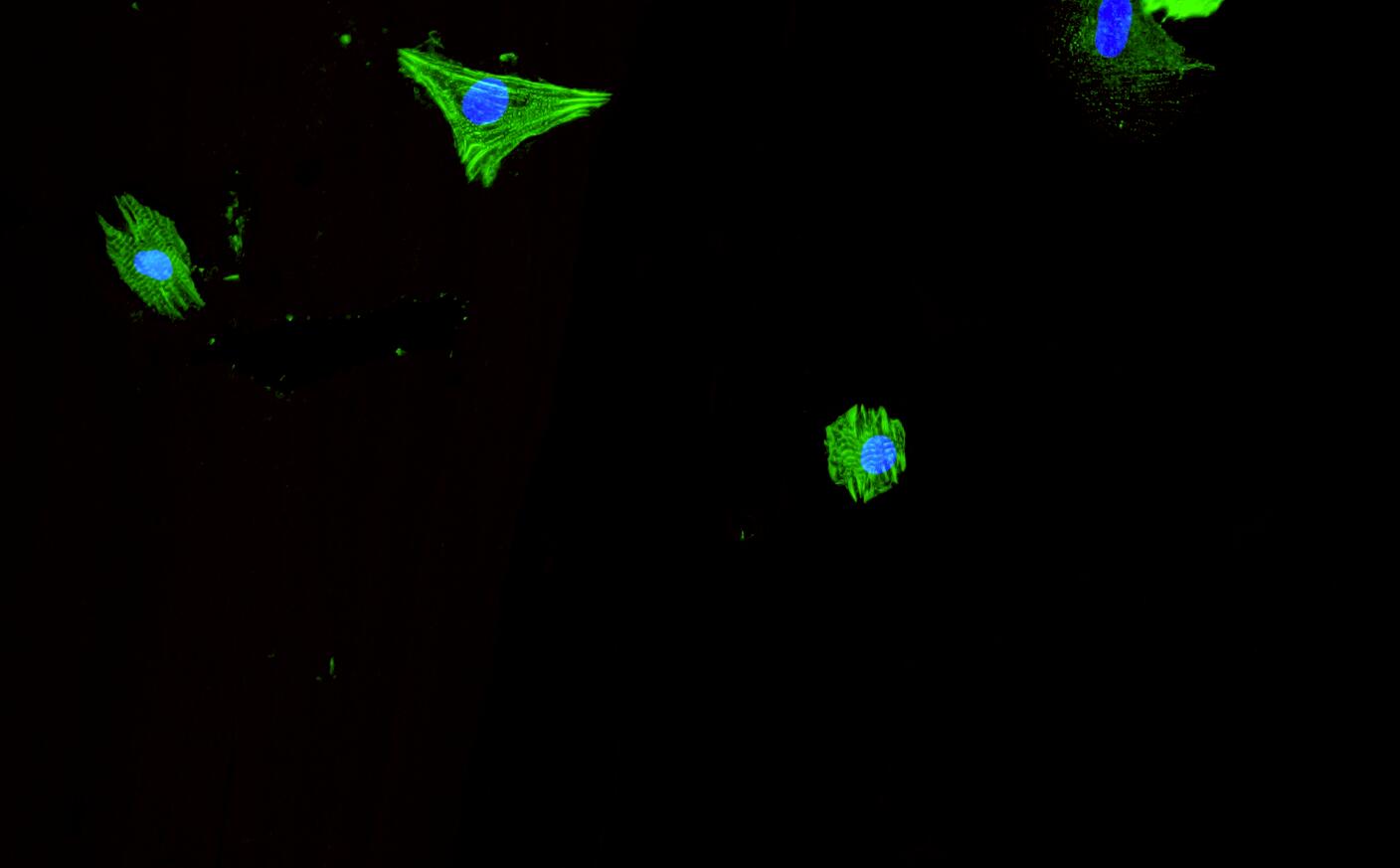

Supplement: Supplementary file 1 [file DataSheet1.ZIP › Additinal files/IF a-actinin/Figure 8.A a-actinin/LQ-Ang II-CpC-.jpg]

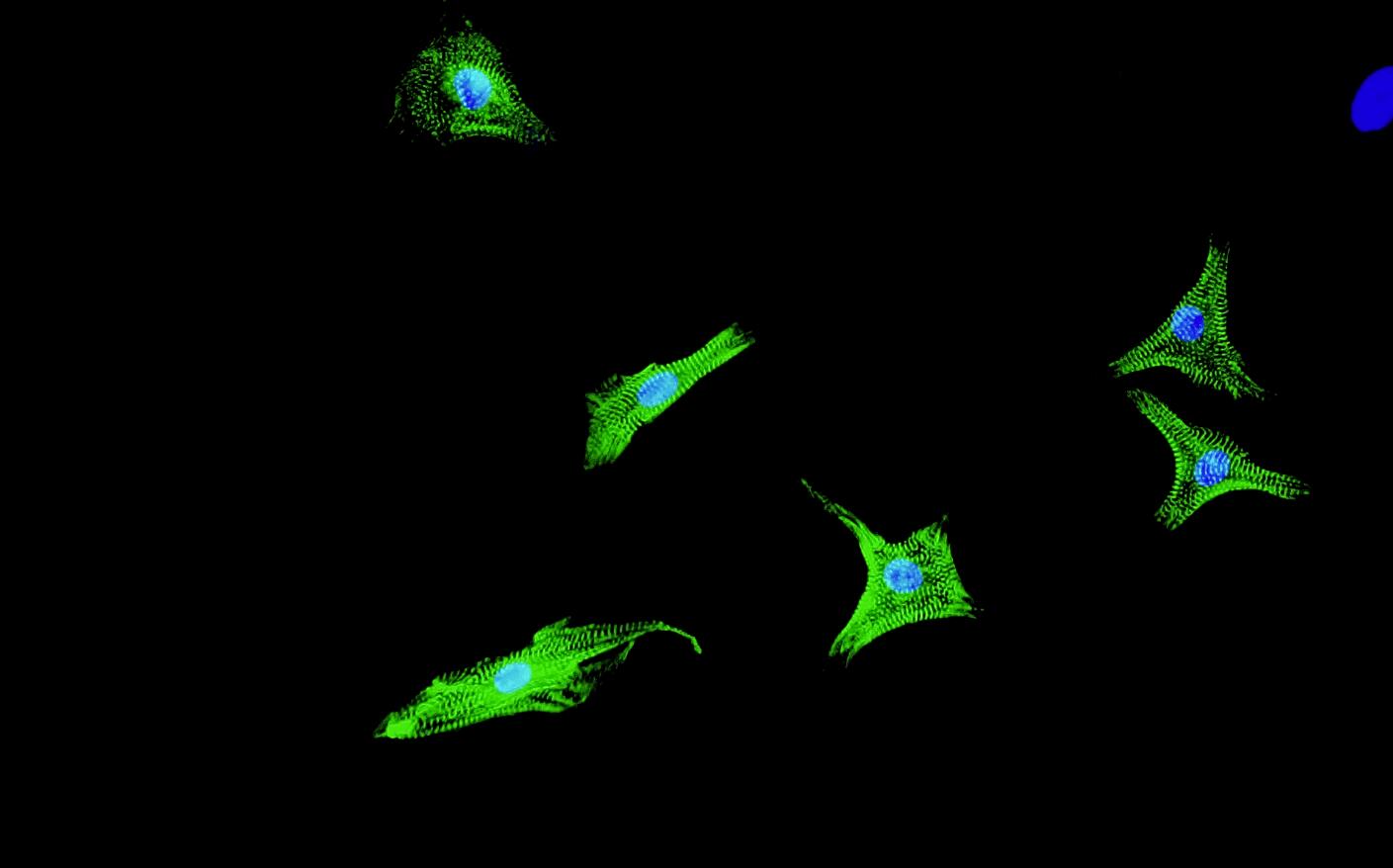

Supplement: Supplementary file 1 [file DataSheet1.ZIP › Additinal files/IF a-actinin/Figure 8.A a-actinin/LQ-Ang II-CpC+.jpg]

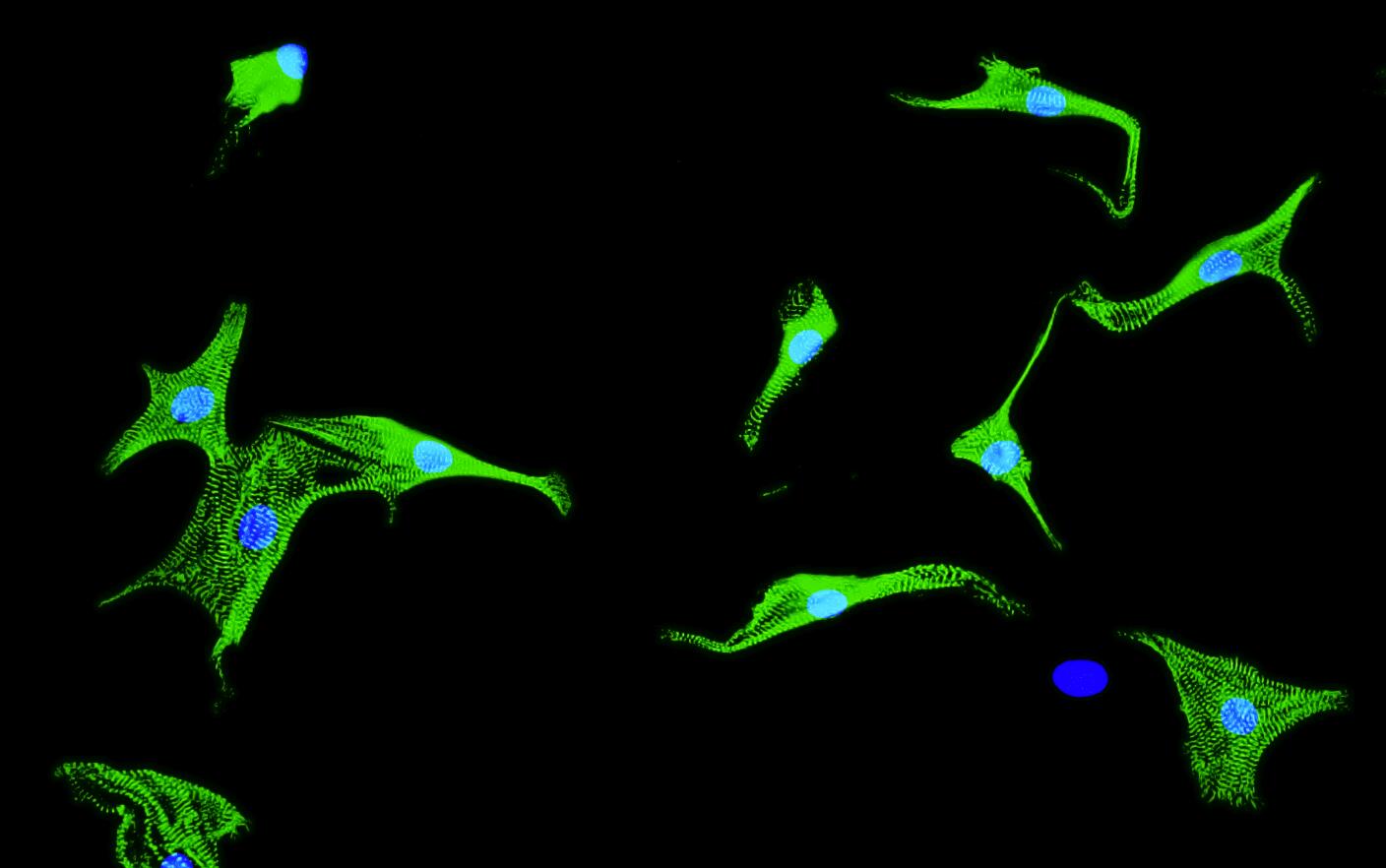

Supplement: Supplementary file 1 [file DataSheet1.ZIP › Additinal files/IF a-actinin/Figure 9.F a-actinin/LQ+Ang II+H89-.jpg]

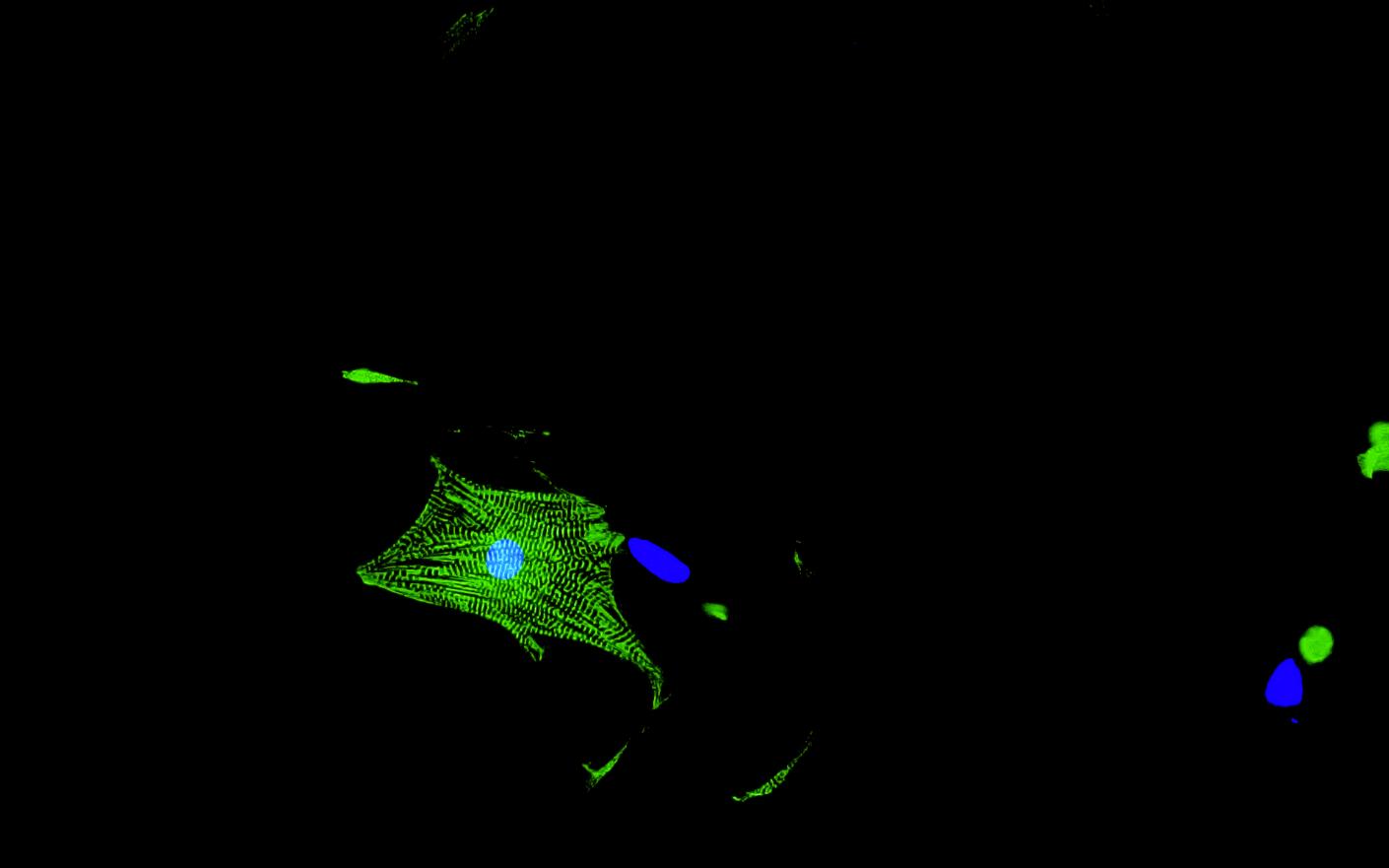

Supplement: Supplementary file 1 [file DataSheet1.ZIP › Additinal files/IF a-actinin/Figure 9.F a-actinin/LQ+Ang II+H89+.jpg]

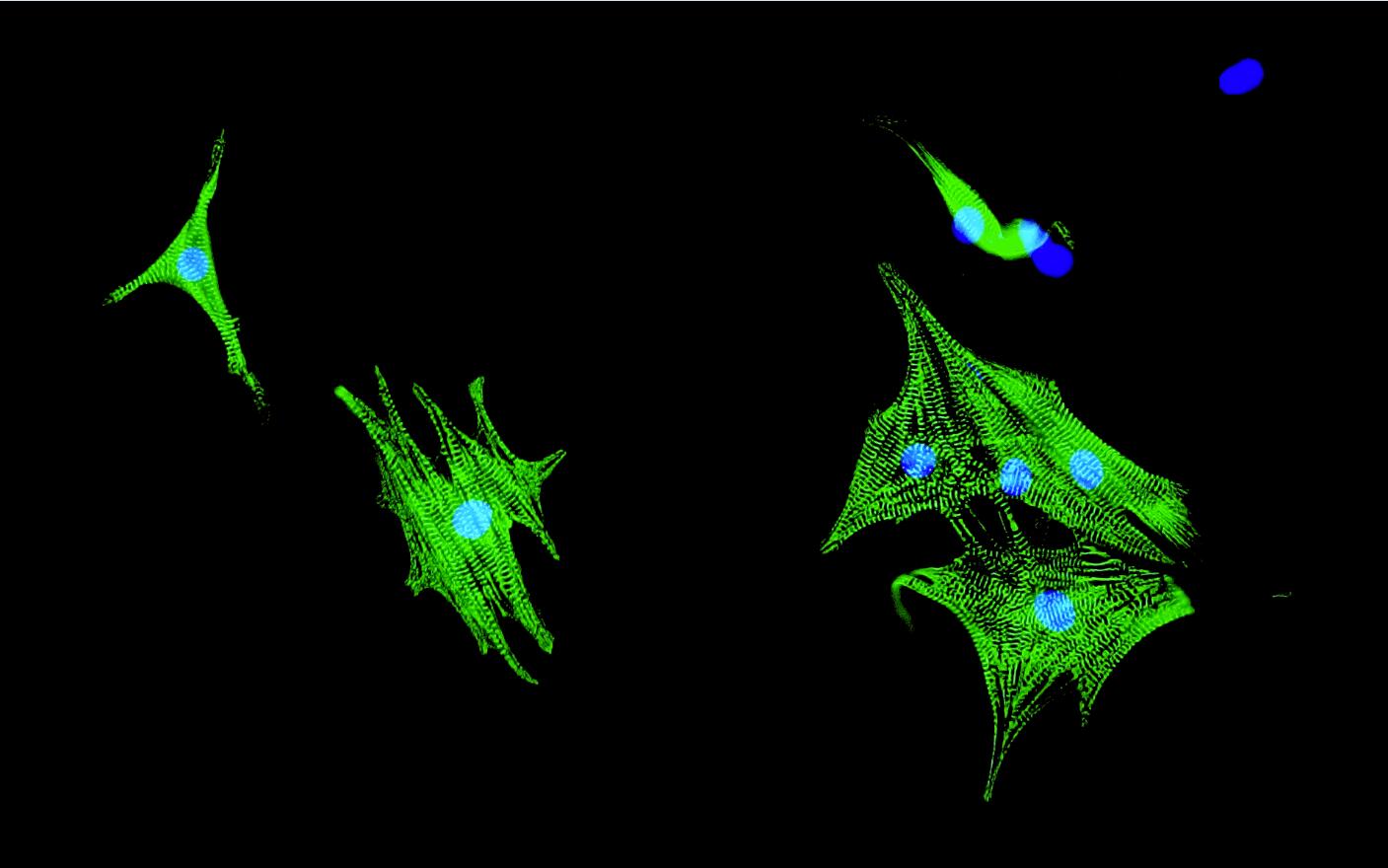

Supplement: Supplementary file 1 [file DataSheet1.ZIP › Additinal files/IF a-actinin/Figure 9.F a-actinin/LQ-Ang II+H89-.jpg]

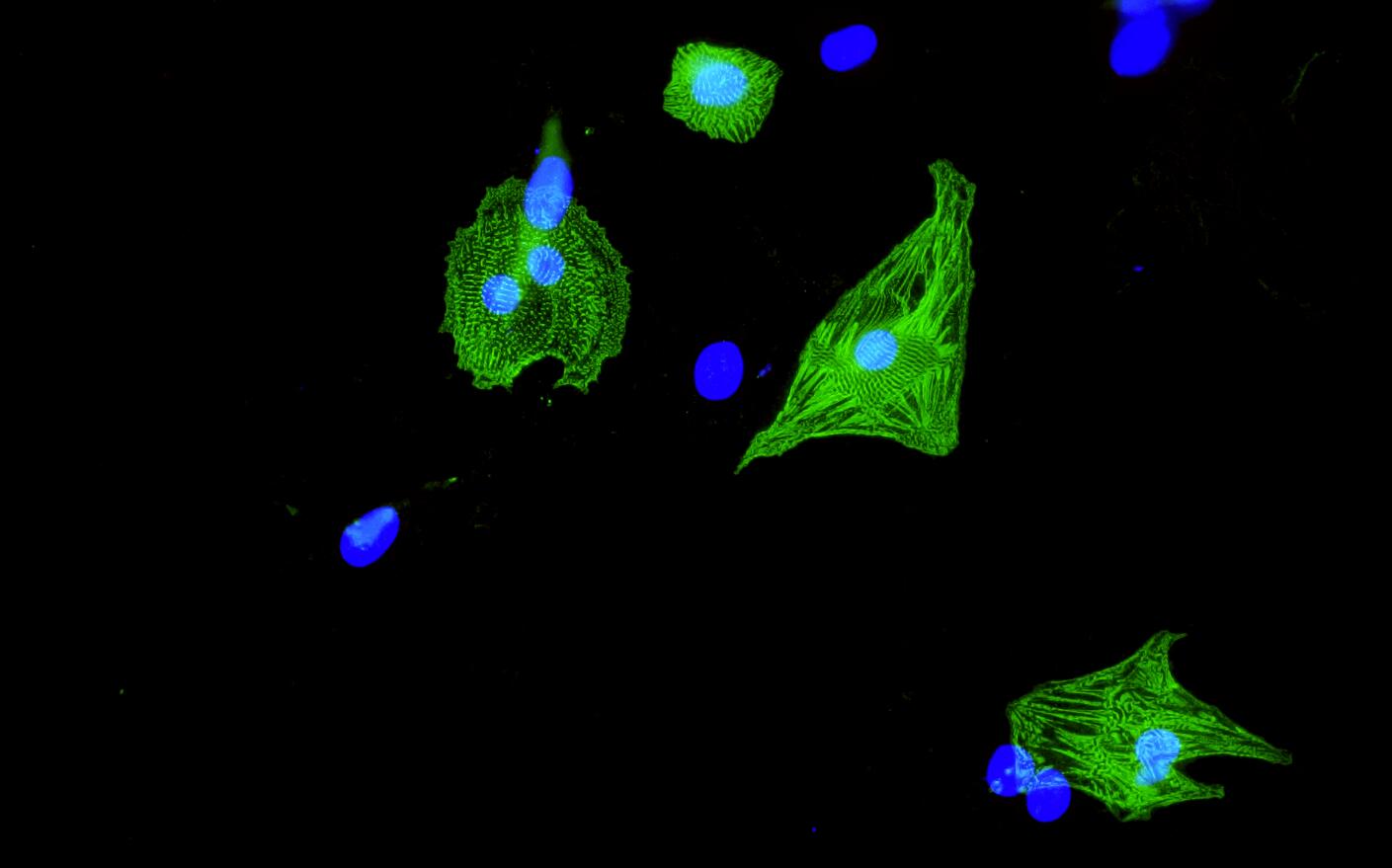

Supplement: Supplementary file 1 [file DataSheet1.ZIP › Additinal files/IF a-actinin/Figure 9.F a-actinin/LQ-Ang II+H89+.jpg]

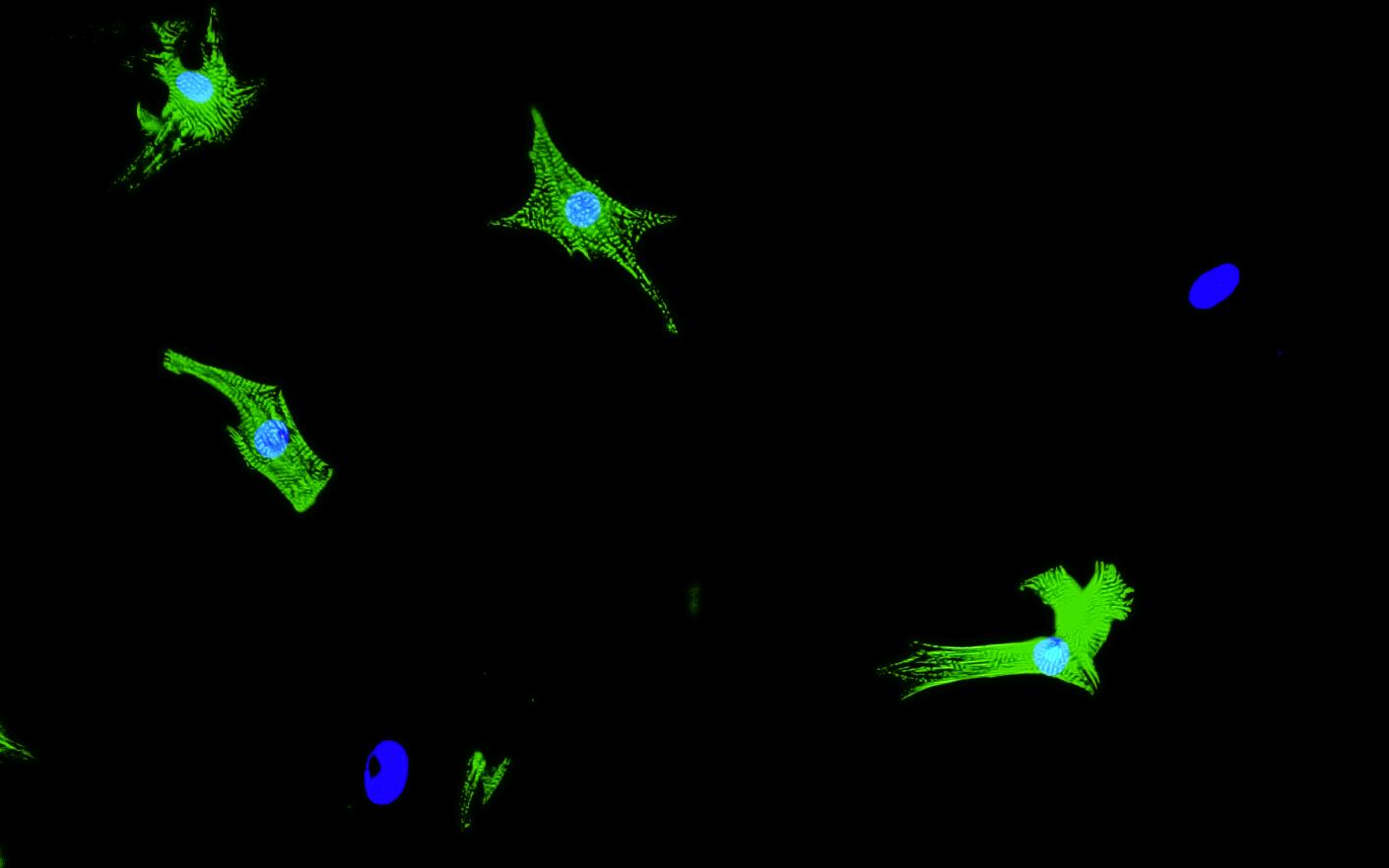

Supplement: Supplementary file 1 [file DataSheet1.ZIP › Additinal files/IF a-actinin/Figure 9.F a-actinin/LQ-Ang II-H89-.jpg]

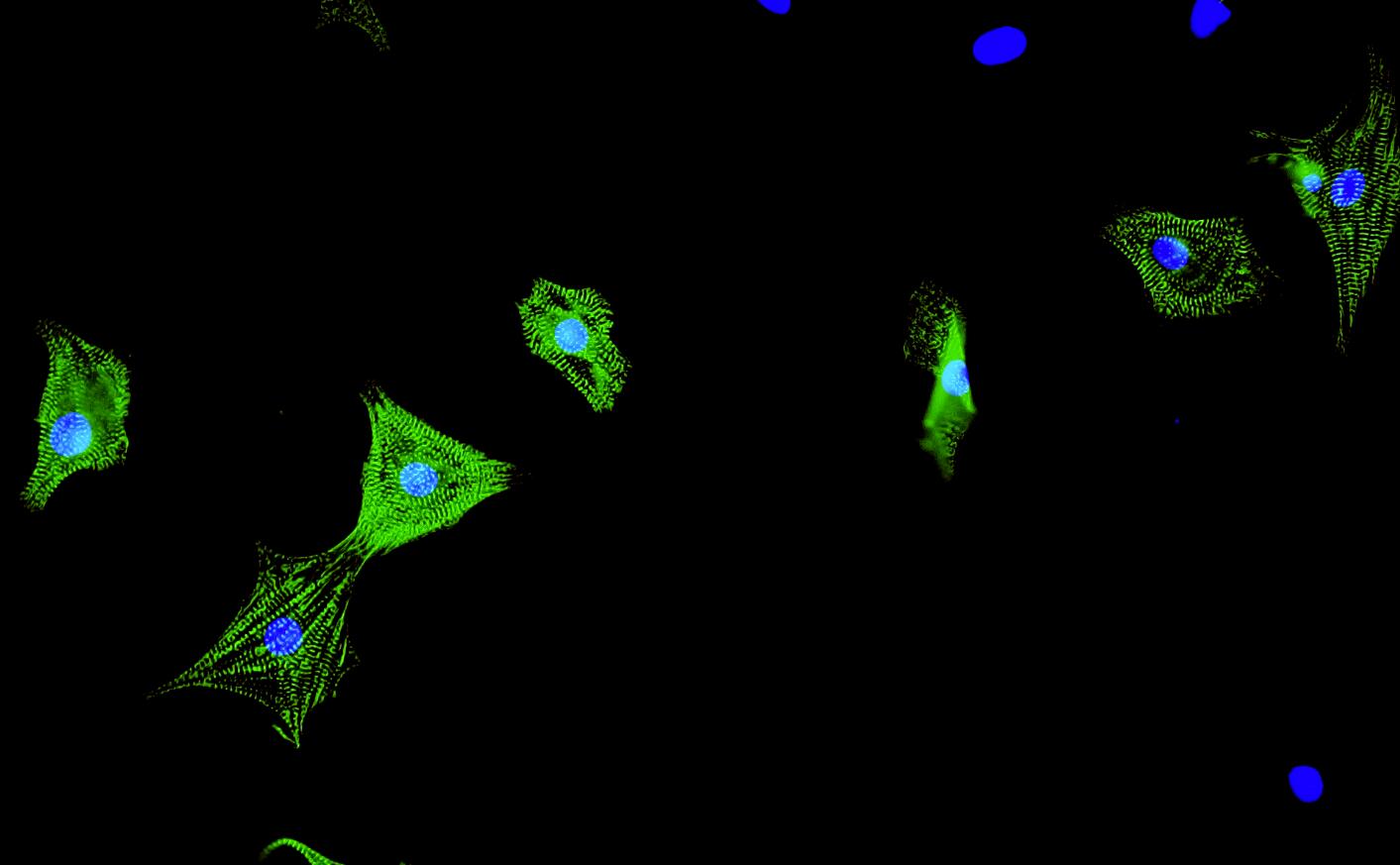

Supplement: Supplementary file 1 [file DataSheet1.ZIP › Additinal files/IF a-actinin/Figure 9.F a-actinin/LQ-Ang II-H89+.jpg]

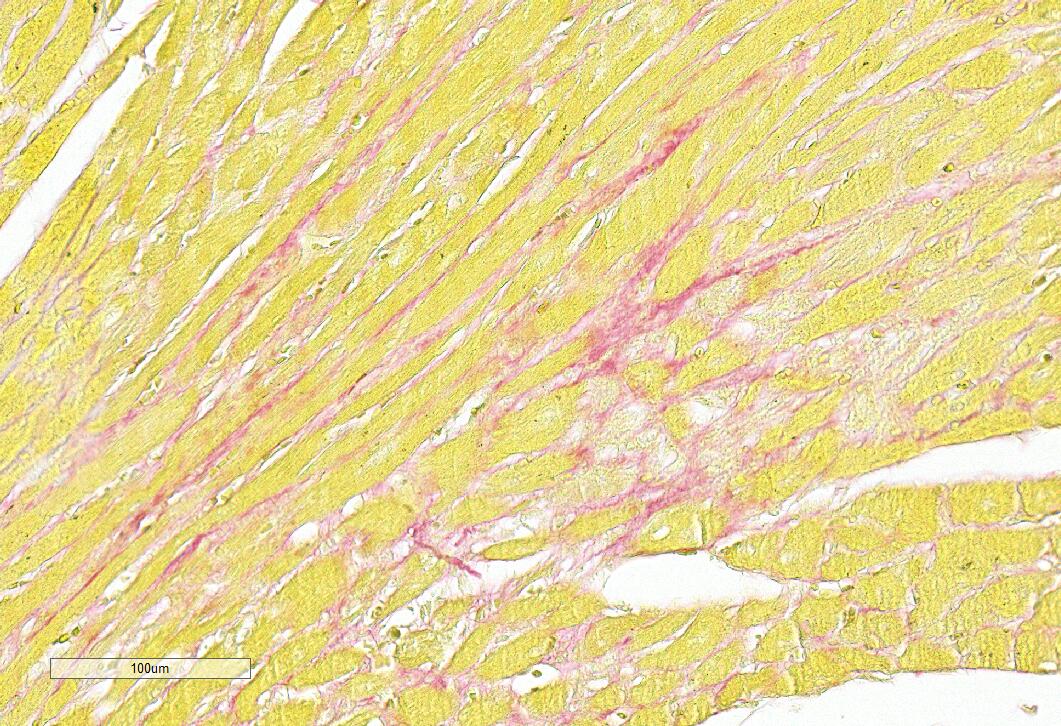

Supplement: Supplementary file 1 [file DataSheet1.ZIP › Additinal files/PSR staining/Figure 3.A PSR/AB+LQ interstitial.jpg]

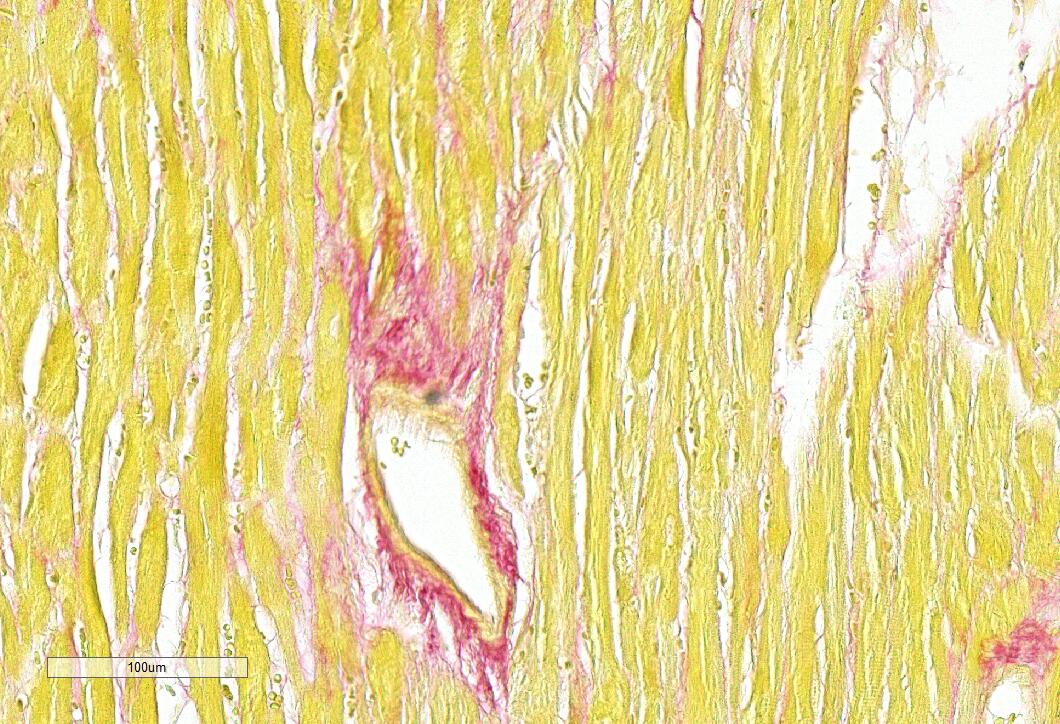

Supplement: Supplementary file 1 [file DataSheet1.ZIP › Additinal files/PSR staining/Figure 3.A PSR/AB+LQ perivascular.jpg]

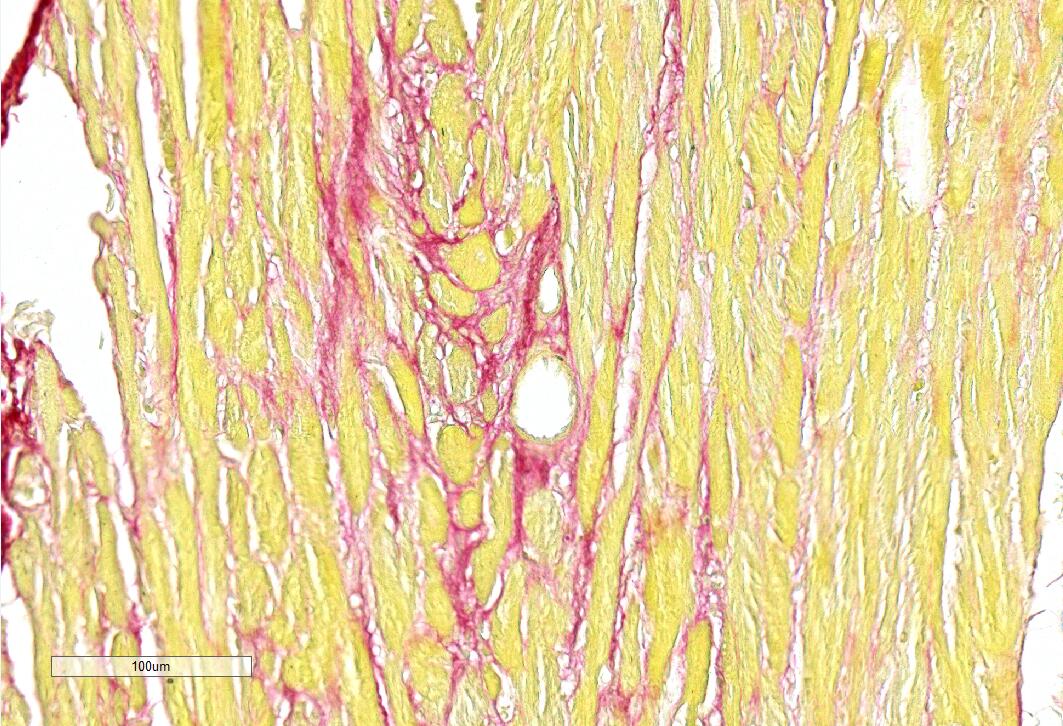

Supplement: Supplementary file 1 [file DataSheet1.ZIP › Additinal files/PSR staining/Figure 3.A PSR/AB+Veh interstitial.jpg]

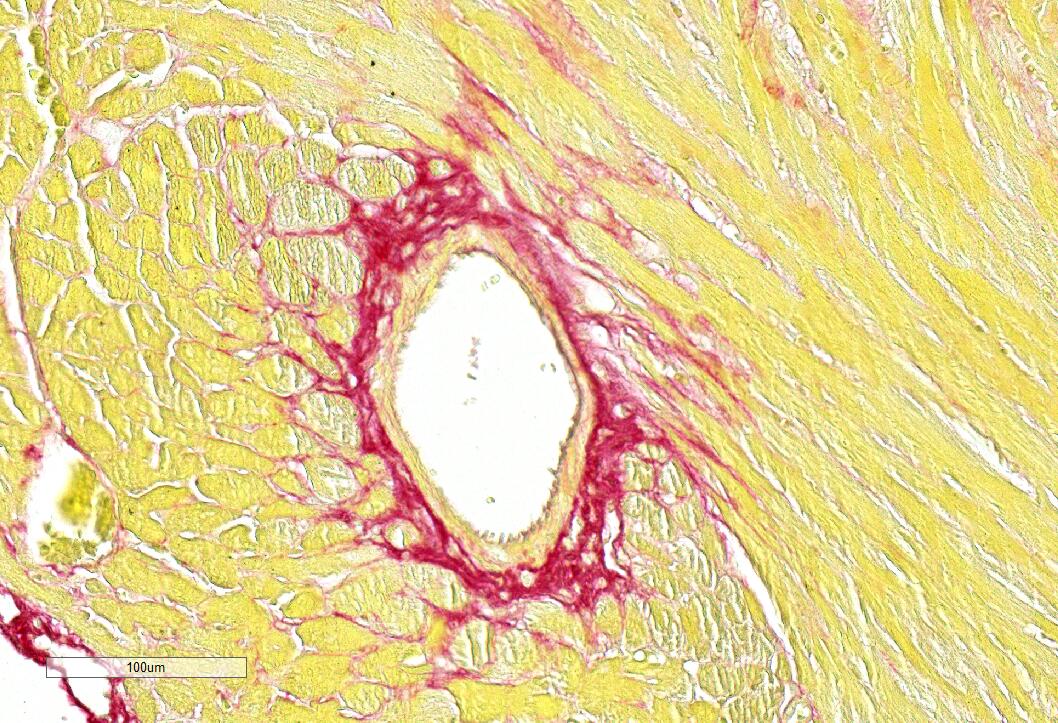

Supplement: Supplementary file 1 [file DataSheet1.ZIP › Additinal files/PSR staining/Figure 3.A PSR/AB+Veh perivascular.jpg]

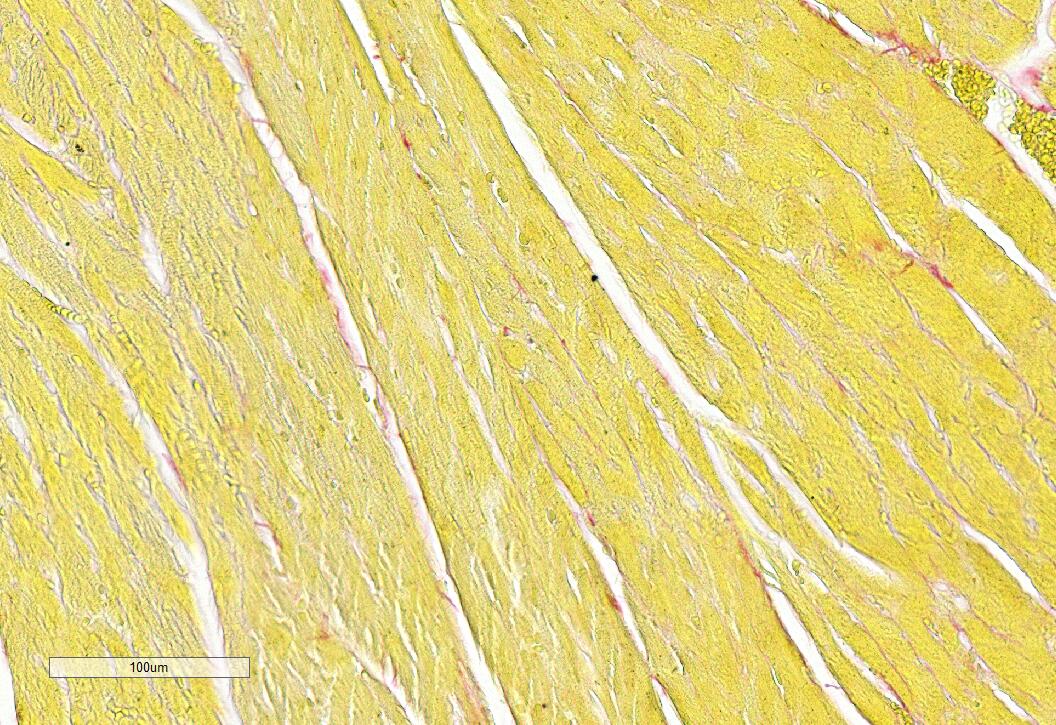

Supplement: Supplementary file 1 [file DataSheet1.ZIP › Additinal files/PSR staining/Figure 3.A PSR/Sham+LQ interstitial.jpg]

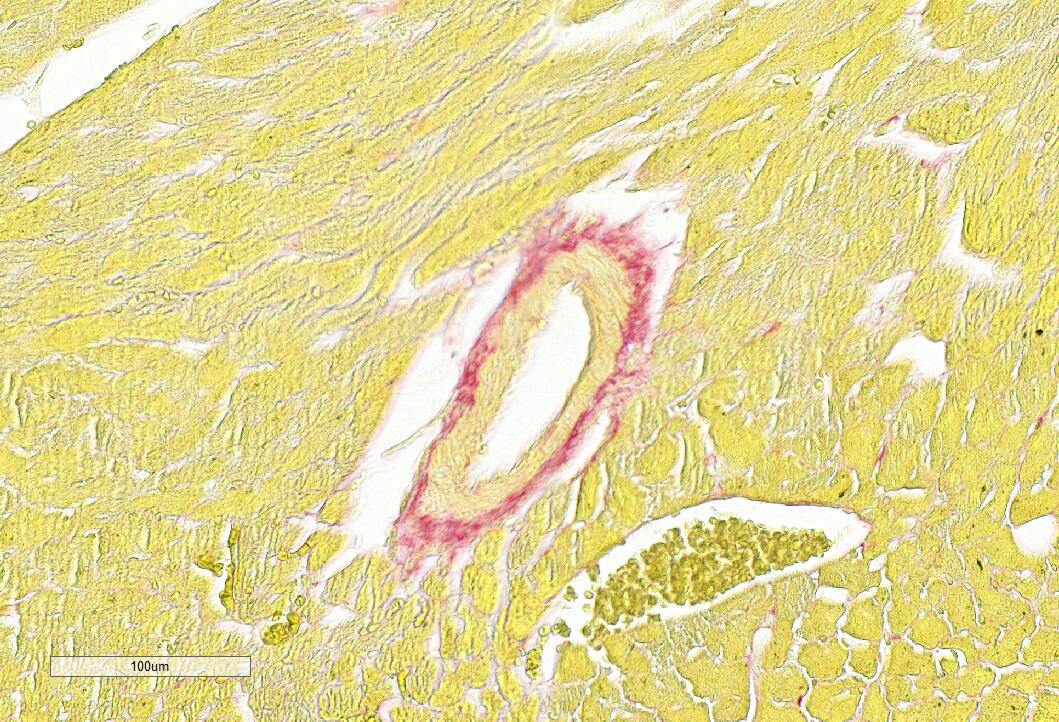

Supplement: Supplementary file 1 [file DataSheet1.ZIP › Additinal files/PSR staining/Figure 3.A PSR/Sham+LQ perivascular.jpg]

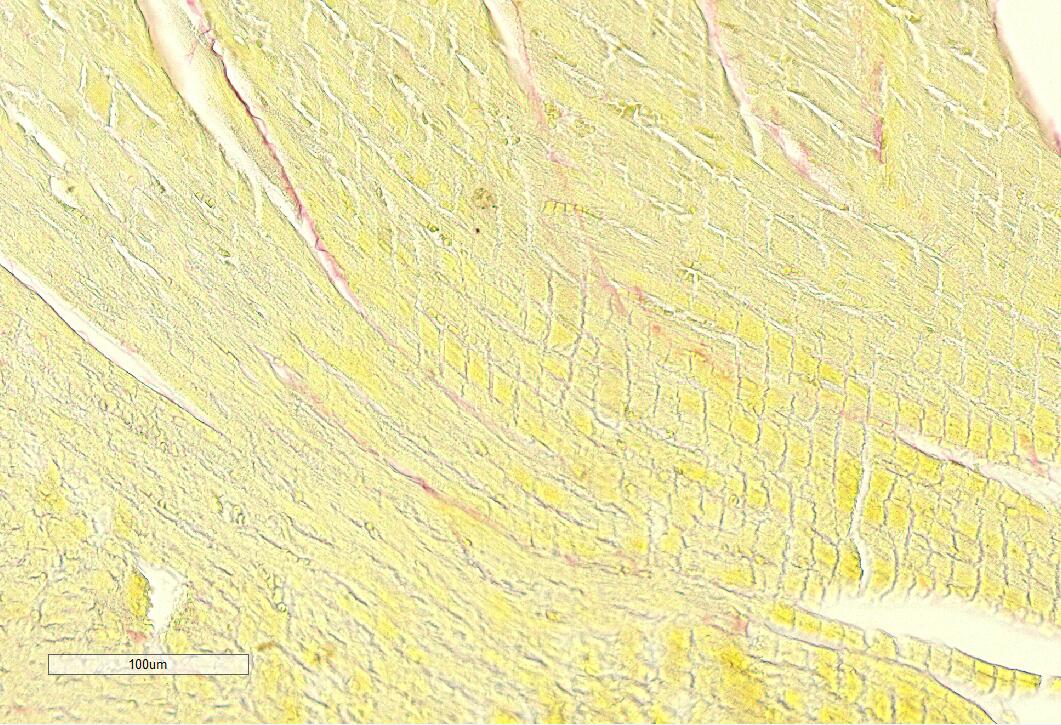

Supplement: Supplementary file 1 [file DataSheet1.ZIP › Additinal files/PSR staining/Figure 3.A PSR/Sham+Veh interstitial.jpg]

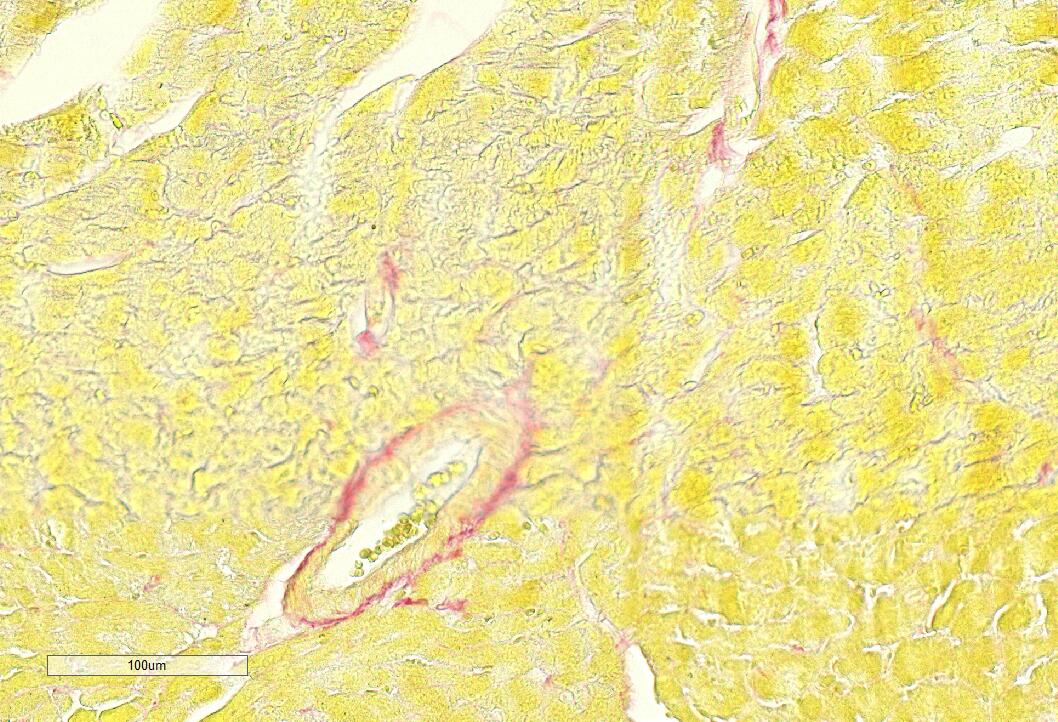

Supplement: Supplementary file 1 [file DataSheet1.ZIP › Additinal files/PSR staining/Figure 3.A PSR/Sham+Veh perivascular.jpg]

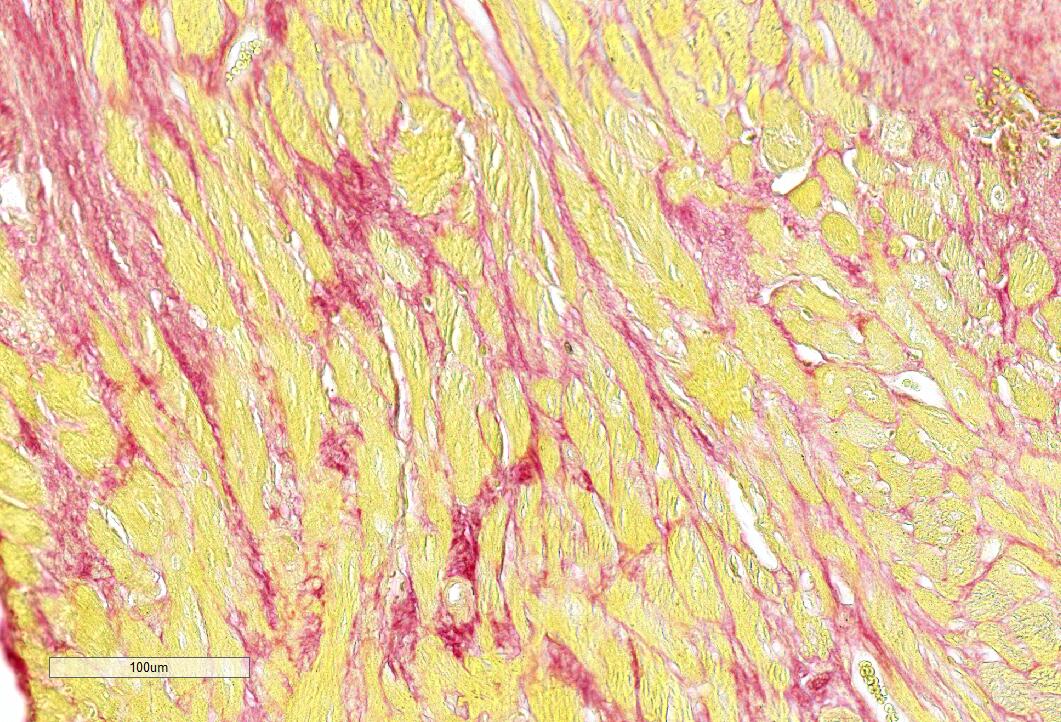

Supplement: Supplementary file 1 [file DataSheet1.ZIP › Additinal files/PSR staining/Supplementary Figure S1.I PSR/KO AB+LQ interstitial.jpg]

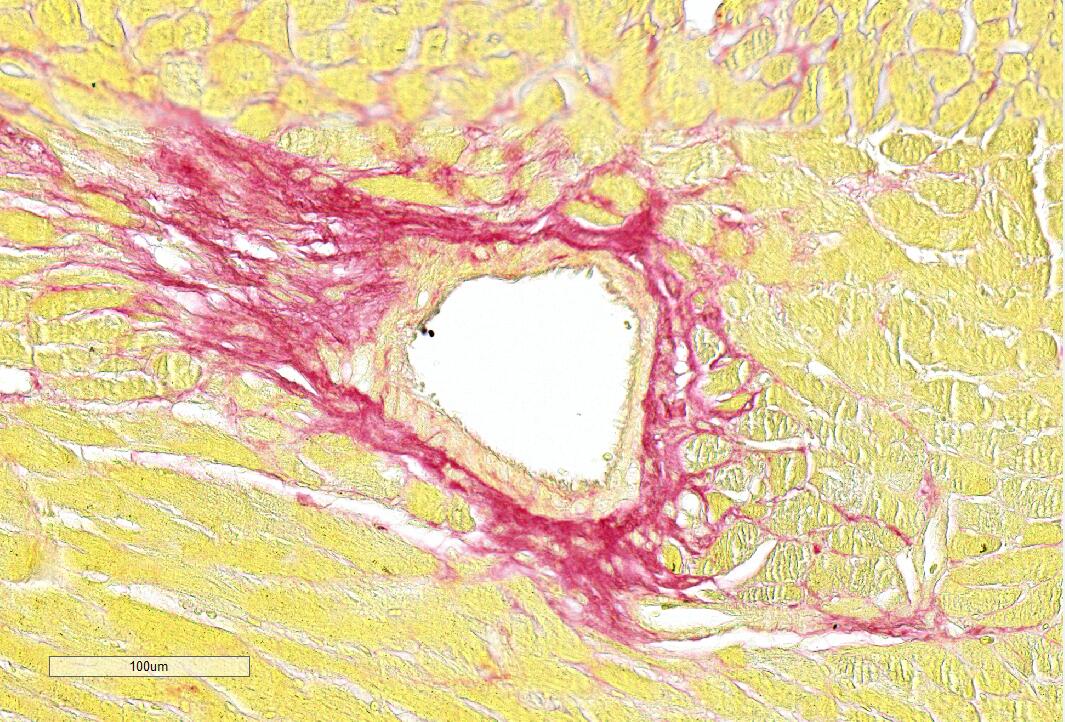

Supplement: Supplementary file 1 [file DataSheet1.ZIP › Additinal files/PSR staining/Supplementary Figure S1.I PSR/KO AB+LQ perivascular.jpg]

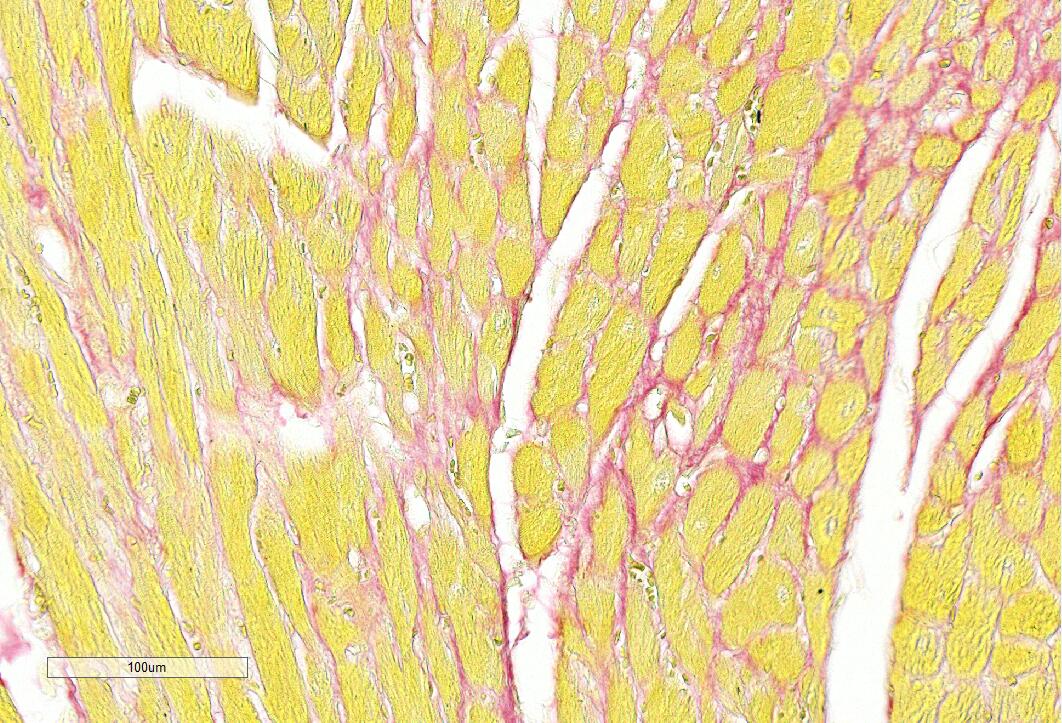

Supplement: Supplementary file 1 [file DataSheet1.ZIP › Additinal files/PSR staining/Supplementary Figure S1.I PSR/KO AB+Veh interstitial.jpg]

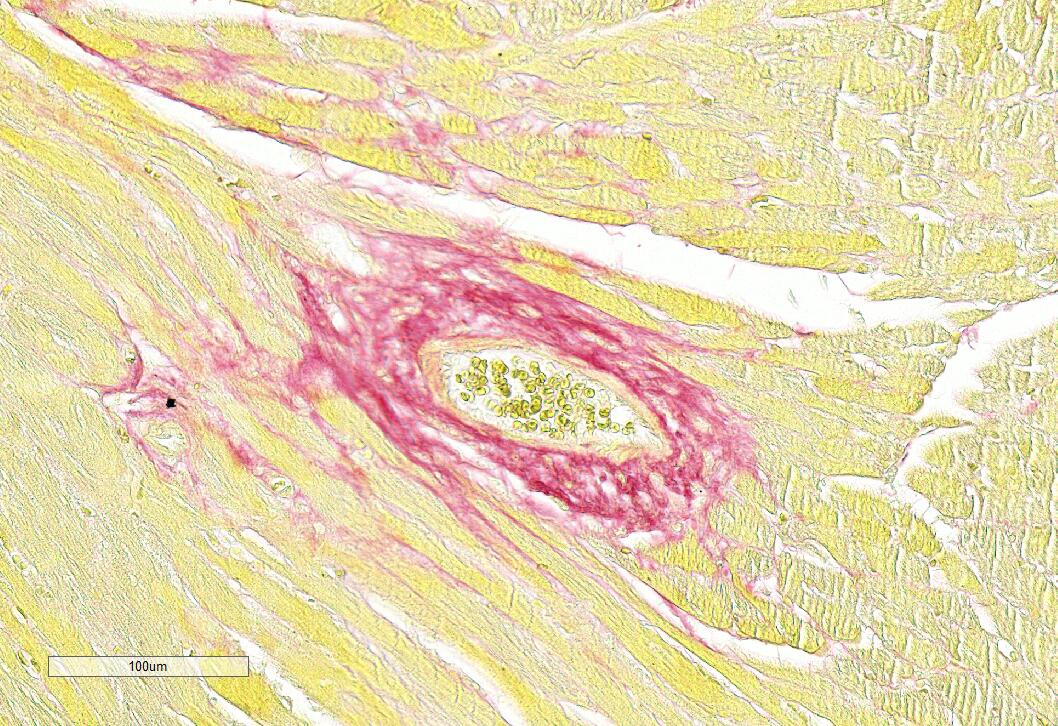

Supplement: Supplementary file 1 [file DataSheet1.ZIP › Additinal files/PSR staining/Supplementary Figure S1.I PSR/KO AB+Veh perivascular.jpg]

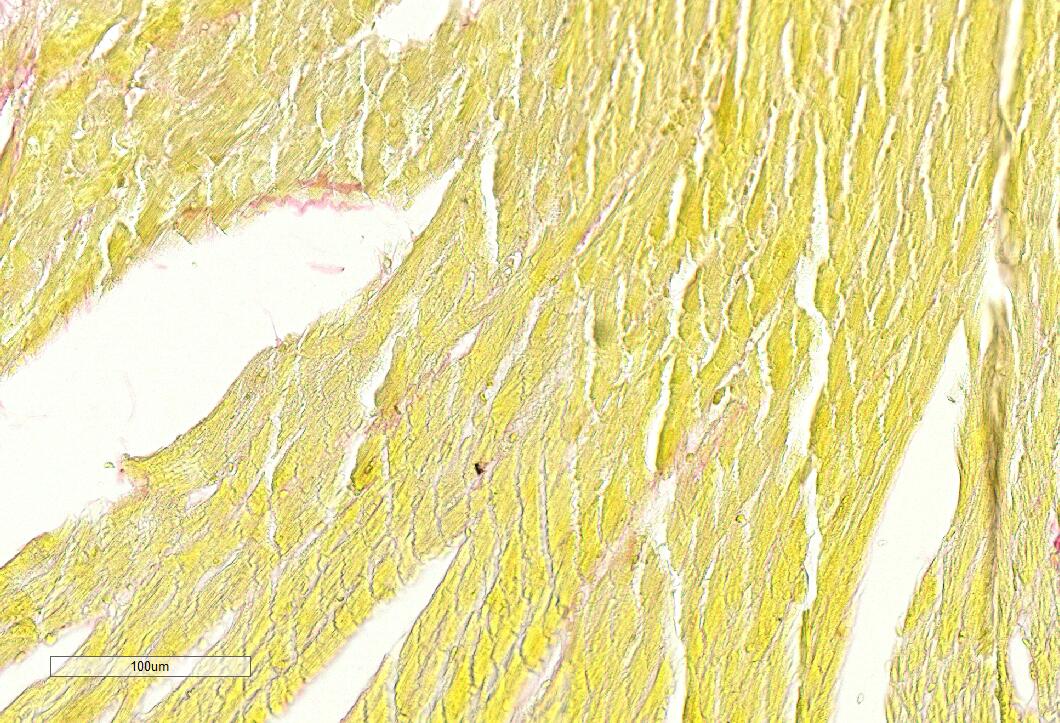

Supplement: Supplementary file 1 [file DataSheet1.ZIP › Additinal files/PSR staining/Supplementary Figure S1.I PSR/KO Sham+Veh interstitial.jpg]

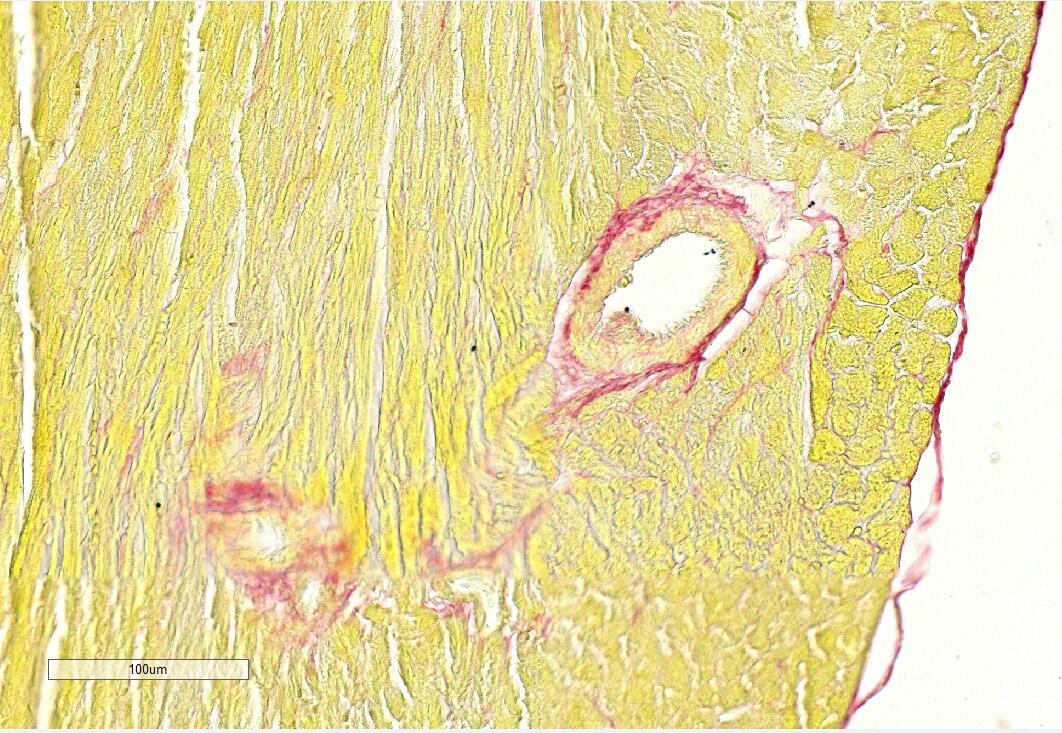

Supplement: Supplementary file 1 [file DataSheet1.ZIP › Additinal files/PSR staining/Supplementary Figure S1.I PSR/KO Sham+Veh perivascular.jpg]

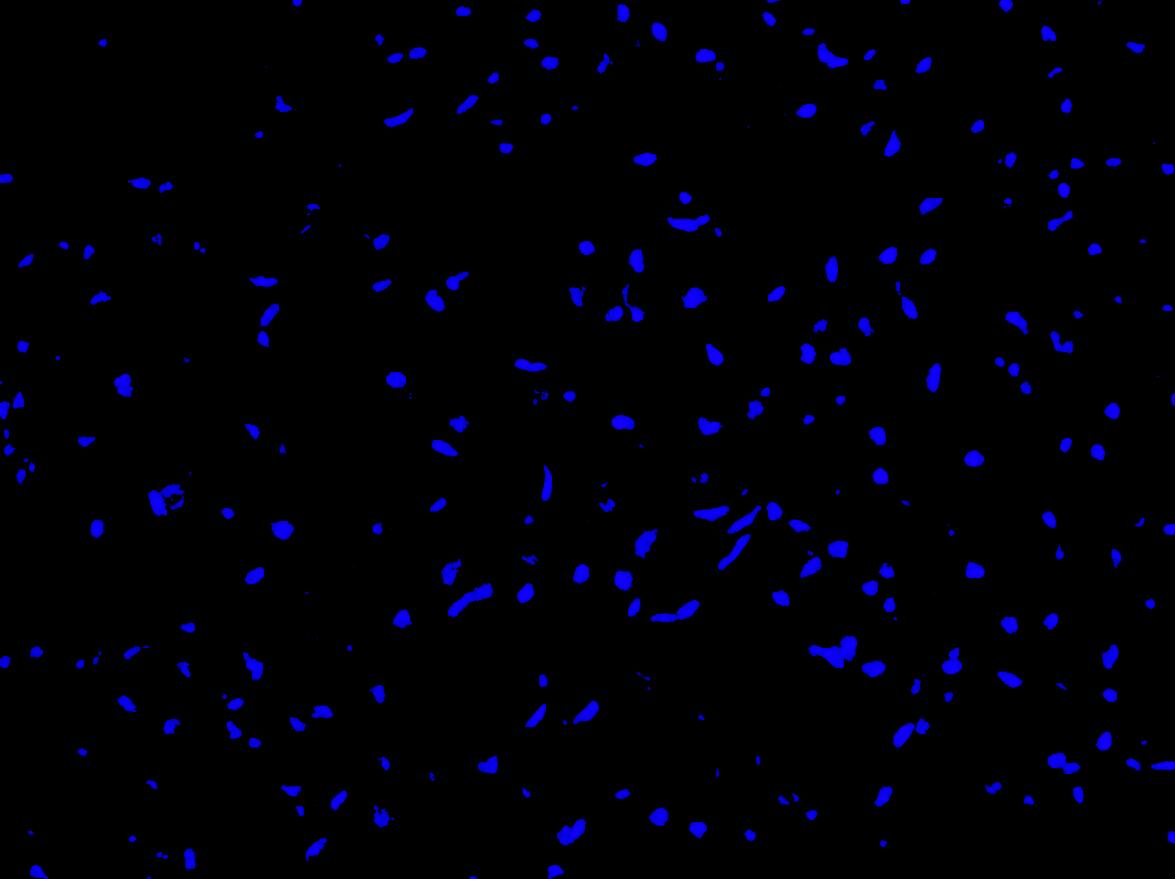

Supplement: Supplementary file 1 [file DataSheet1.ZIP › Additinal files/TUNEL staining/AB+LQ blue.jpg]

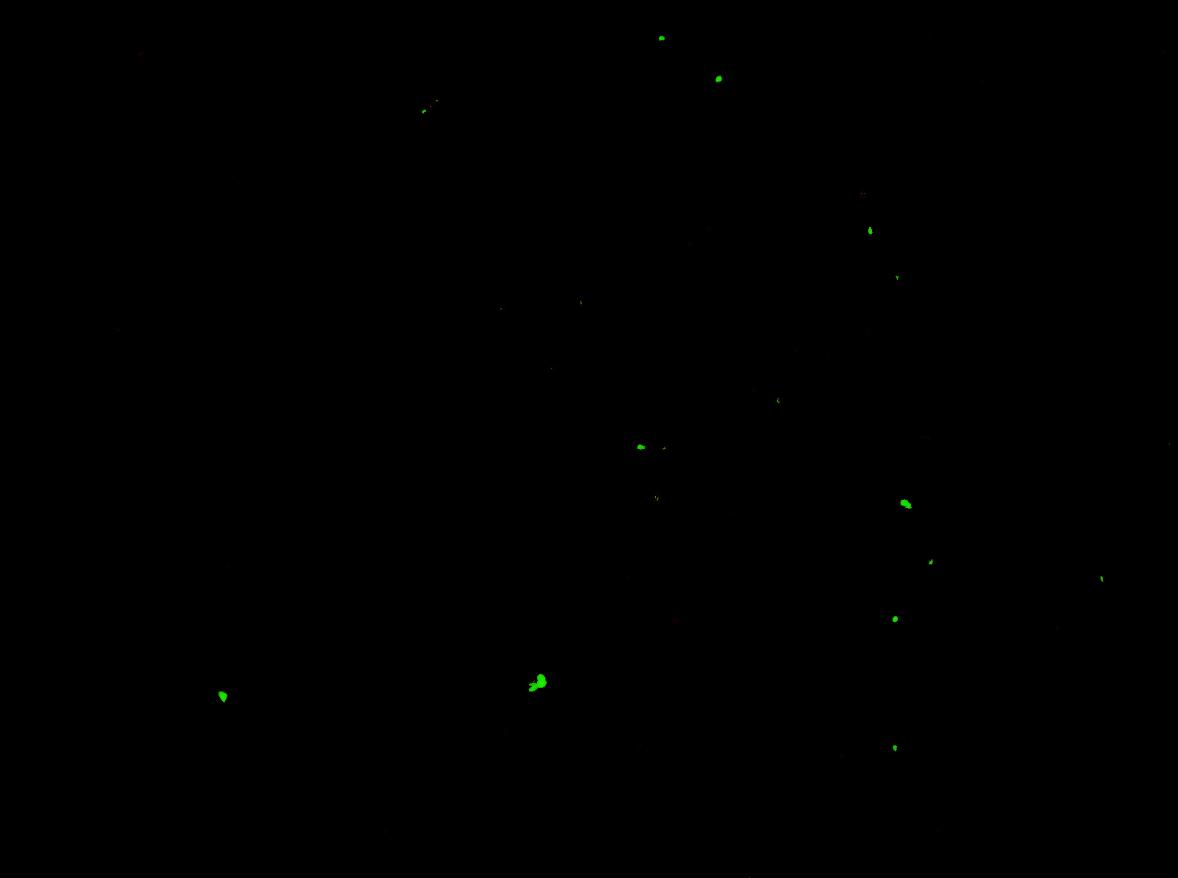

Supplement: Supplementary file 1 [file DataSheet1.ZIP › Additinal files/TUNEL staining/AB+LQ green.jpg]

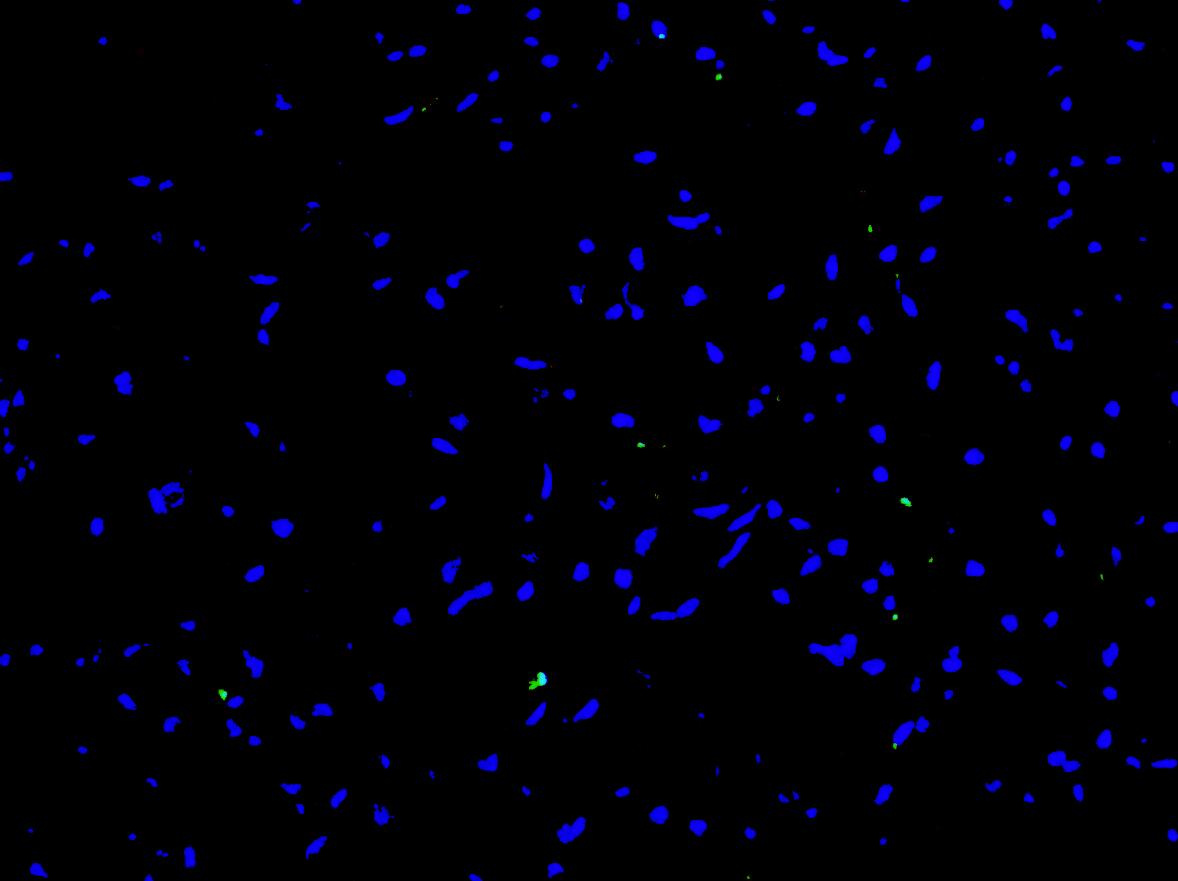

Supplement: Supplementary file 1 [file DataSheet1.ZIP › Additinal files/TUNEL staining/AB+LQ merge.jpg]

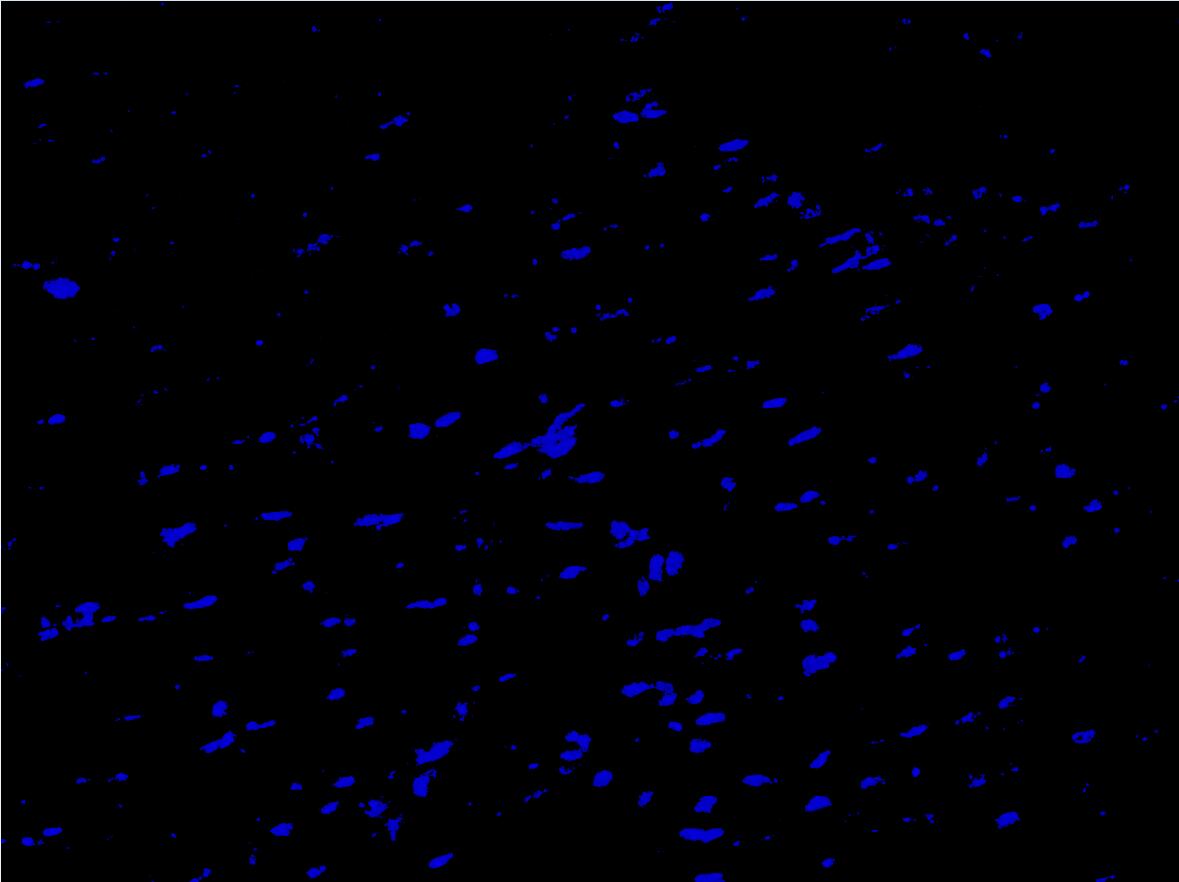

Supplement: Supplementary file 1 [file DataSheet1.ZIP › Additinal files/TUNEL staining/AB+Veh blue.jpg]

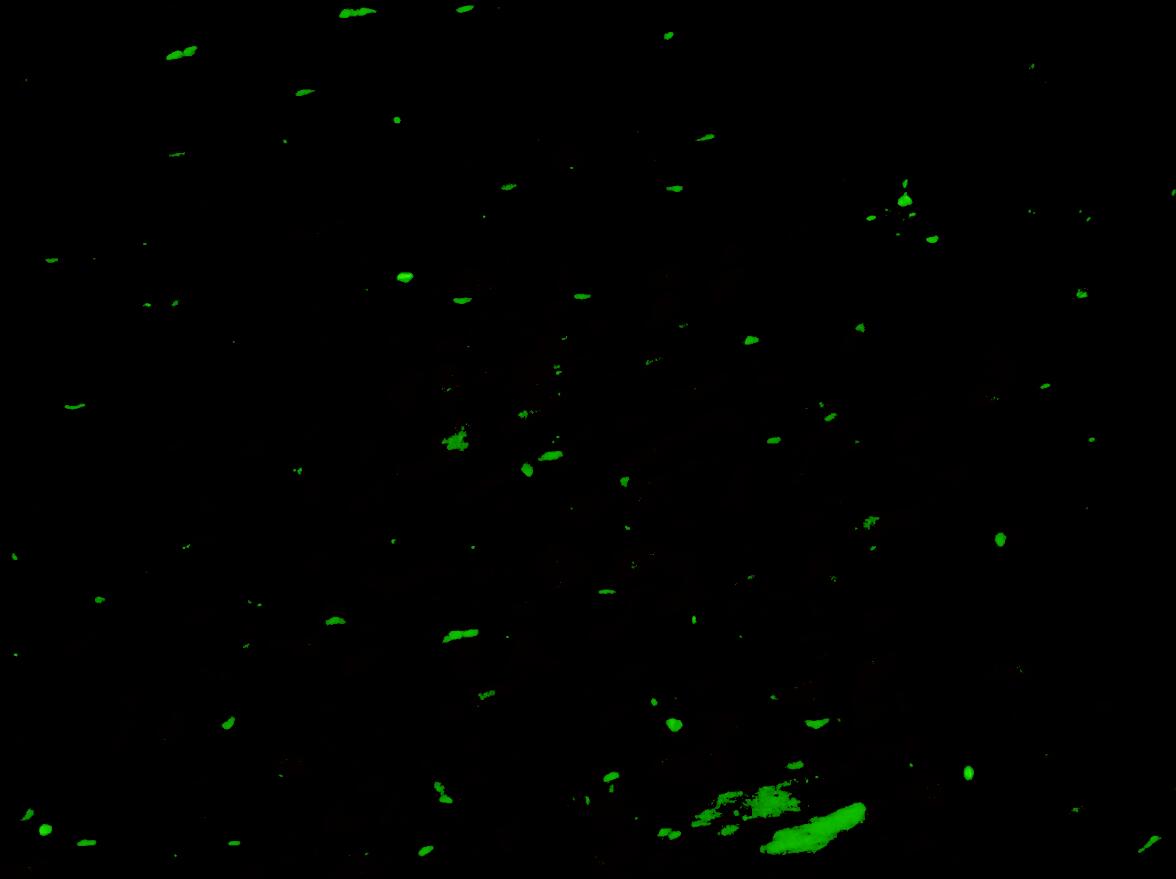

Supplement: Supplementary file 1 [file DataSheet1.ZIP › Additinal files/TUNEL staining/AB+Veh green.jpg]

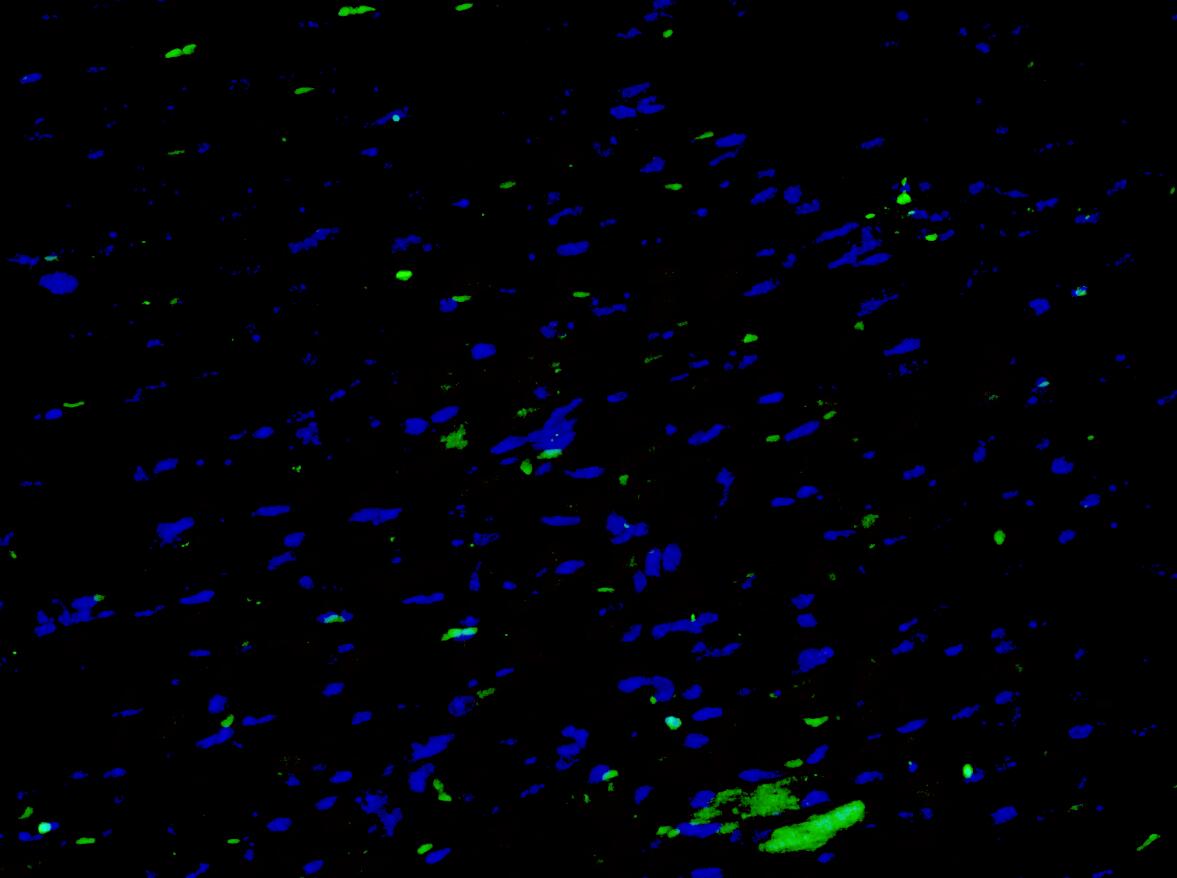

Supplement: Supplementary file 1 [file DataSheet1.ZIP › Additinal files/TUNEL staining/AB+Veh merge.jpg]

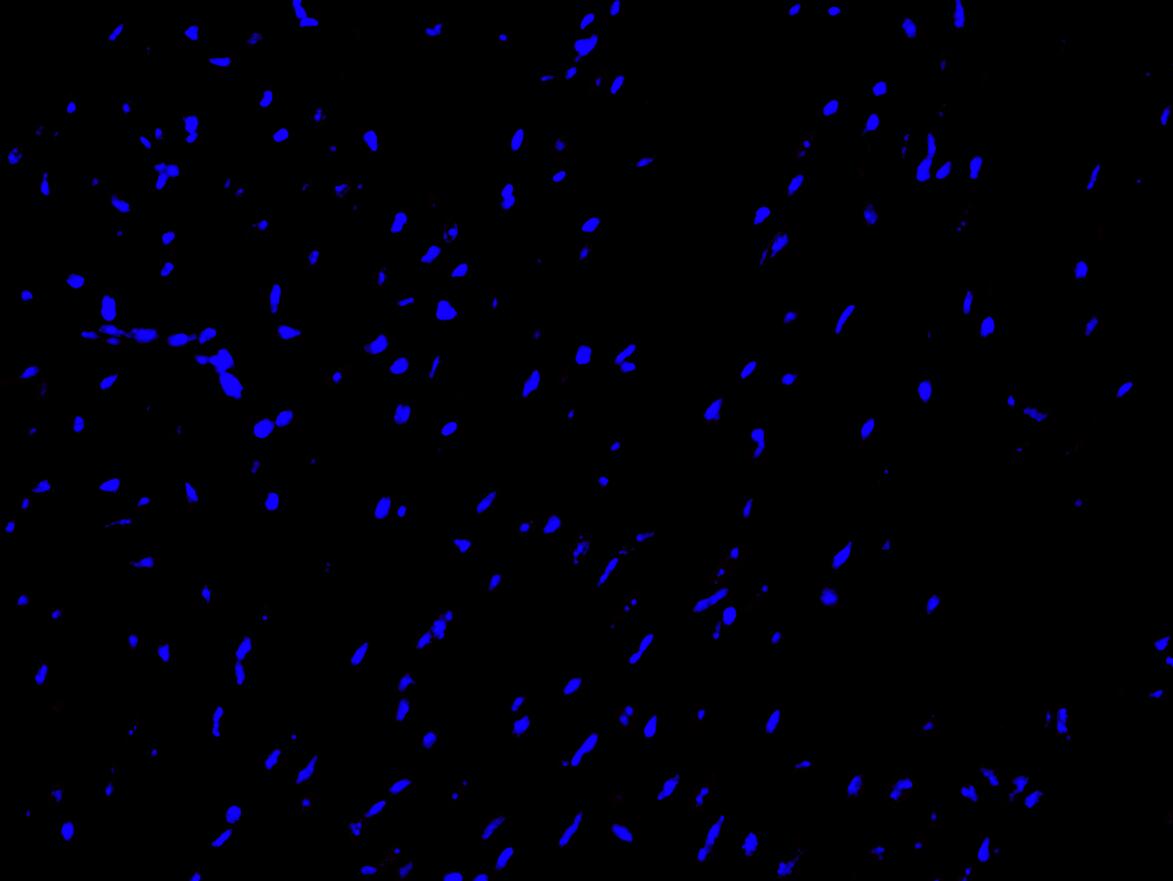

Supplement: Supplementary file 1 [file DataSheet1.ZIP › Additinal files/TUNEL staining/Sham+LQ blue.jpg]

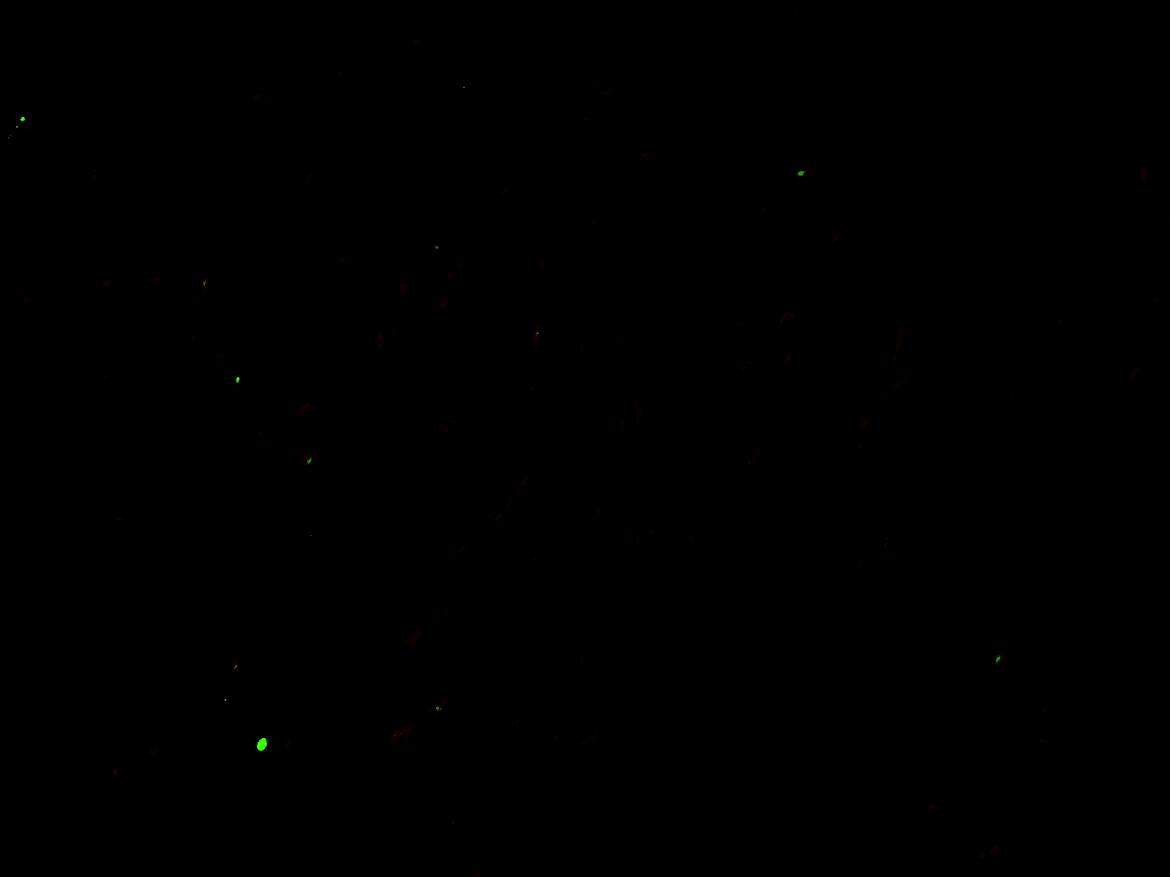

Supplement: Supplementary file 1 [file DataSheet1.ZIP › Additinal files/TUNEL staining/Sham+LQ green.jpg]

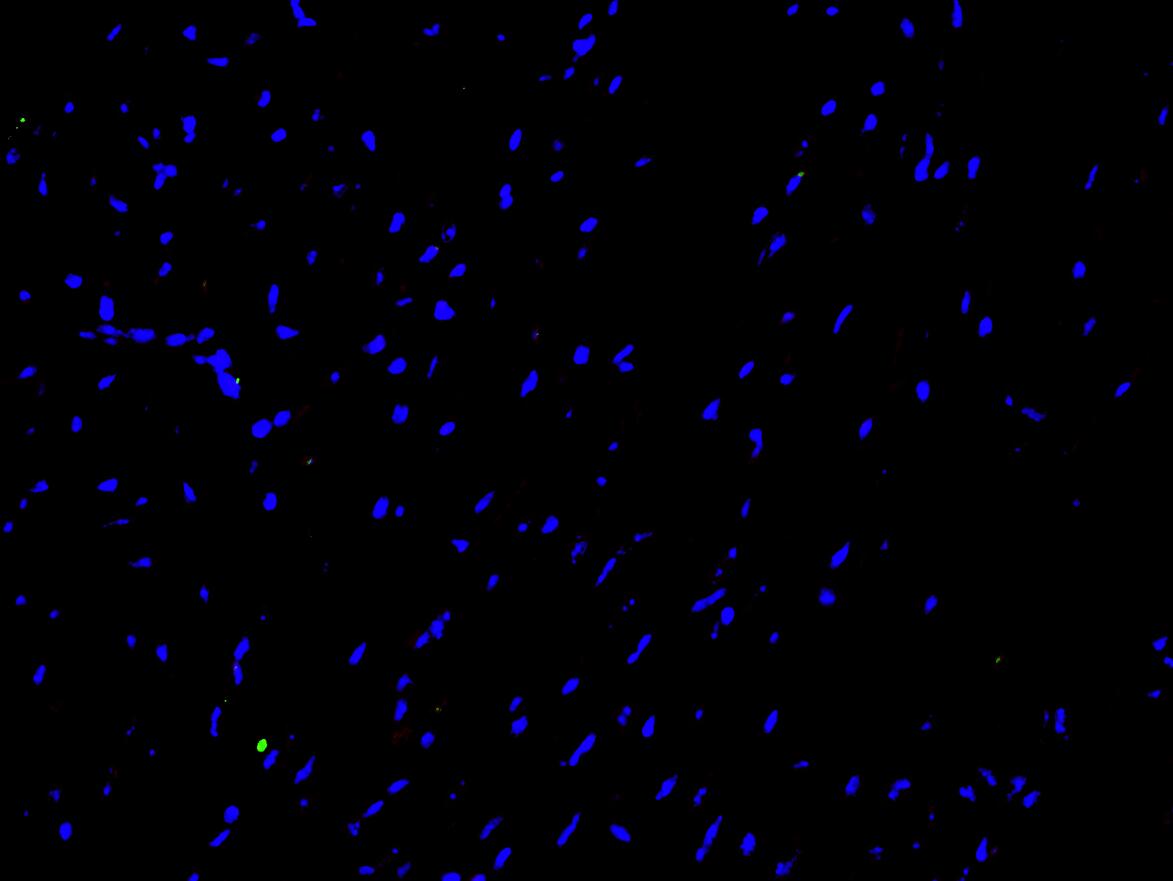

Supplement: Supplementary file 1 [file DataSheet1.ZIP › Additinal files/TUNEL staining/Sham+LQ merge.jpg]

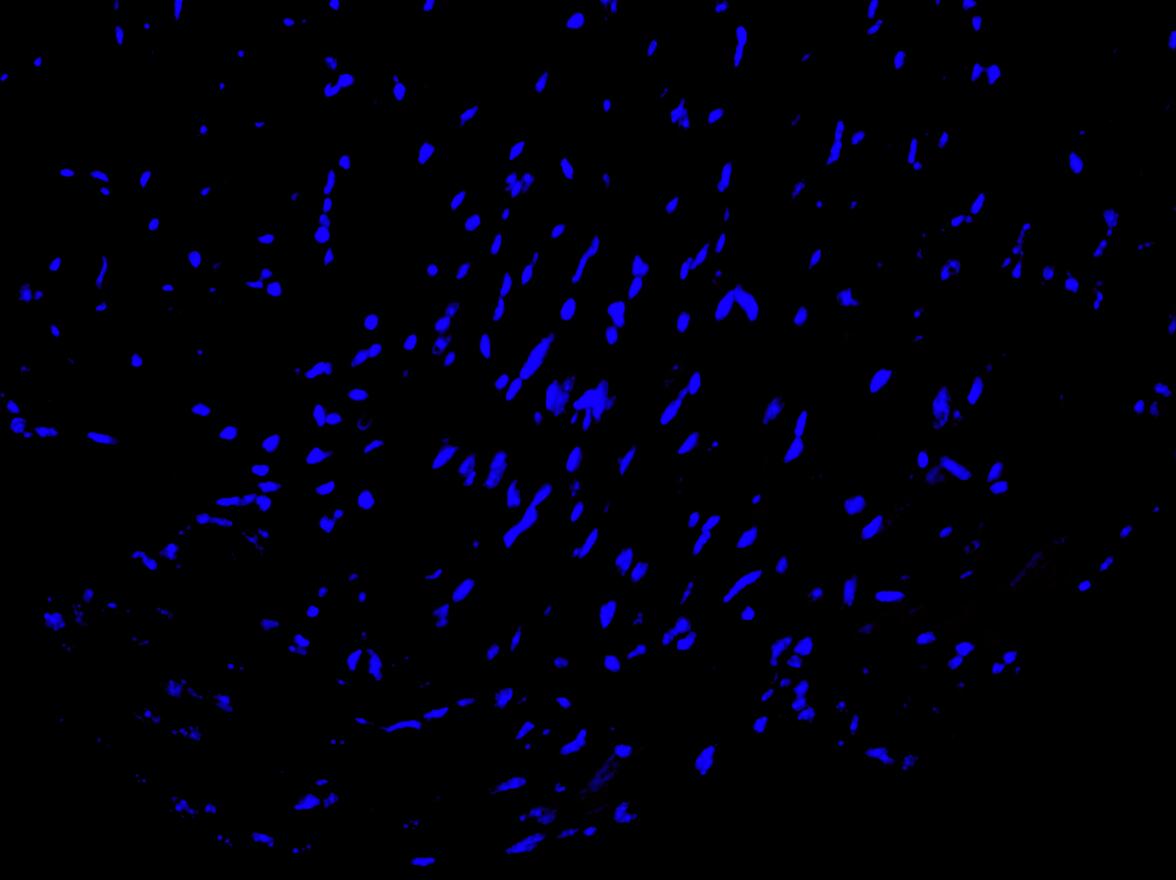

Supplement: Supplementary file 1 [file DataSheet1.ZIP › Additinal files/TUNEL staining/Sham+Veh blue.jpg]

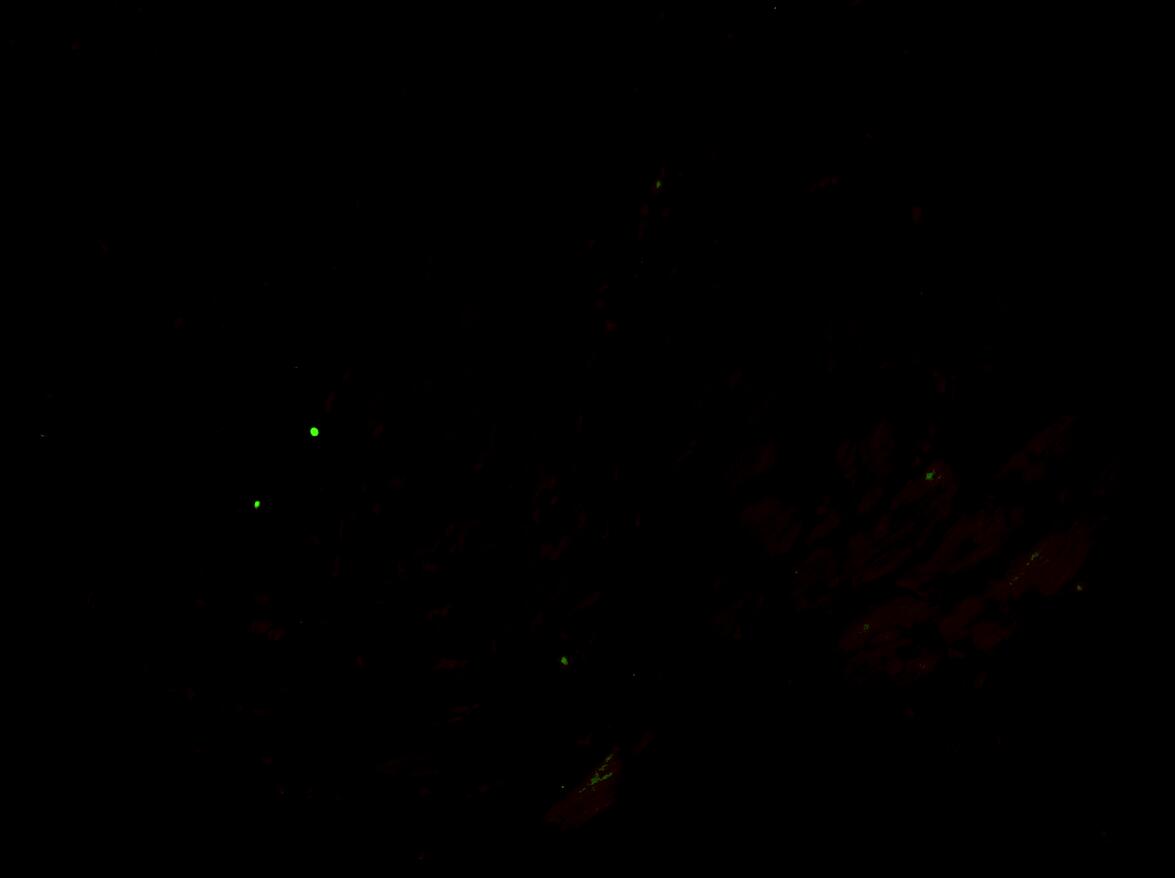

Supplement: Supplementary file 1 [file DataSheet1.ZIP › Additinal files/TUNEL staining/Sham+Veh green.jpg]

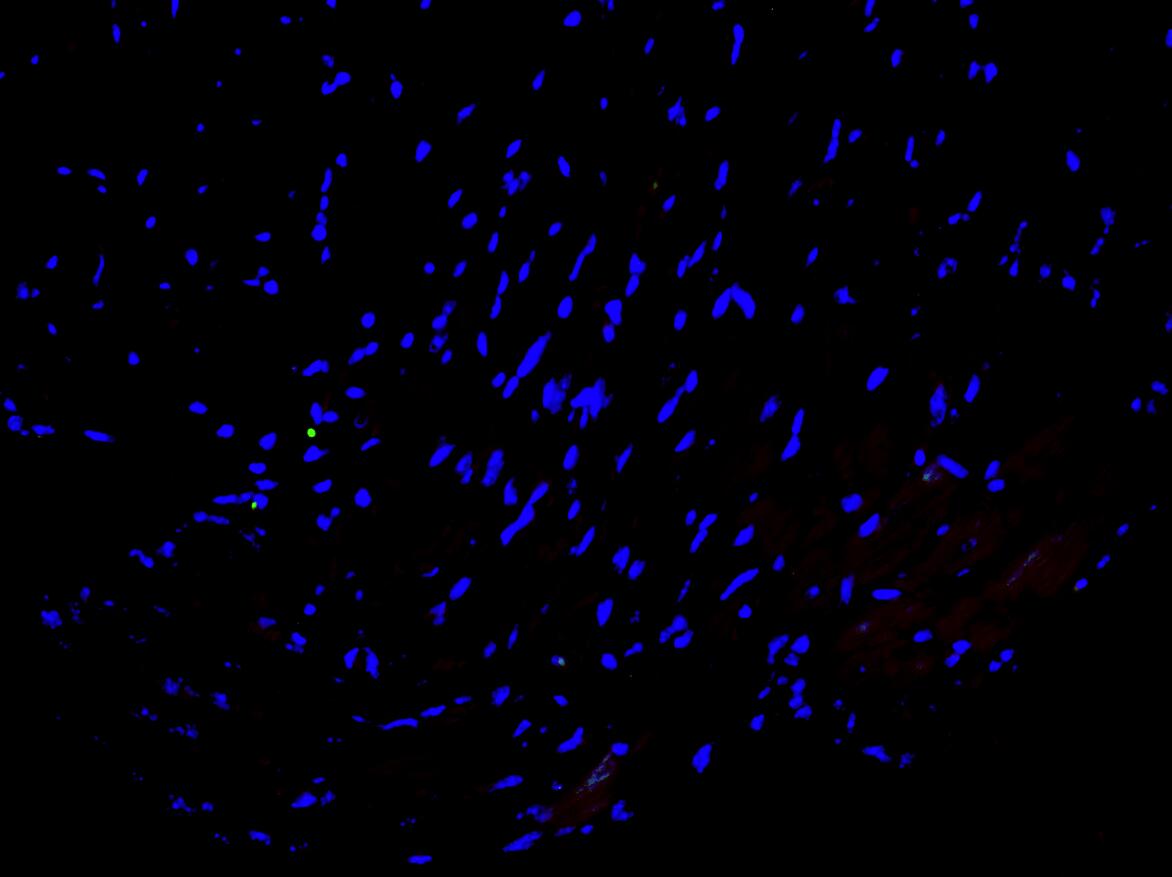

Supplement: Supplementary file 1 [file DataSheet1.ZIP › Additinal files/TUNEL staining/Sham+Veh merge.jpg]

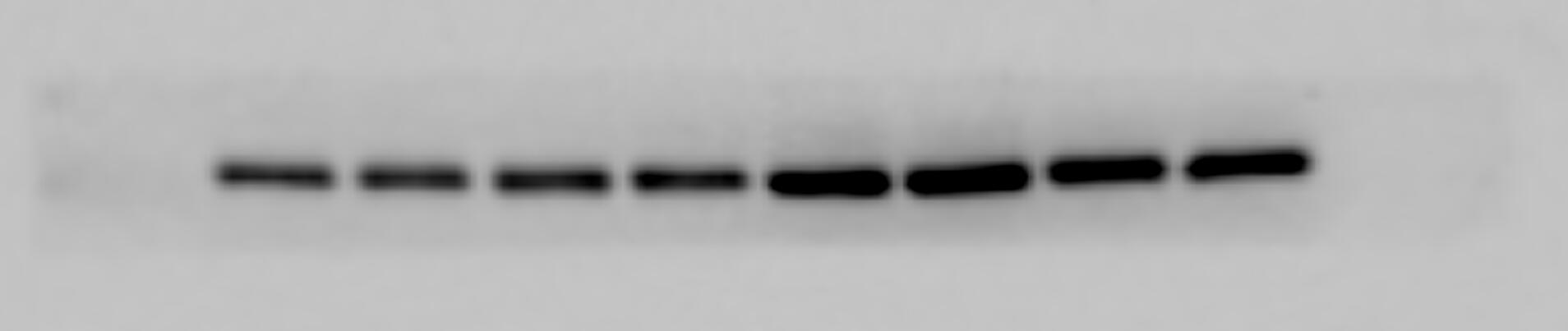

Supplement: Supplementary file 1 [file DataSheet1.ZIP › Additinal files/Western blots/Figure 4.C Western blots/Figure 4.C Bax.jpg]

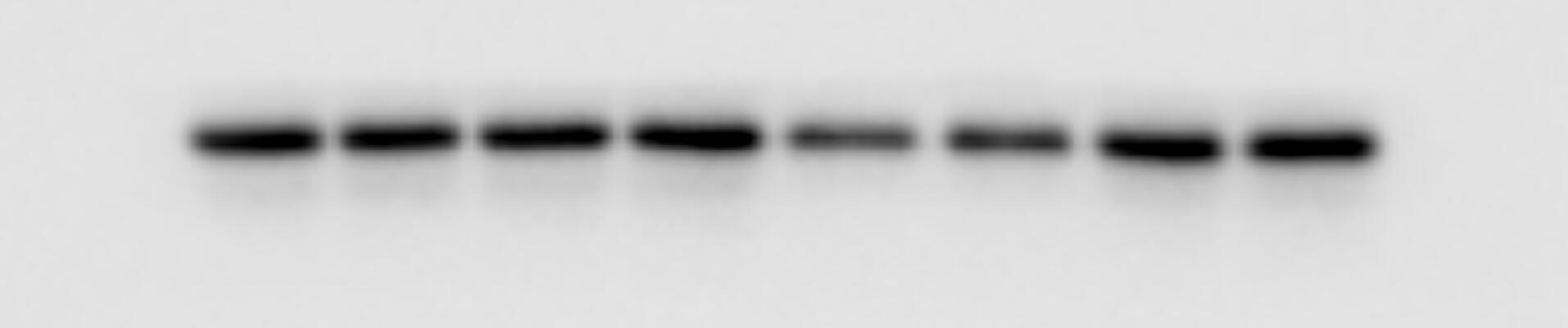

Supplement: Supplementary file 1 [file DataSheet1.ZIP › Additinal files/Western blots/Figure 4.C Western blots/Figure 4.C Bcl-2.jpg]

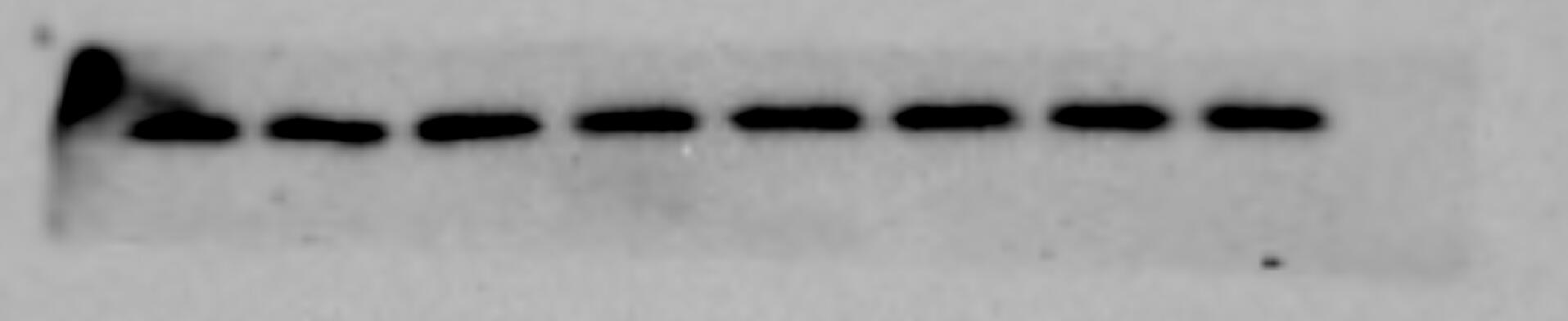

Supplement: Supplementary file 1 [file DataSheet1.ZIP › Additinal files/Western blots/Figure 4.C Western blots/Figure 4.C GAPDH.jpg]

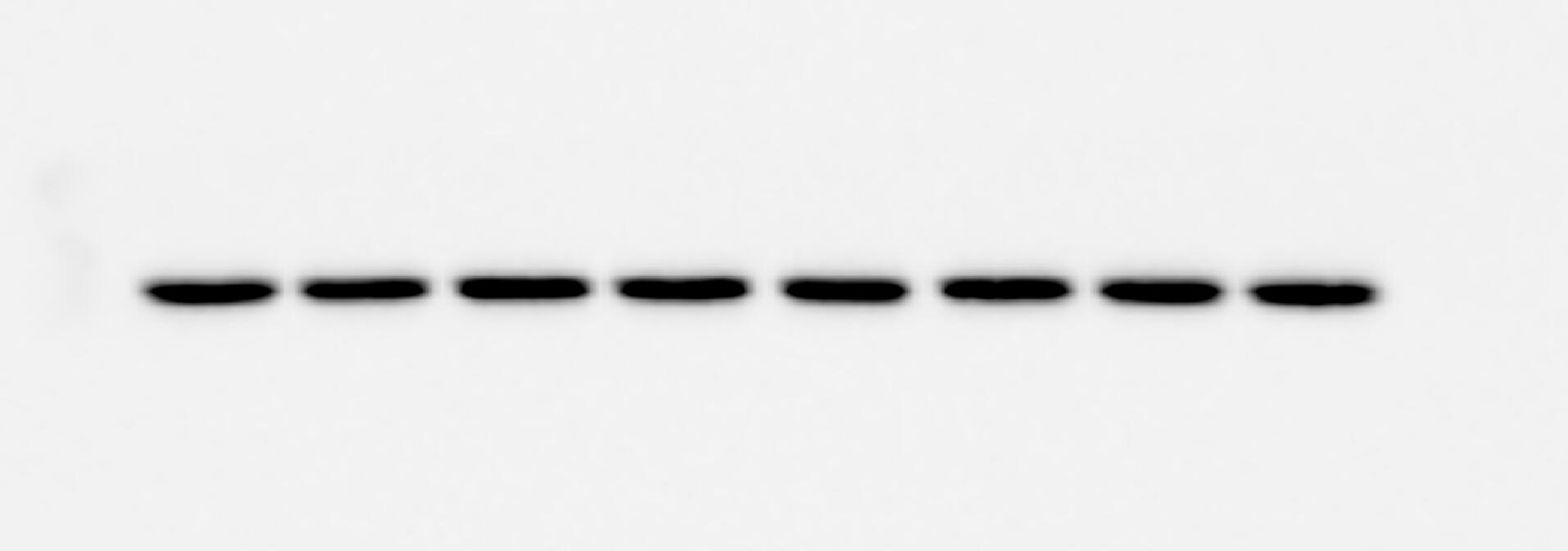

Supplement: Supplementary file 1 [file DataSheet1.ZIP › Additinal files/Western blots/Figure 5.A Western blots/Figure 5.A GAPDH.jpg]

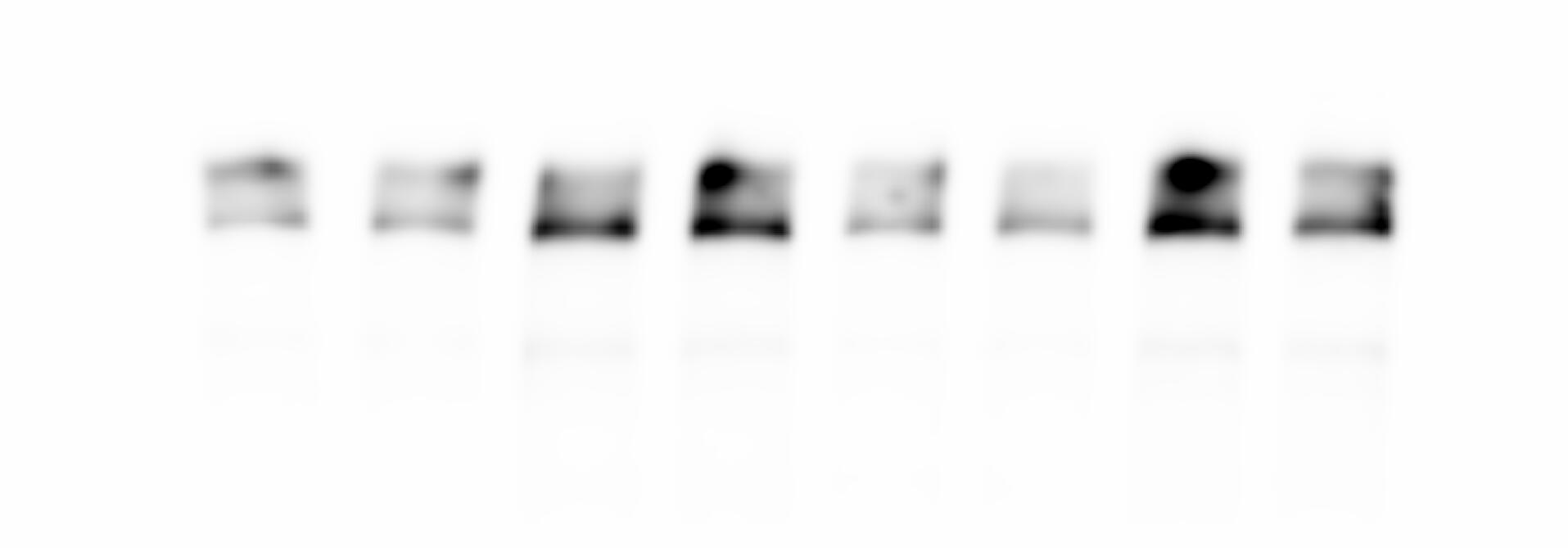

Supplement: Supplementary file 1 [file DataSheet1.ZIP › Additinal files/Western blots/Figure 5.A Western blots/Figure 5.A p-ACC.jpg]

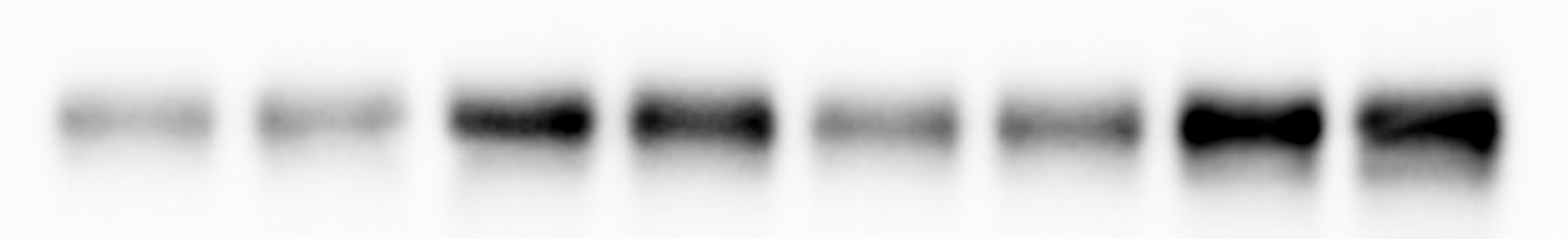

Supplement: Supplementary file 1 [file DataSheet1.ZIP › Additinal files/Western blots/Figure 5.A Western blots/Figure 5.A p-AMPKa2.jpg]

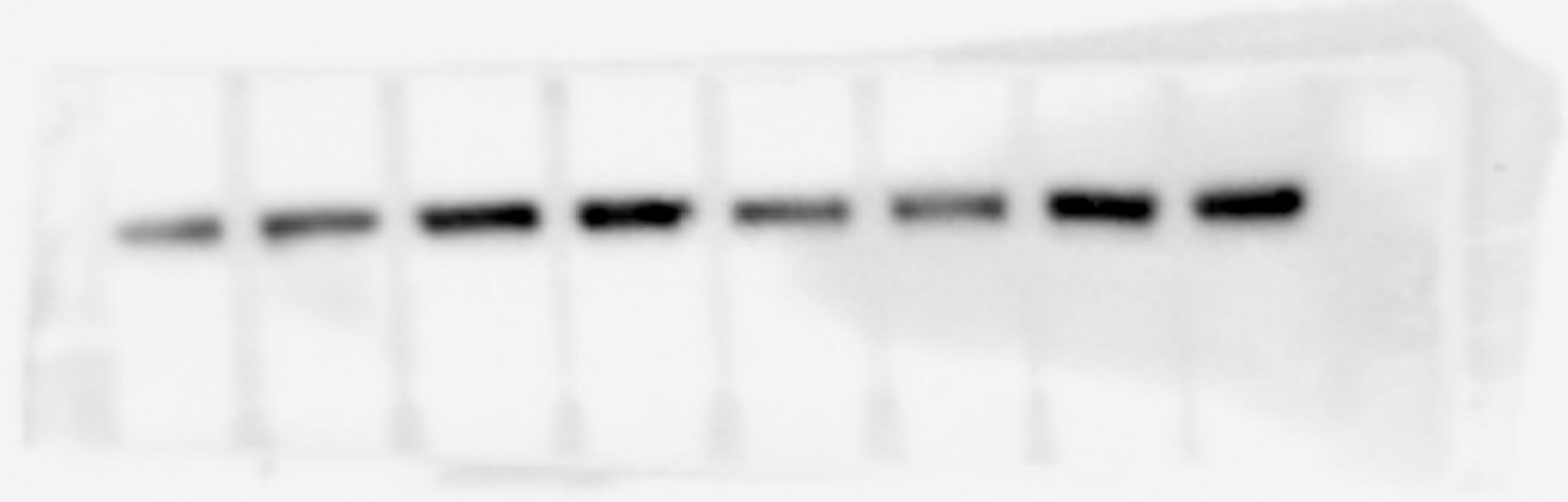

Supplement: Supplementary file 1 [file DataSheet1.ZIP › Additinal files/Western blots/Figure 5.A Western blots/Figure 5.A p-LKB1.jpg]

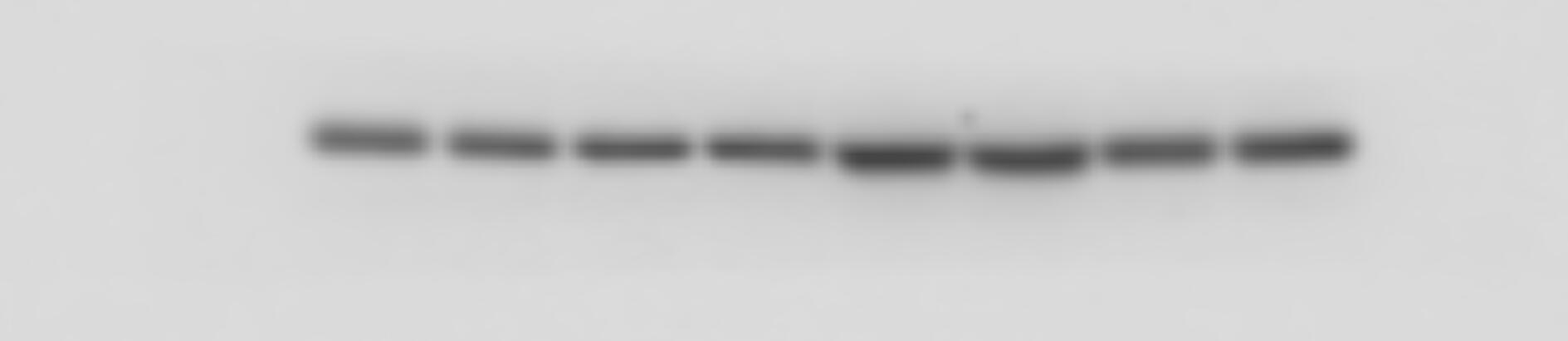

Supplement: Supplementary file 1 [file DataSheet1.ZIP › Additinal files/Western blots/Figure 5.A Western blots/Figure 5.A p-mTORC1.jpg]

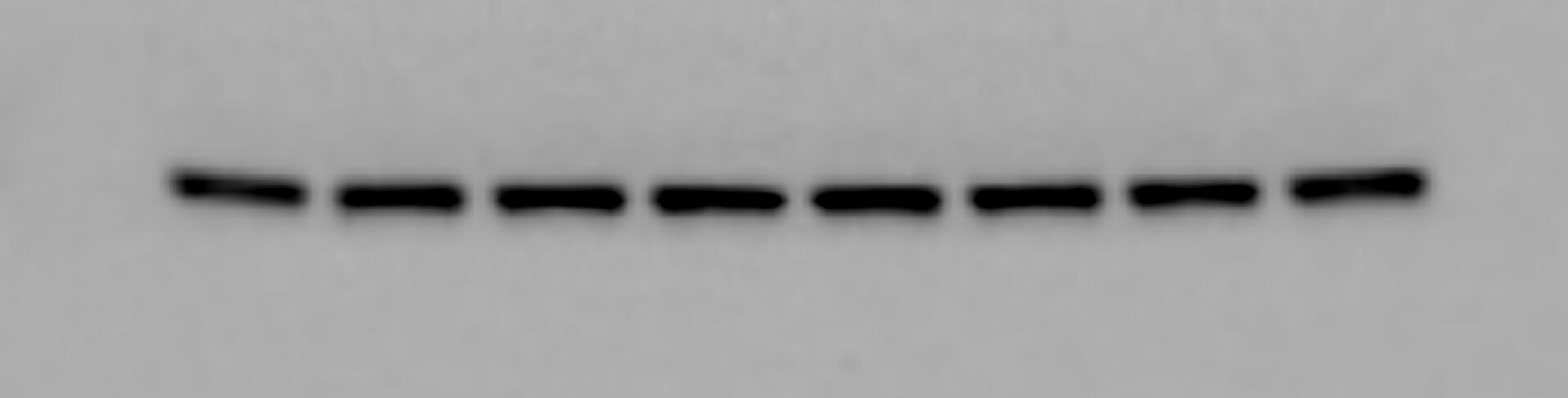

Supplement: Supplementary file 1 [file DataSheet1.ZIP › Additinal files/Western blots/Figure 5.A Western blots/Figure 5.A T-ACC.jpg]

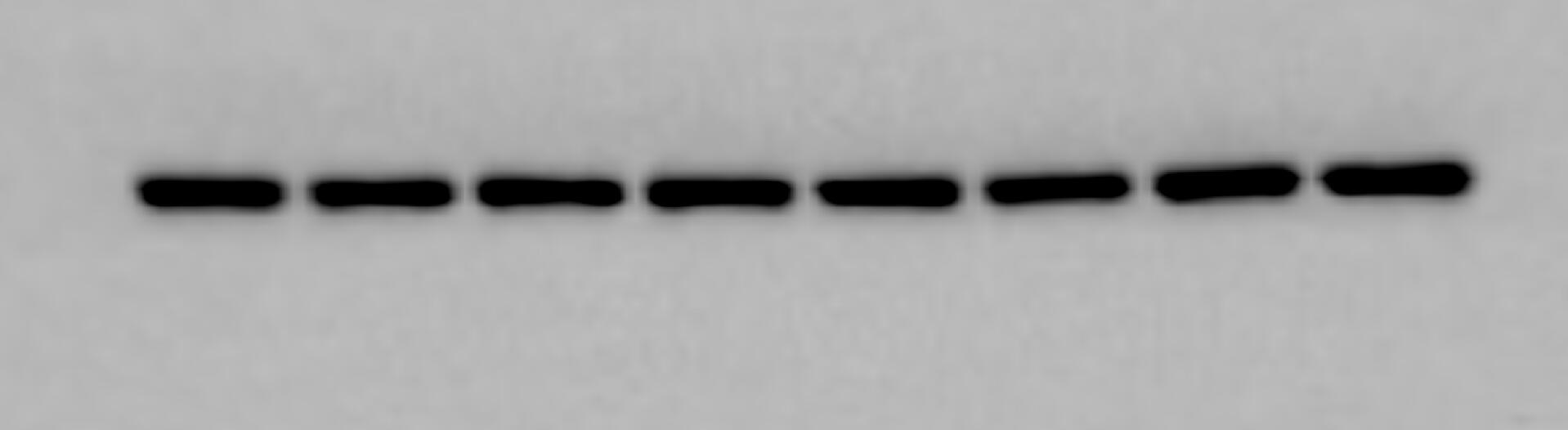

Supplement: Supplementary file 1 [file DataSheet1.ZIP › Additinal files/Western blots/Figure 5.A Western blots/Figure 5.A T-AMPKa2.jpg]

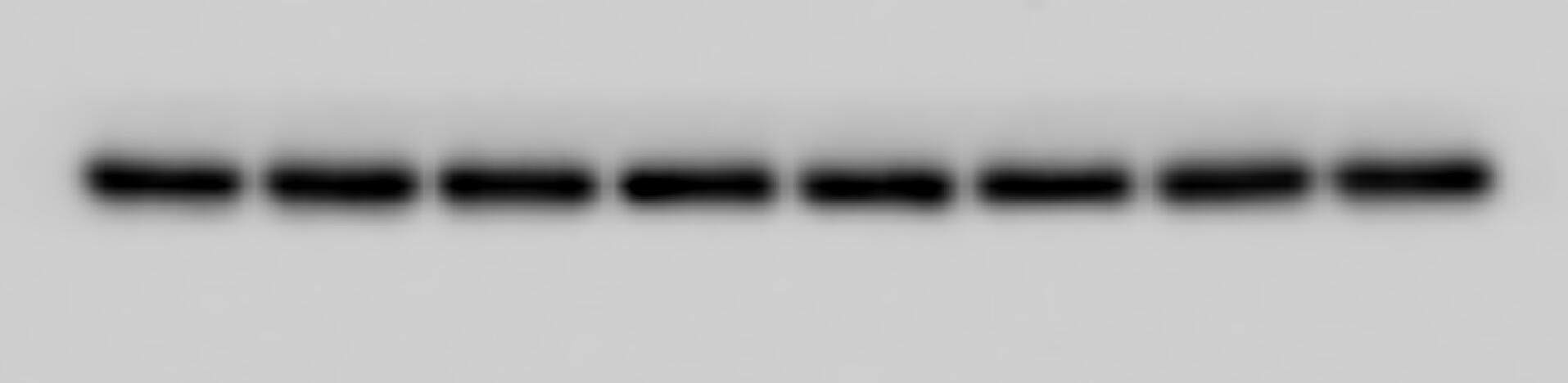

Supplement: Supplementary file 1 [file DataSheet1.ZIP › Additinal files/Western blots/Figure 5.A Western blots/Figure 5.A T-LKB1.jpg]

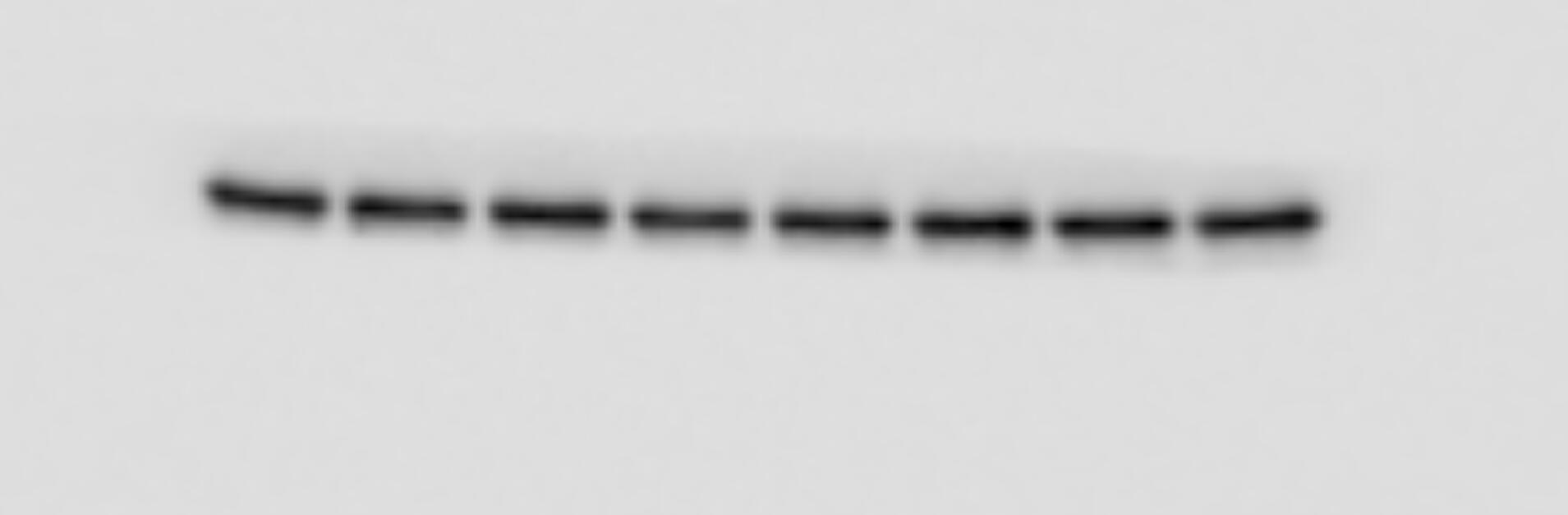

Supplement: Supplementary file 1 [file DataSheet1.ZIP › Additinal files/Western blots/Figure 5.A Western blots/Figure 5.A T-mTORC1.jpg]

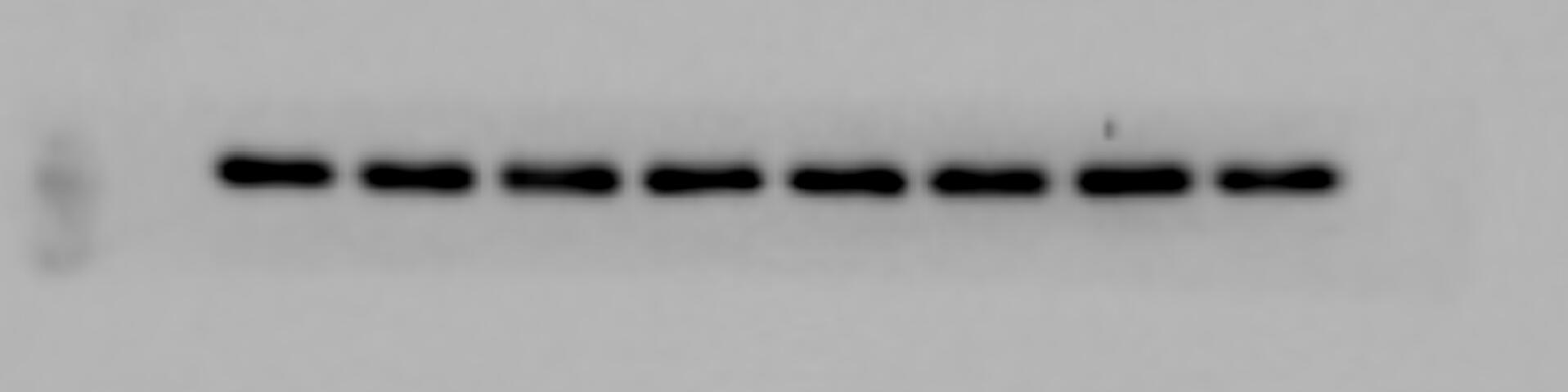

Supplement: Supplementary file 1 [file DataSheet1.ZIP › Additinal files/Western blots/Figure 6.G Western blots/Figure 6.G GAPDH.jpg]

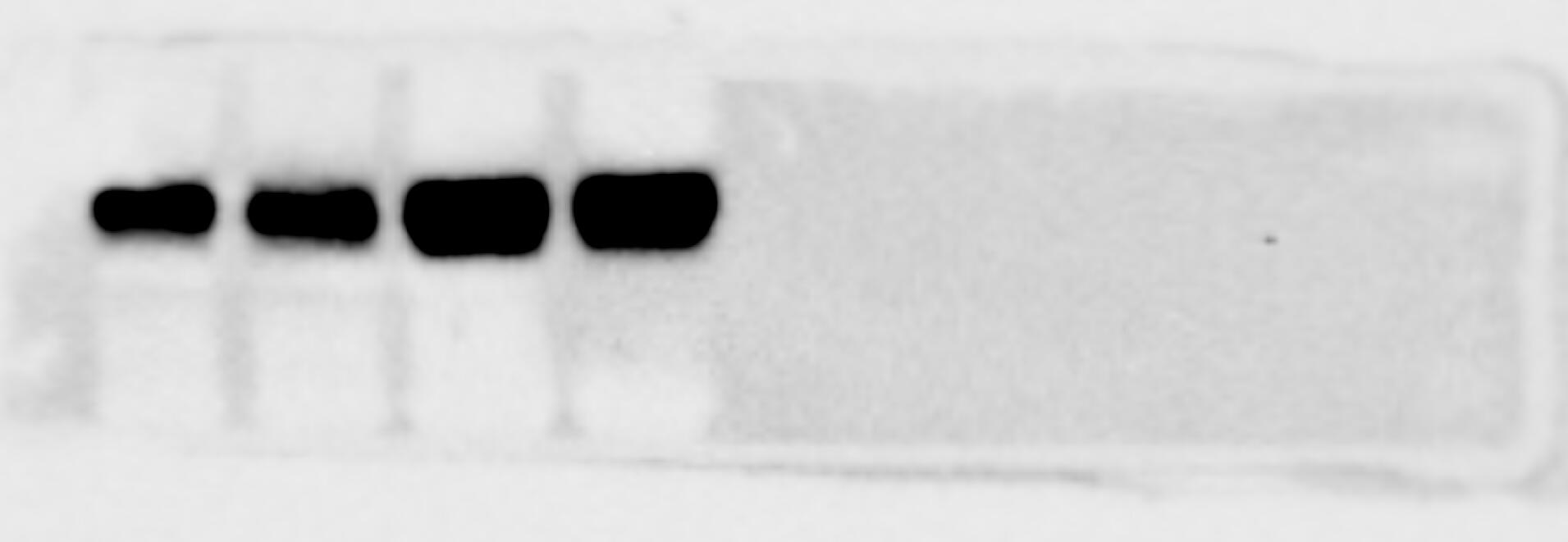

Supplement: Supplementary file 1 [file DataSheet1.ZIP › Additinal files/Western blots/Figure 6.G Western blots/Figure 6.G p-AMPKa2.jpg]

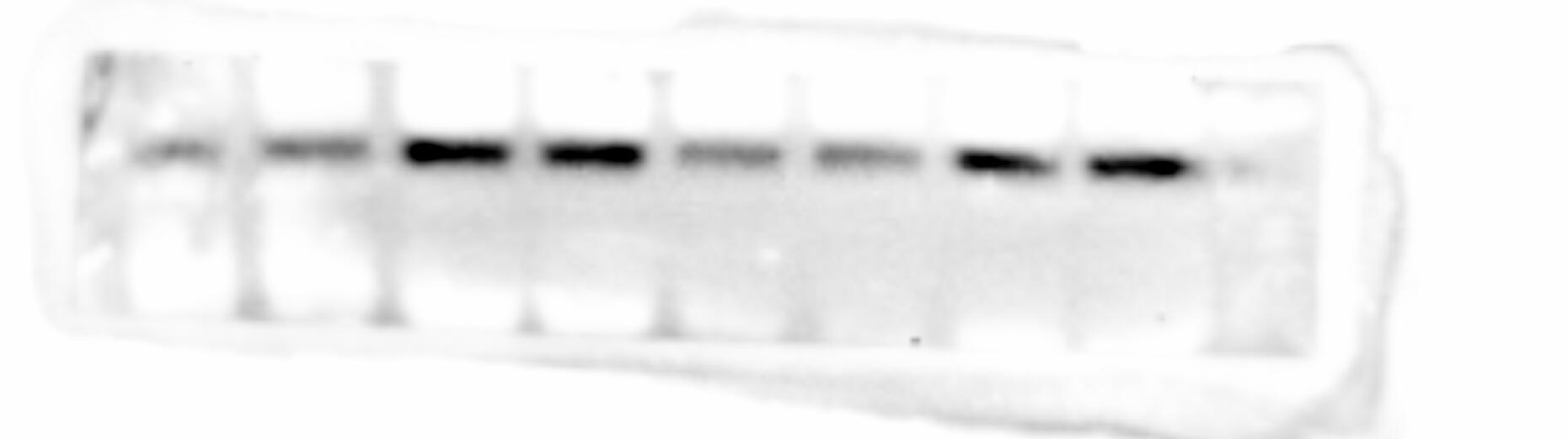

Supplement: Supplementary file 1 [file DataSheet1.ZIP › Additinal files/Western blots/Figure 6.G Western blots/Figure 6.G p-LKB1.jpg]

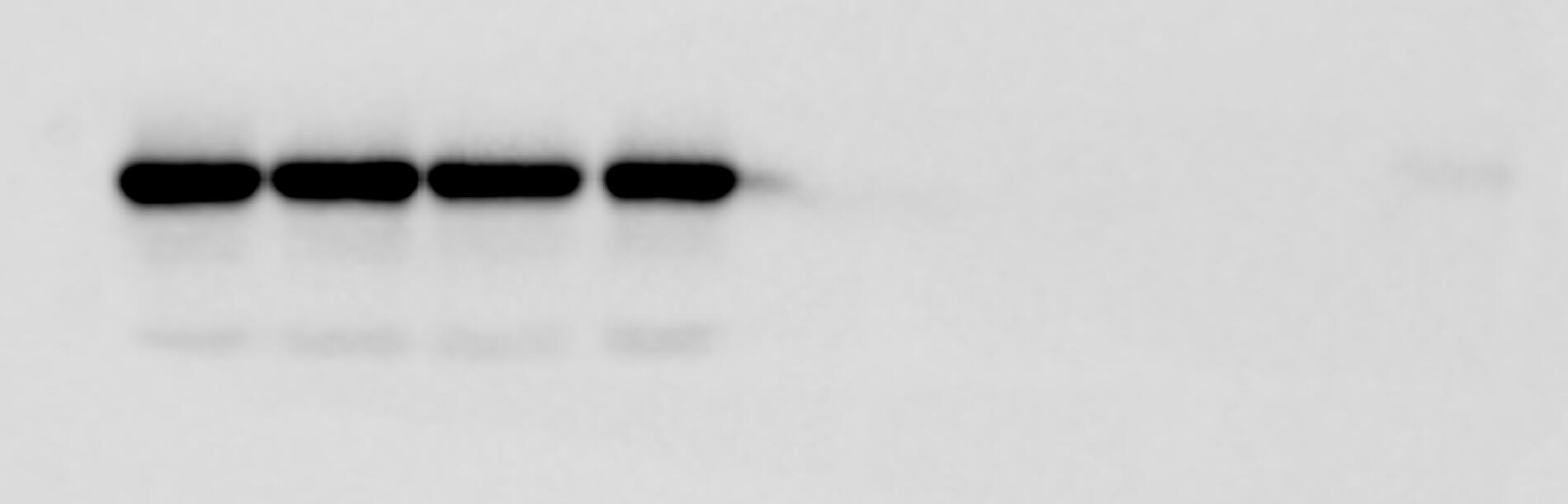

Supplement: Supplementary file 1 [file DataSheet1.ZIP › Additinal files/Western blots/Figure 6.G Western blots/Figure 6.G T-AMPKa2.jpg]

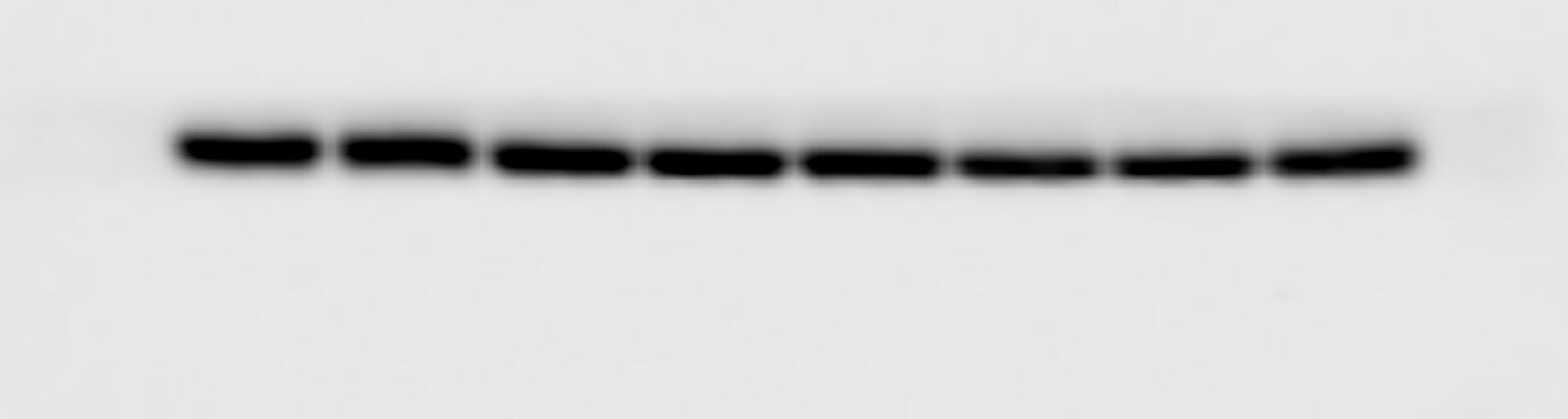

Supplement: Supplementary file 1 [file DataSheet1.ZIP › Additinal files/Western blots/Figure 6.G Western blots/Figure 6.G T-LKB1.jpg]

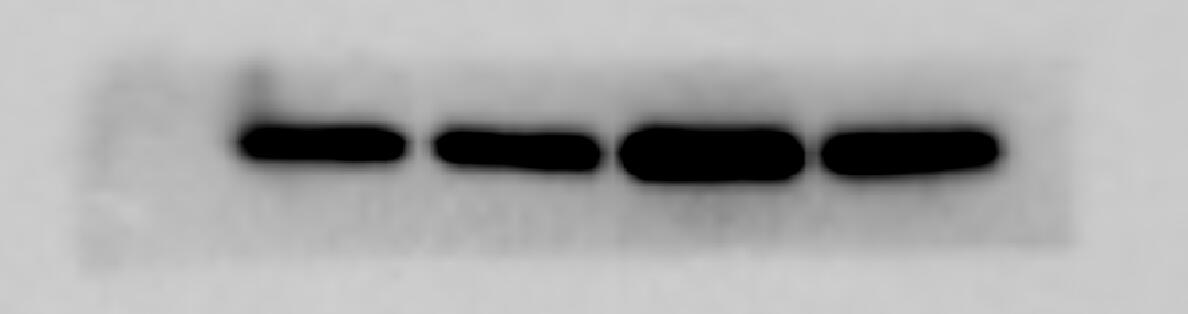

Supplement: Supplementary file 1 [file DataSheet1.ZIP › Additinal files/Western blots/Figure 7.G Western blots/Figure 7.G Bax.jpg]

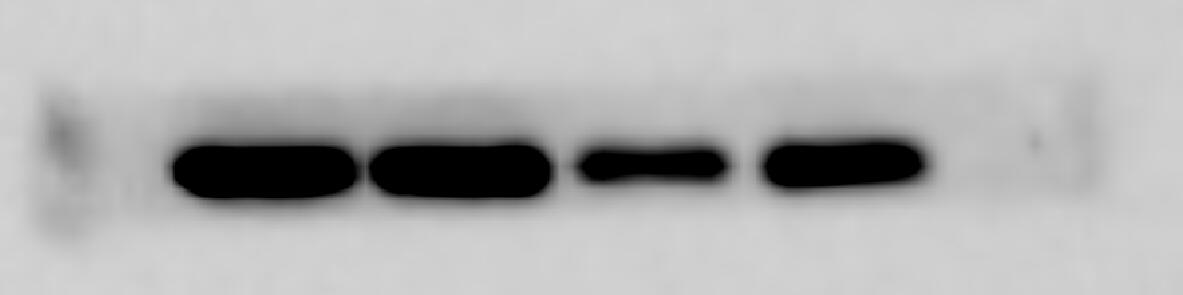

Supplement: Supplementary file 1 [file DataSheet1.ZIP › Additinal files/Western blots/Figure 7.G Western blots/Figure 7.G Bcl-2.jpg]

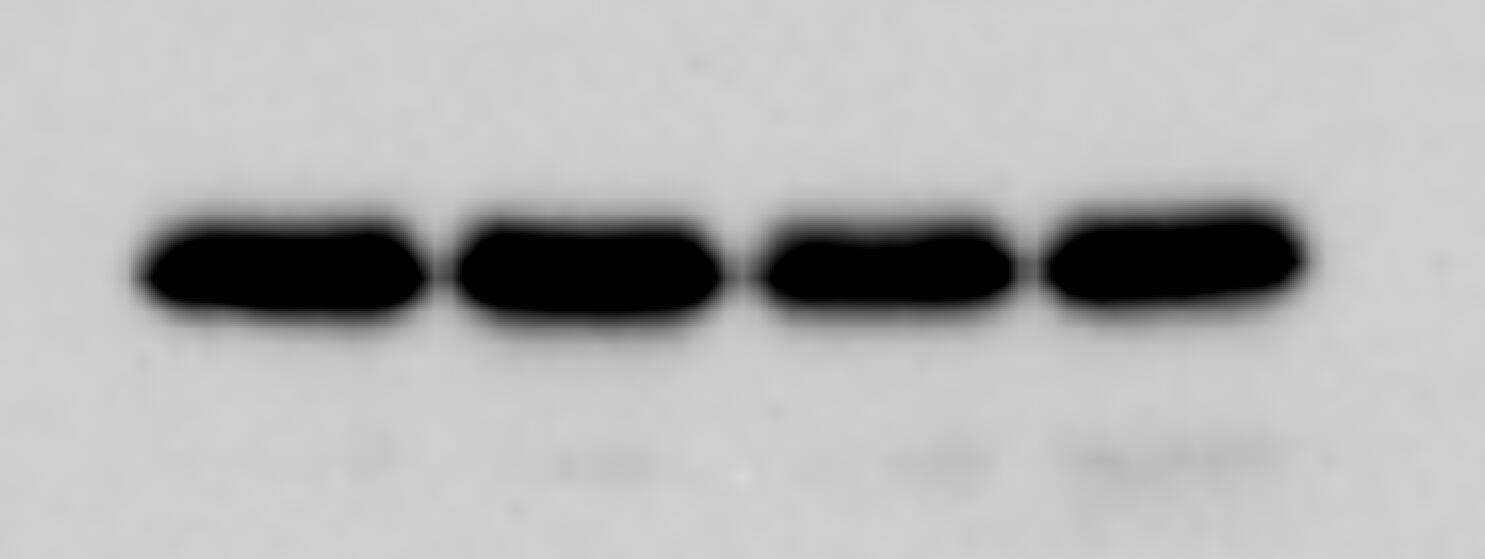

Supplement: Supplementary file 1 [file DataSheet1.ZIP › Additinal files/Western blots/Figure 7.G Western blots/Figure 7.G GAPDH.jpg]

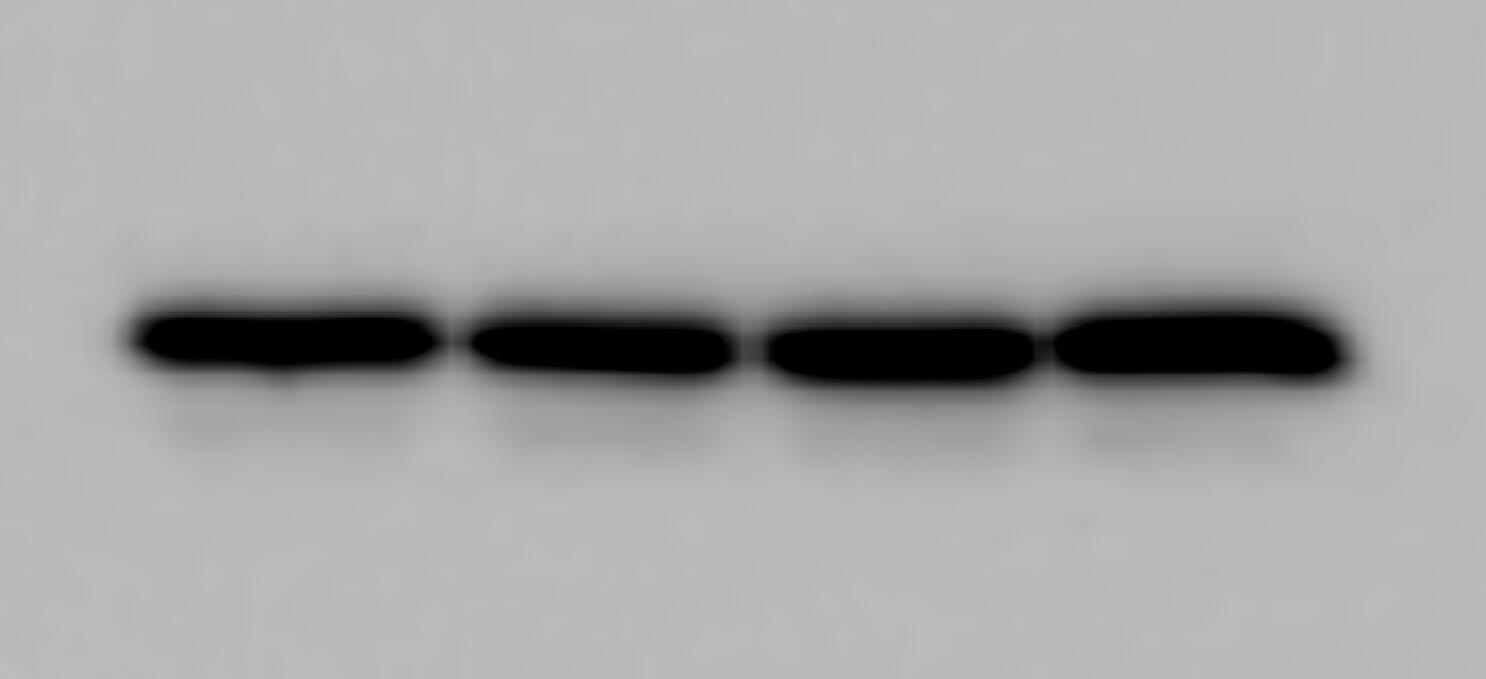

Supplement: Supplementary file 1 [file DataSheet1.ZIP › Additinal files/Western blots/Figure 7.J Western blots/Figure 7.J GAPDH.jpg]

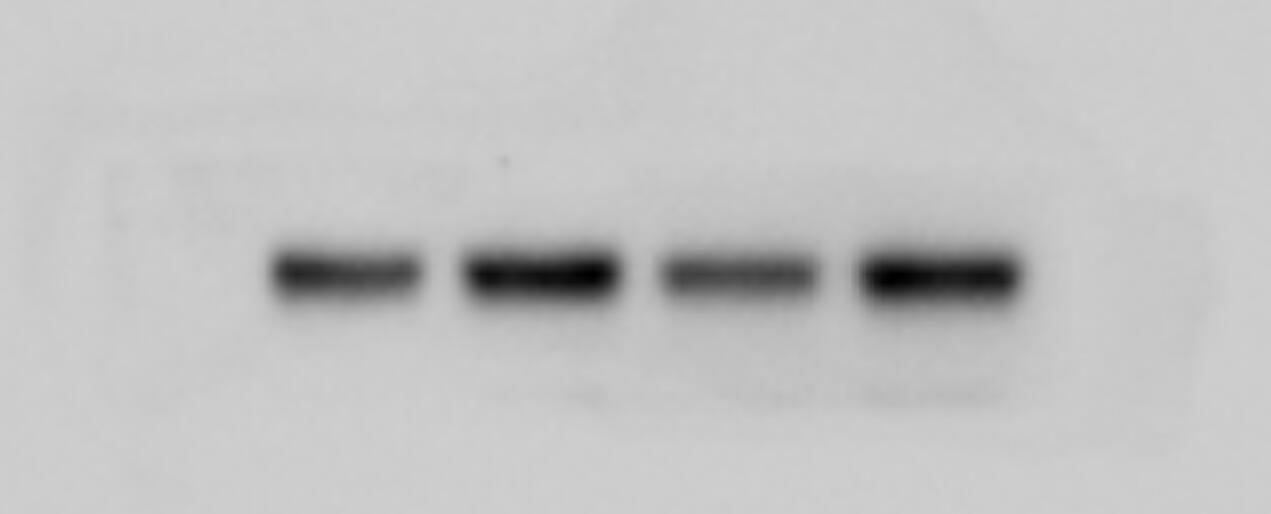

Supplement: Supplementary file 1 [file DataSheet1.ZIP › Additinal files/Western blots/Figure 7.J Western blots/Figure 7.J p-ACC.jpg]

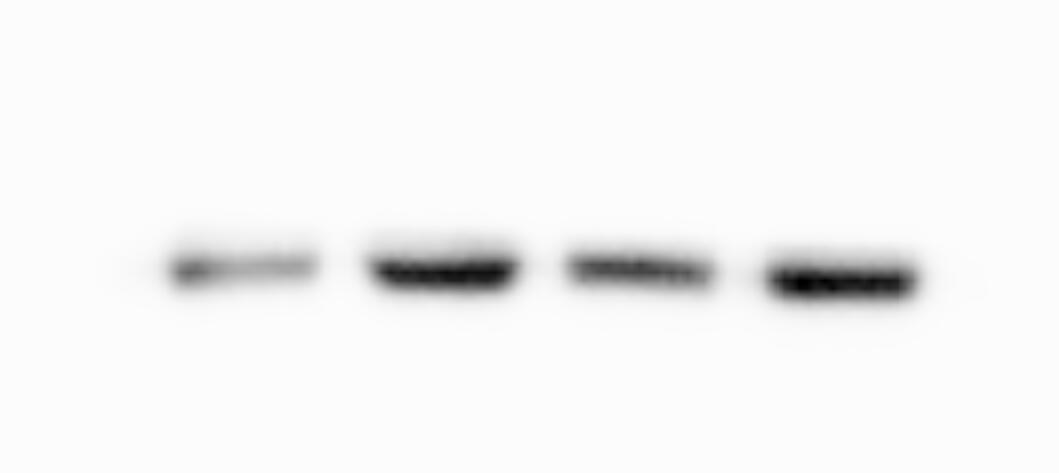

Supplement: Supplementary file 1 [file DataSheet1.ZIP › Additinal files/Western blots/Figure 7.J Western blots/Figure 7.J p-AMPKa2.jpg]

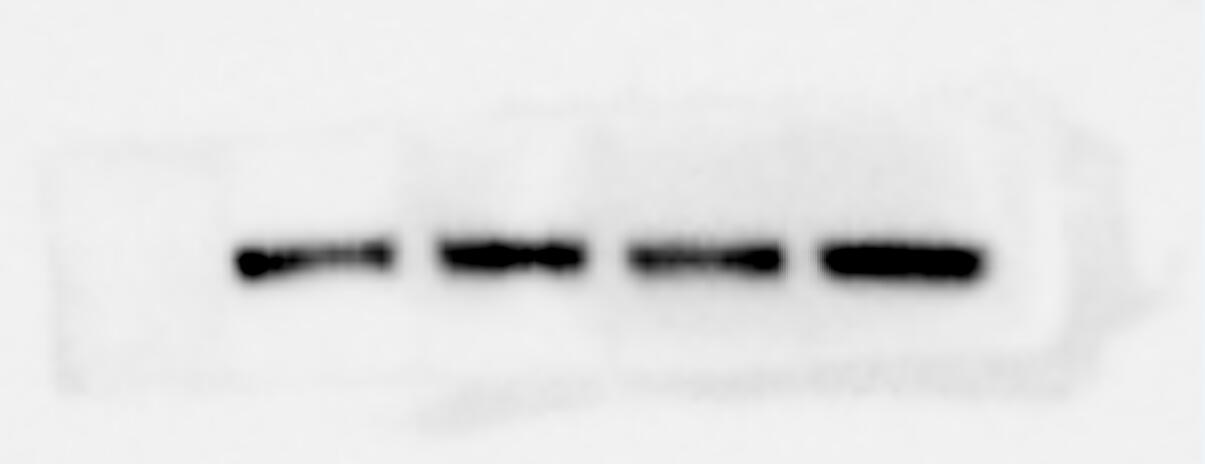

Supplement: Supplementary file 1 [file DataSheet1.ZIP › Additinal files/Western blots/Figure 7.J Western blots/Figure 7.J p-LKB1.jpg]

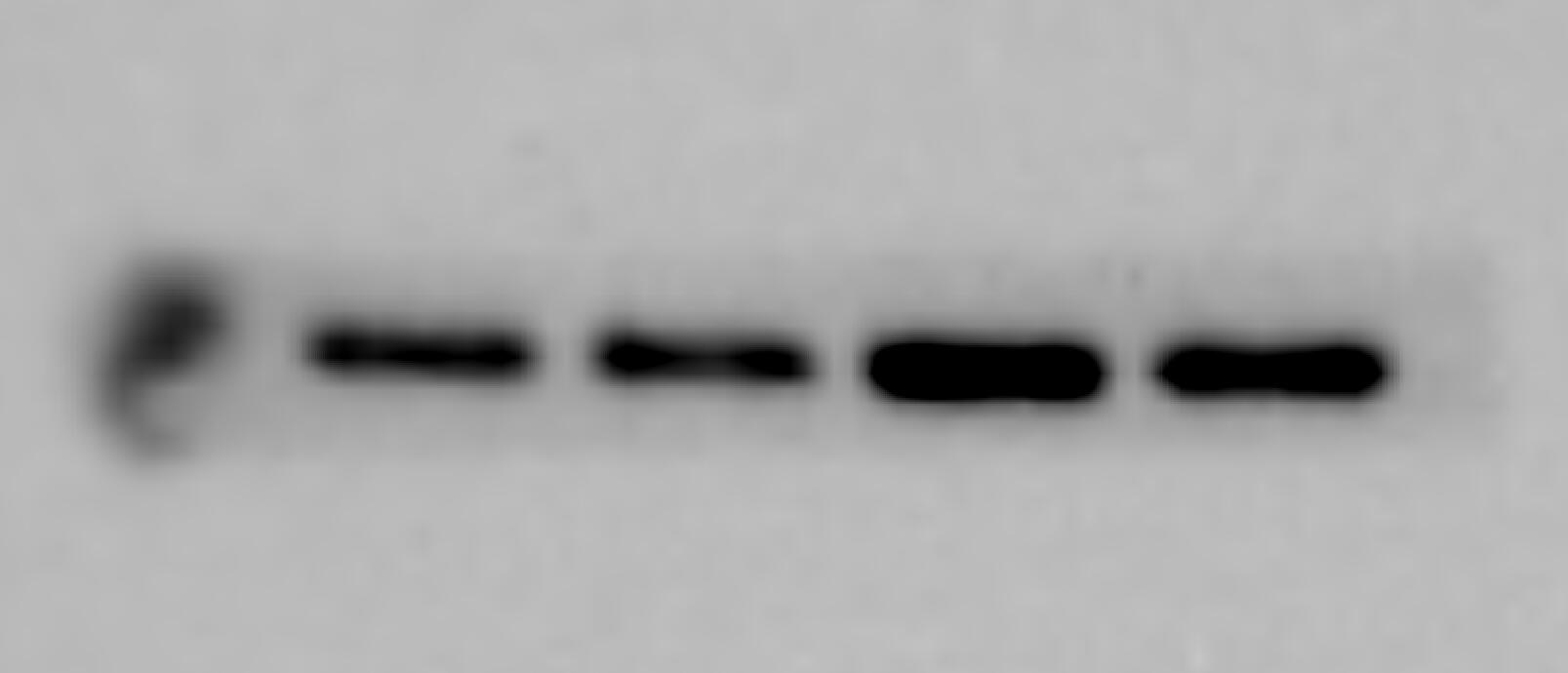

Supplement: Supplementary file 1 [file DataSheet1.ZIP › Additinal files/Western blots/Figure 7.J Western blots/Figure 7.J p-mTORC1.jpg]

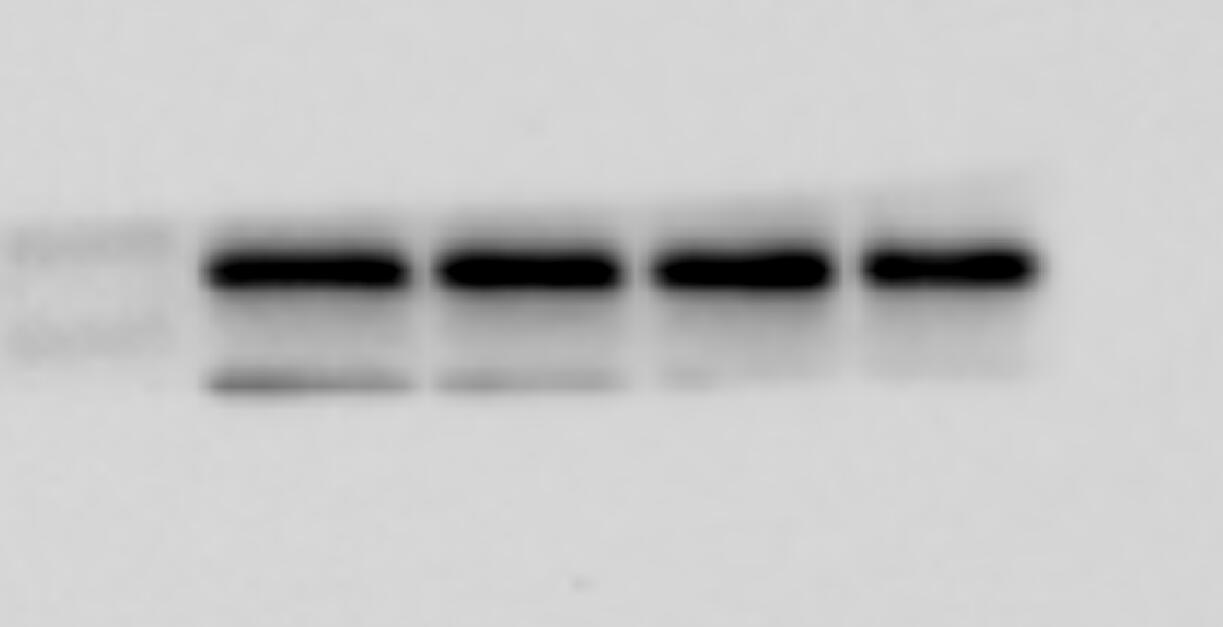

Supplement: Supplementary file 1 [file DataSheet1.ZIP › Additinal files/Western blots/Figure 7.J Western blots/Figure 7.J T-ACC.jpg]

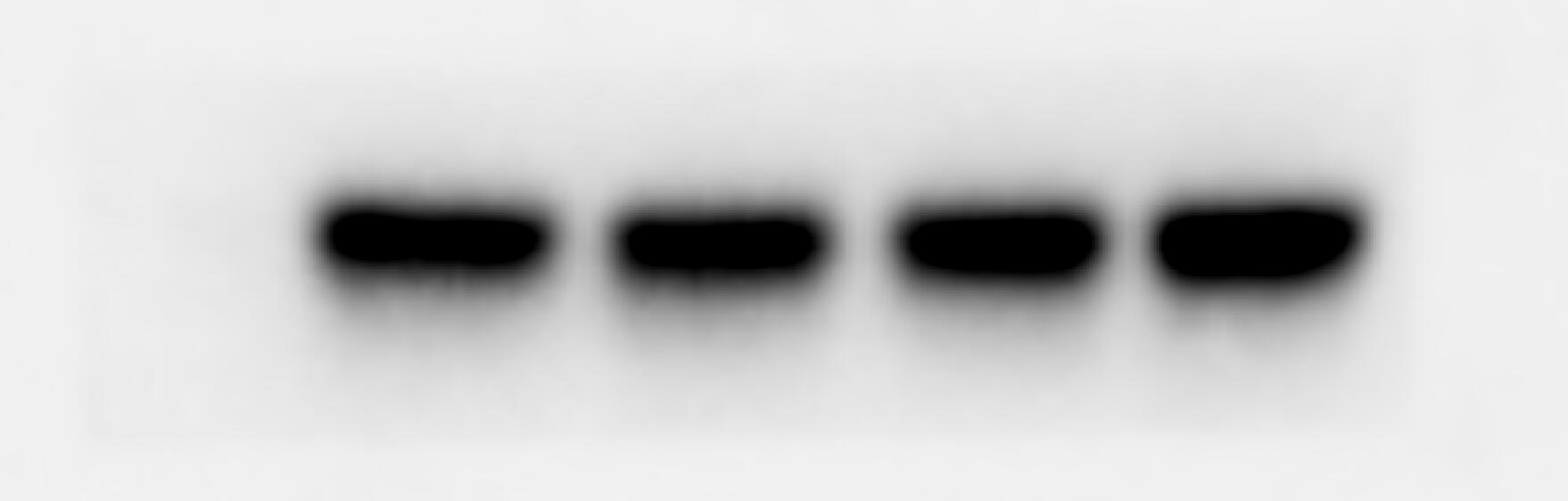

Supplement: Supplementary file 1 [file DataSheet1.ZIP › Additinal files/Western blots/Figure 7.J Western blots/Figure 7.J T-AMPKa2.jpg]

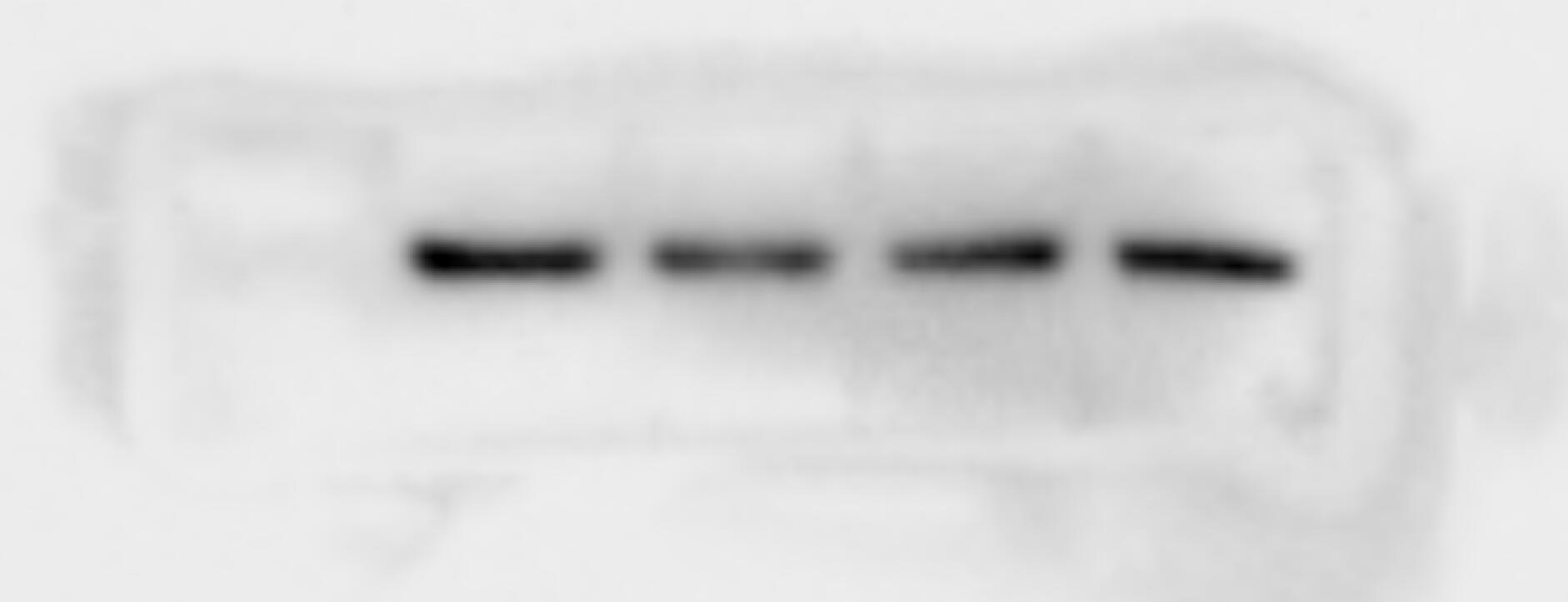

Supplement: Supplementary file 1 [file DataSheet1.ZIP › Additinal files/Western blots/Figure 7.J Western blots/Figure 7.J T-LKB1.jpg]

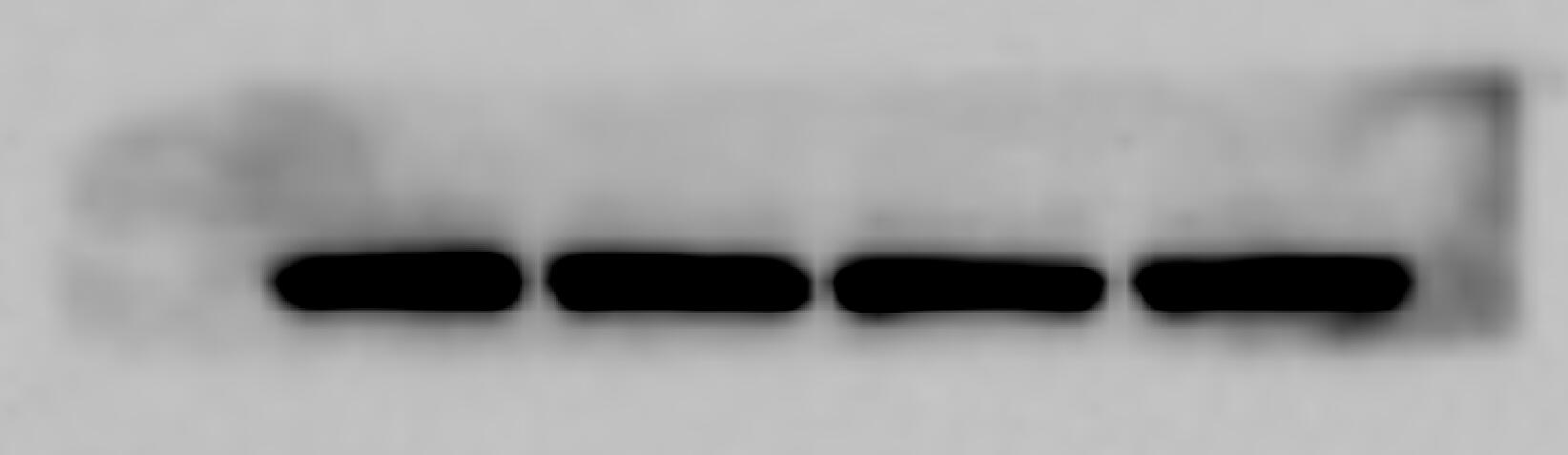

Supplement: Supplementary file 1 [file DataSheet1.ZIP › Additinal files/Western blots/Figure 7.J Western blots/Figure 7.J T-mTORC1.jpg]

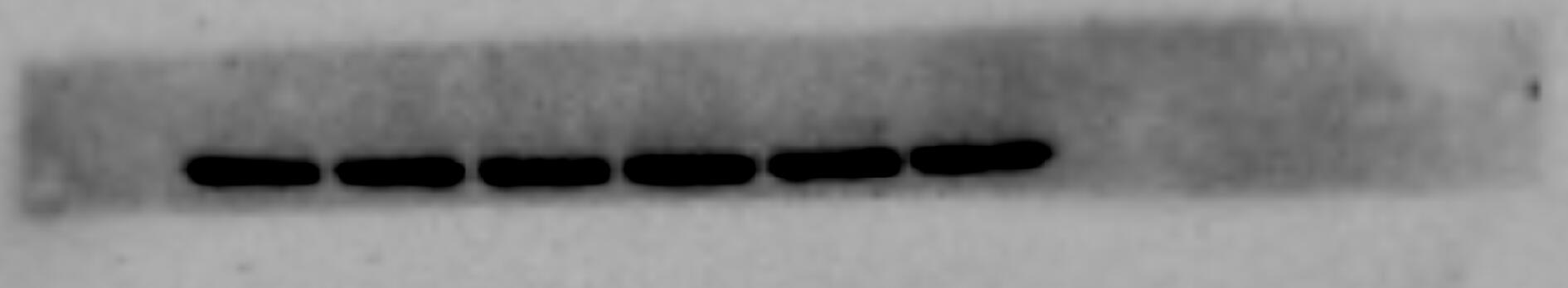

Supplement: Supplementary file 1 [file DataSheet1.ZIP › Additinal files/Western blots/Figure 8.D Western blots/Figure 8.D GAPDH.jpg]
